# Electrophilic bis-fluorophosphonium dications: Lewis acid catalysts from diphosphines†
†Electronic supplementary information (ESI) available: Preparative, spectroscopic and analytical details for the experiments described herein. CCDC 1041558–1041565. For ESI and crystallographic data in CIF or other electronic format see DOI: 10.1039/c5sc00051c



**DOI:** 10.1039/c5sc00051c

**Published:** 2015-01-27

**Authors:** Michael H. Holthausen, Rashi R. Hiranandani, Douglas W. Stephan

**Affiliations:** a Department of Chemistry , University of Toronto , 80 St. George St , Toronto , Ontario M5S3H6 , Canada . Email: dstephan@chem.utoronto.ca; b Chemistry Department-Faculty of Science , King Abdulaziz University , Jeddah 21589 , Saudi Arabia

## Abstract

Stepwise oxidation of 1,8-bis(diphenylphosphino)naphthalene and a series of (oligo)methylene-linked diphosphines with XeF_2_ followed by fluoride abstraction yields a family of compounds featuring phosphine, phosphonium and phosphorane moieties in close proximity. The bisphosphonium ions [(C_10_H_6_)(Ph_2_PF)_2_]^2+^ (**5**) and [CH_2_(Ph_2_PF)_2_]^2+^ (**9a**) exhibit remarkable Lewis acidity arising from the proximity of the phosphonium centers. The effectiveness of bisphosphonium dications (**5**, **9a–e**) is examined in a series of Lewis acid catalysed transformations.

## Abstract 2

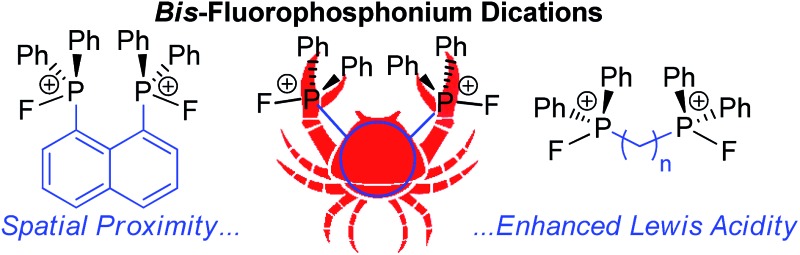
A series of electrophilic bis-fluorophosphonium dications dervied from diphosphines with naphthalene- and (oligo)methylene-linkers is presented. The resulting Lewis acidity is demonstrated to depend on the spatial proximity between the P moieties as evidenced in several Lewis acid catalyzed transformations.

## Introduction

Main group based Lewis acids are used as co-catalysts in polymerization catalysis or as Lewis acid catalysts in a wide range of organic transformations.^1^ As such reactivity is predicated only on being electron deficient, group XIII compounds such as BH_3_, BF_3_, or AlMe_3_ are the quintessential examples of main-group Lewis acids. Group XIV-centered Lewis acids (C,^2^ Si^3^) have also been exploited although as these compounds are generally very strong Lewis acids and coordinatively unsaturated, they tend to be employed as stoichiometric reagents. Moving further to the right to group XV, derivatives of these elements are most commonly Lewis bases and the electrophilic character of group XV species has drawn lesser attention. Nonetheless, P(iii) phosphenium ions [R_2_P]^+^ have been shown to be ambiphilic effecting C–C, C–H^4^ and P–P^5^ bond activation. P(v) phosphonium cations of the form [R_4_P]^+^ (R = aryl, alkyl) are weakly Lewis acidic and have been exploited as fluoride ion sensors^6^ or as catalysts for (cyclo)addition reactions to polar or activated unsaturates.^7^ Exploring more electron deficient phosphonium cations, we found that [(C_6_F_5_)_3_PF]^+^ is a highly electrophilic phosphonium cation (EPC). This Lewis acidity is attributed to the presence of the strongly electron-withdrawing substituents and the energetically accessible σ*(P–F) orbital.^8^ In an alternative approach towards EPCs, the dicationic phosphonium salt [(SIMes)PPh_2_F][B(C_6_F_5_)_4_]_2_ ^9^ was shown to be even more Lewis acidic than [(C_6_F_5_)_3_PF]^+^, demonstrating the impact of the additional positive charge. Nonetheless both [(C_6_F_5_)_3_PF]^+^ and [(SIMes)PPh_2_F]^2+^ were shown to be effective catalysts for the hydrodefluorination of fluoroalkanes and the hydrosilylation or transfer-hydrogenation of olefins and alkynes.^8–10^

In group XIII^11^ and group XIV chemistry,^12^ the proximity of two Lewis acidic centers has been shown to enhance the Lewis acidity, providing even more potent Lewis acids and thus impact on reactivity. Only recently, a group XV-based bidentate stiborane was introduced as strong, chelating Lewis acid for fluoride complexation.^13^ Exploring these notions in phosphonium chemistry prompted us to target bidentate bis-phosphonium salts. In this manuscript we prepare a series of such compounds exploiting a naphthalene backbone which locks two P moieties in the close vicinity of the *peri*-positions. In addition a series of more flexible (oligo)methylene-linked derivatives ((CH_2_)_*n*_, *n* = 1–5) are prepared. The impact of the proximity of two EPC centers is gauged by an examination of these species in a series of Lewis acid catalyses.

## Results and discussion

**Scheme 1:**
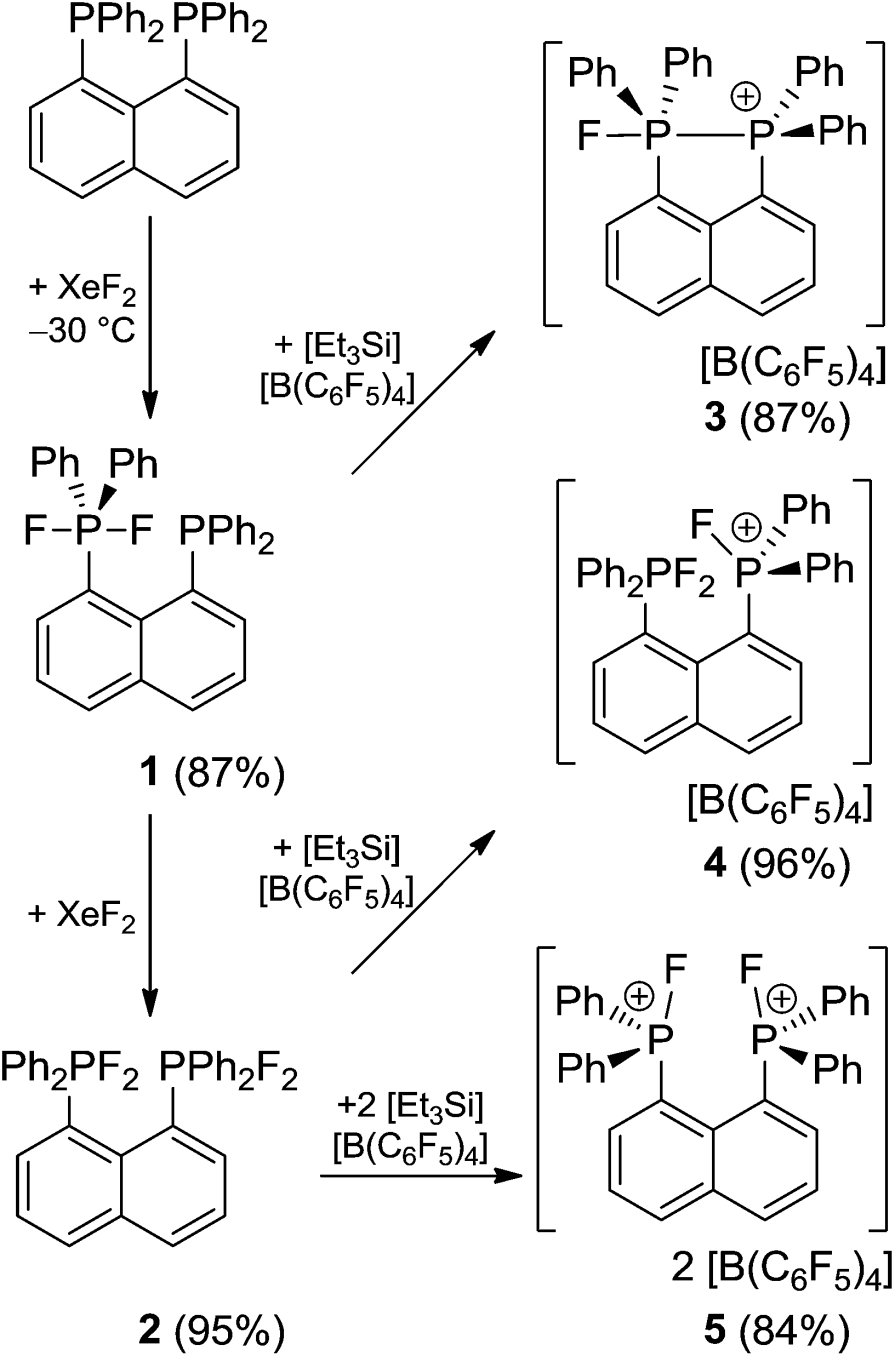
Synthetic routes to **1–5**.

The naphthalene-based diphosphine (C_10_H_6_)(PPh_2_)_2_ reacts with XeF_2_ at low temperatures (–30 °C) affording the selective formation of the phosphine–phosphorane species (C_10_H_6_)(Ph_2_PF_2_)(PPh_2_) (**1**) in high yields (87%, Scheme 1). The ^31^P{^1^H} NMR spectrum of **1** shows two triplet of doublet resonances at –55.5 ppm (^1^ *J*_PF_ = 742 Hz, ^4^ *J*_PP_ = 10 Hz) and –18.5 ppm (^5^ *J*_PF_ = 17 Hz, ^4^ *J*_PP_ = 10 Hz) which are assigned to the phosphorane and phosphine moieties respectively.^14^ The associated doublet of doublet resonance of the fluorine atoms shows a chemical shift of –42.0 ppm in the ^19^F{^1^H} NMR spectrum.

Reacting (C_10_H_6_)(PPh_2_)_2_ with XeF_2_ in a 1 : 2 stoichiometry at ambient temperature yields quantitatively the bis-difluorophosphorane (C_10_H_6_)(Ph_2_PF_2_)_2_ (**2**, 95%). An AA′X_2_X′2 spin system is observed in the ^31^P{^1^H} (*δ*_A_ = –49.4 ppm) and ^19^F{^1^H} NMR (*δ*_X_ = –34.3 ppm) spectra of **2** and its parameters were evaluated by full line shape iteration.^15^ Both resonances of **2** are shifted to lower field compared to the phosphorane moiety in **1**. A relatively large ^5^ *J*_PF_ (^5^ *J*_A′X_ = ^5^ *J*_AX′_ = –18 Hz) and a comparatively small ^4^ *J*_PP_ coupling constant (^4^ *J*_AA′_ = 3 Hz) might indicated a through space interaction between both phosphonium moieties.^16^

**Fig. 1:**
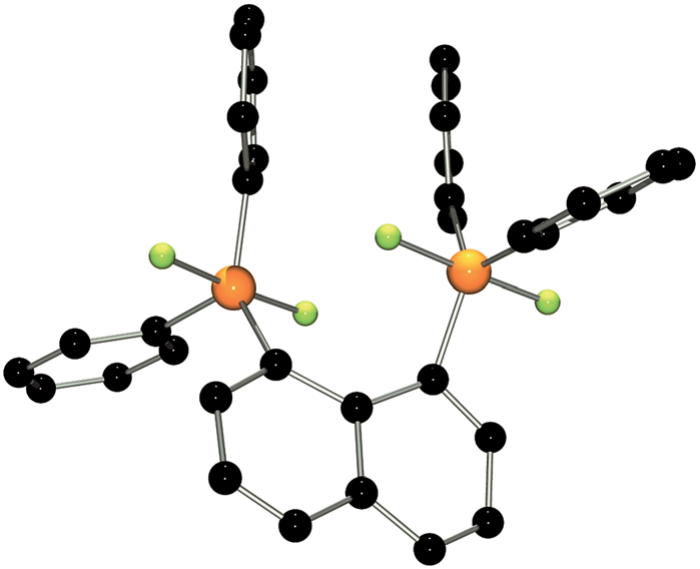
POV-ray depiction of **2**. P: orange, F: yellow-green, C: black. Hydrogen atoms are omitted for clarity.

The molecular structure of **2** shows two trigonal bipyramidal phosphorus moieties with fluorine atoms occupying the axial positions (*av.* F–P–F: 177.0(1)°, Fig. 1). The P–F bond length (*av.* 1.67(3) Å) is typical for difluorotriarylphosphoranes.^14,17^ The close proximity of the phosphorane moieties in **2** leads to significant steric strain. This results in a buckling of its naphthalene-backbone as evidenced by the mean C-atom deviation from the least-squares C_10_ plane of 0.120(2) Å. The corresponding deviation in the parent diphosphine (C_10_H_6_)(Ph_2_P)_2_ is 0.006 Å.^18^ The steric strain also causes the phosphorus moieties of **2** to exhibit a strong bending out of the naphthalene plane (*av.* 0.73(1) Å *vs.* (C_10_H_6_)(Ph_2_P)_2_ *av.* 0.42 Å). The shortest P···F interactions between the phosphorane moieties is 2.825(4) Å which is well within the sum of the respective van der Waal's radii (PF: 3.27 Å).^19^ The corresponding P···P distance of 3.823(3) Å is significantly longer than that in the parent diphosphine (3.052 Å).^18^ Earlier reports have described difficulties in effecting the quaternisation of both P atoms in (C_10_H_6_)(Ph_2_P)_2_ due to steric congestion,^20^ and, to the best of our knowledge, **2** represents the first compound featuring two penta-coordinated P atoms in *peri*-positions on a naphthalene framework.

**Fig. 2:**
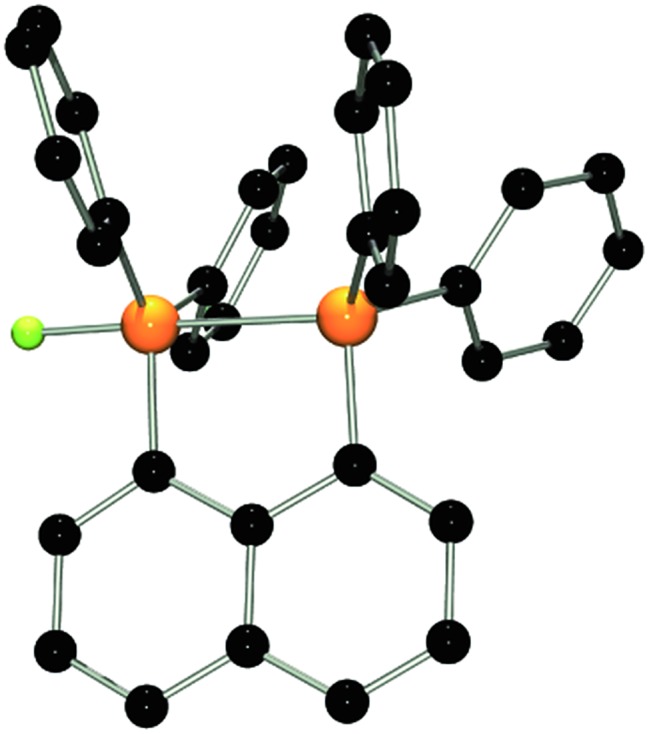
POV-ray depiction of the cation in **3**. P: orange, F: yellow-green, C: black. Hydrogen atoms are omitted for clarity.

The reactions of phosphoranes **1** and **2** with [Et_3_Si][B(C_6_F_5_)_4_]·2(C_7_H_8_) in 1 : 1 stoichiometries yielded the respective salts [(C_10_H_6_)(Ph_2_PF)(PPh_2_)][B(C_6_F_5_)_4_] (**3**) and [(C_10_H_6_)(Ph_2_PF_2_)(Ph_2_PF)][B(C_6_F_5_)_4_] (**4**) in good to excellent yields (Scheme 1). The ^31^P{^1^H} NMR spectrum of **3** shows doublet of doublet resonances at –17.4 and –5.5 ppm. The former is assigned to the Ph_2_PF-moiety on basis of its ^1^ *J*_PF_ value of 783 Hz. A very large coupling constant between both P atoms (138 Hz) indicates coordination of the lone pair of the phosphine-moiety to the fluorophosphonium centre.^21^ This also results in a large coupling constant between the fluorine atom and the distal P atom (164 Hz; *vs.* ^5^ *J*_PF_ = 17 Hz in **1**). The indicated PP interaction was confirmed by single crystal X-ray crystallography (Fig. 2) as the P–P bond length was found to be 2.530(1) Å. This is longer than the P–P distance in P_4_ (2.2 Å)^22^ indicating that the interaction in **3** is weak. The fluoro-substituted P atom shows a trigonal bipyramidal bonding environment with the fluorine and the coordinating phosphine-moiety adopting axial positions. The P–F bond distance (1.637(2) Å) is significantly longer than in other fluorophosphonium species (*av.* 1.54(1) Å)^8,9,14^ which is consistent with the donation of electron density from the phosphine unit into the σ*-orbital of the P–F bond. In contrast to **2**, the naphthalene backbone of **3** show no evidence of steric strain (mean C-atom deviation from the least-squares C_10_ plane: 0.006(1) Å).

It is noteworthy that interactions between the P atoms of adjacent phosphine and alkyl or aryl phosphonium centers in the related cations [(C_10_H_6_)(Ph_2_PR)(Ph_2_P)]^+^ (R = alkyl, aryl) is not observed,^17,20a,23^ reflecting the Lewis acidity of the fluorophosphonium cation. While we have previously reported the intramolecular coordination of an amide fragment to a fluorophosphonium moiety,^24^ compound **3** appears to be the first example of phosphine coordinating to a fluoro-phosphonium center. It is important to note that such an interaction is not observed in a 1 : 1 mixture of Ph_3_P and [Ph_3_PF][B(C_6_F_5_)_4_] indicating that the close proximity between Lewis acid and base imposed by the naphthalene backbone is crucial for adduct formation.

**Fig. 3:**
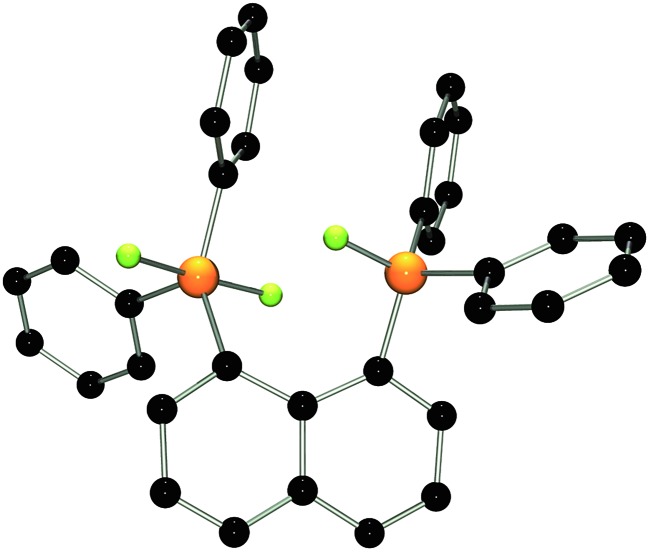
POV-ray depiction of the cation in **4**·(CH_2_Cl_2_). P: orange, F: yellow-green, C: black. Hydrogen atoms are omitted for clarity. The solvate molecule was removed by the squeeze routine of the program PLATON.

The ^31^P{^1^H} NMR spectrum of [(C_10_H_6_)(Ph_2_PF_2_)(Ph_2_PF)]^+^ (**4**) shows a broad triplet resonance at –51.7 ppm (^1^ *J*_PF_ = 717 Hz) assigned to the phosphorane moiety and a broad doublet at 96.6 ppm (^1^ *J*_PF_ = 1012 Hz) corresponding to the phosphonium centre. A crystallographic study revealed that the P–F bond lengths at the penta-coordinated P atom of **4** (*av.* 1.664(2) Å) are longer than the P–F bond distance at the adjacent fluorophosphonium centre (1.543(1) Å; Fig. 3). Similar to **2**, a weak P···F interaction between the F on the phosphonium and P of the phosphorane fragment is observed (2.995(2) Å). Compared to **2** there is a decreased buckling of the naphthalene moiety (mean C-atom deviation from the least-squares C_10_ plane: 0.097(1) Å), a shorter P···P distance (3.668(2) Å), and the phosphonium P atom in **4** shows a reduced bending out of the naphthalene plane (0.057(1) Å) compared to its phosphorane neighbour (0.076(1) Å).

The bis-difluorophosphorane **2** also reacts with two equivalents of [Et_3_Si][B(C_6_F_5_)_4_]·2(C_7_H_8_) to give the salt [(C_10_H_6_)(Ph_2_PF)_2_][B(C_6_F_5_)_4_]_2_ (**5**) in high yields (84%, Scheme 1). The ^31^P{^1^H} and ^19^F{^1^H} NMR spectra of **5** reveal an AA′XX′ spin system. The ^19^F{^1^H} NMR spectrum reveals a very broad doublet resonance (*δ*_X_ = –117.1 ppm, Δ*ν*_1/2_ = 450 Hz). The ^31^P{^1^H} NMR resonance (*δ*_A_ = 96.8 ppm) shows a relatively large ^1^ *J*_PF_ coupling constant (^1^ *J*_AX_ = ^1^ *J*_A′X′_ = 1004 Hz) which has also been observed for the related cations [(C_6_F_5_)_3_PF]^+^ (1062 Hz),^8^ and [(SIMes)PPh_2_F]^2+^ (1040 Hz ^9^). Similar to **2**, dication **5** also features a relative large ^5^ *J*_PF_ (^5^ *J*_A′X_ = ^5^ *J*_AX′_ = –13 Hz) and a comparatively small ^4^ *J*_PP_ coupling constant (^4^ *J*_AA′_ = 3 Hz). The related doubly protonated naphthalene-based bis-phosphine was previously studied by NMR spectroscopy.^25^ While dications incorporating a P–P bond^21b,26^ or a bridging substituent^23,27^ have been previously isolated.

**Scheme 2:**
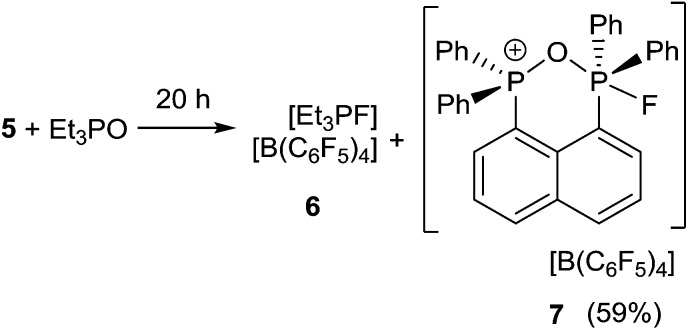
Reaction of **5** with Et_3_PO.

In an effort to probe the Lewis acidity of **5**, it was reacted with an equivalent of Et_3_PO employing the Gutmann–Beckett method (Scheme 2).^28^ The ^31^P{^1^H} NMR spectrum of the reaction mixture shows a broad resonance at 60.5 ppm corresponding to Et_3_PO interacting with **5**. This results in an acceptor number of 21.4, which suggests that **5** is approximately half as Lewis acidic as B(C_6_F_5_)_3_. In comparison, combination of the salt [Ph_3_PF][B(C_6_F_5_)_4_] and Et_3_PO in a 2 : 1 stoichiometry showed only a very small shift in the resonance of Et_3_PO. These observations infer that the Lewis acidity of a phosphonium center in **5** is enhanced by the proximity of a second fluorophosphonium moiety. Monitoring the 1 : 1 mixture of **5** and Et_3_PO over time revealed slow transformation to [Et_3_PF]^+^ (**6**)^9^ and [(C_6_H_10_)(Ph_2_PO)(Ph_2_PF)]^+^ (**7**). The ^31^P{^1^H} NMR spectrum of (**7**) shows the expected doublet of doublet resonances at –34.5 ppm (^1^ *J*_PF_ = 697 Hz, *J*_PP_ = 23 Hz) and 47.1 ppm (*J*_PF_ = 2 Hz). The high field shift of the fluorophosphonium moiety and the low field shift of the phosphine oxide indicate some degree of donation of electron density from the PO fragment to the electrophilic fluorophosphonium cation. A similar but faster fluoride oxide exchange reaction was previously reported for the more Lewis acidic dication [(SIMes)PPh_2_F]^2+^.^9^

**Scheme 3:**
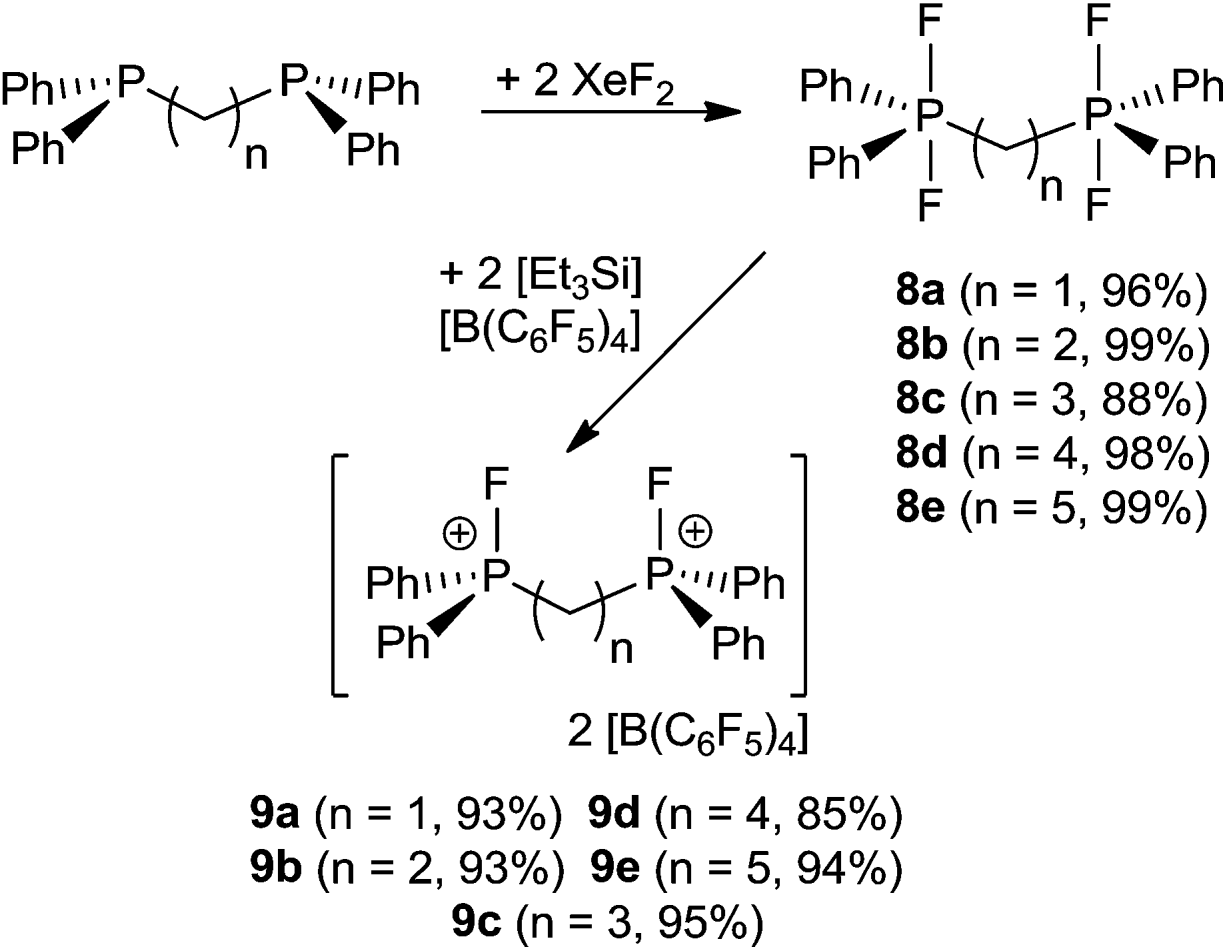
Synthetic route to **8a–e** and **9a–e**.

A family of related bis-phosphonium dication salts was envisioned and thus reactions of (oligo)methylene-bridged diphosphines (CH_2_)_*n*_(Ph_2_P)_2_ (*n* = 1 (a), 2 (b), 3 (c), 4 (d), 5 (e)) and XeF_2_ were performed in CH_2_Cl_2_ to yield the respective bis-difluorophosphoranes (CH_2_)_*n*_(Ph_2_PF_2_)_2_ (**8a–e**) in high to almost quantitative isolated yields (Scheme 3). The species **8a–d** have been previously prepared by using a range of oxidation reagents including F_2_,^29^ SF_4_,^30^ Me_2_NSF_3_,^31^ (CF_3_)_2_CO^32^ COF_2_ ^33^ and NF_3_O^34^ or by halide exchange from the respective bromophosphoranes^30^ or by hydrofluorination of the respective silyl phosphinimines with HF.^35^ However, most of these methods suffer from low yields, laborious workup procedures and the use of hazardous reagents. The reaction of phosphoranes **8a–e** with two equivalents of [Et_3_Si][B(C_6_F_5_)_4_]·2(C_7_H_8_) resulted in the formation of corresponding bis-phosphonium dication salts **9a–e** (Scheme 3). All five derivatives were isolated and fully characterized. It is curious to note that the salts **9b** and **9d**, featuring linkers with an even number of CH_2_ units, are only sparingly soluble in CD_2_Cl_2_ whereas those with an odd numbers of methylene carbons in the linker (**9a**, **9c**, **9e**) show good solubility. The respective NMR spectra of **9a–e** exhibit AA′XX′ spin systems (see ESI†). Compounds **9a–c** show higher order effects due to coupling between the nuclei of the magnetically inequivalent phosphonium moieties and thus the NMR parameters were obtained *via* a full line shape iteration procedure.^15^ Interestingly **9a** exhibits a large P–F coupling constant (^1^ *J*_AX_ = 1024 Hz) and significant coupling between both fluorine atoms (^4^ *J*_XX′_ = 12 Hz) suggesting enhanced Lewis acidity. Remarkable large P–P coupling constant was observed for **9b** (^3^ *J*_AA′_ = 70 Hz) suggesting a weak through space interaction between the phosphorus centers (see ESI†).^36^

**Scheme 4:**
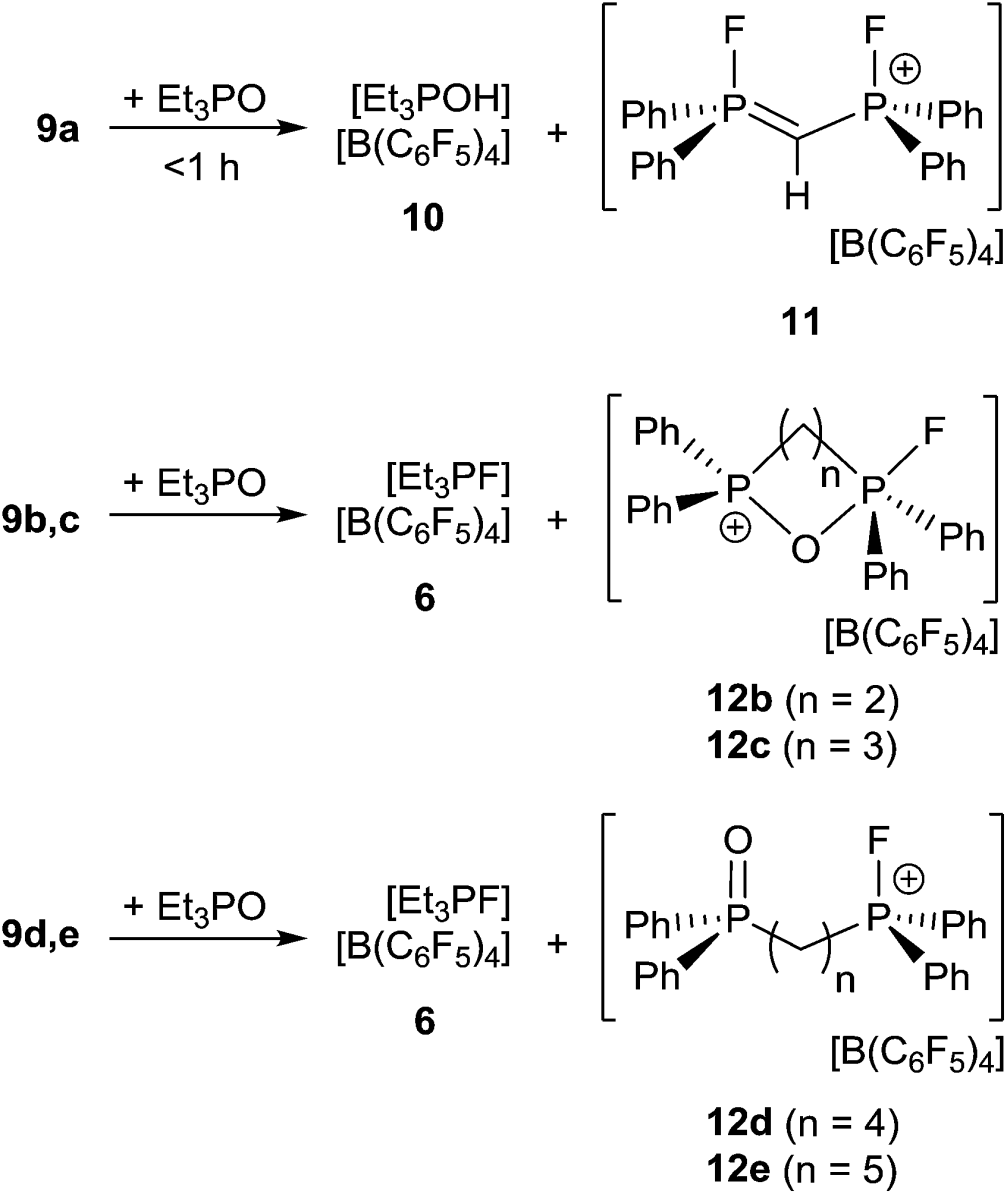
Reaction of **9a–e** with Et_3_PO.

Lewis acidity tests of this series of bis-fluorophosphonium ion salts **9a–e** by the Gutmann–Beckett method^28^ were performed (Scheme 4). Determination of an acceptor number for **9a** was hampered by deprotonation of the central CH_2_ moiety, affording [Et_3_POH][B(C_6_F_5_)_4_] (**10**) and [(CH)(Ph_2_PF)_2_][B(C_6_F_5_)_4_] (**11**). Compound (**10**) showed a broad ^31^P{^1^H} NMR resonance at 74.1 ppm, while (**11**) reveals an AA′XX′ spin system in the ^31^P{^1^H} (*δ*_A_ = 81.2 ppm) and ^19^F{^1^H} NMR spectrum (*δ*_X_ = –99.4 ppm). The formation of **11** was further supported by its independent synthesis from **9a** and *t*-Bu_3_P.

The cations of **9b–e** interact weakly with Et_3_PO resulting in acceptor numbers of ∼5. However, in all cases slow conversion to the monocationic species [(CH_2_)_*n*_(Ph_2_PO)(Ph_2_PF)][B(C_6_F_5_)_4_] (**12b–e**) and [Et_3_PF]^+^-salt **6** was observed.^9^ The shorter linkers in the case of **12b** and **12c** result in a chelate interaction of the phosphine oxide and the electrophilic fluorophosphonium center as evidenced by the high field shift of the fluoro-substituted P atom (**12b**: 2.2 ppm, ^1^ *J*_PF_ = 717 Hz, **12c**: 45.2 ppm, ^1^ *J*_PF_ = 823 Hz). This notion is also supported by the observation of comparatively low field ^31^P NMR resonances arising for the PO fragments (**12b**: 53.1 ppm, **12d**: 44.1 ppm).^28^ In contrast **12d–e** show ^31^P{^1^H} NMR resonances in the typical range for discrete phosphine oxide^37^ and fluorophosphonium moieties.

**Scheme 5:**
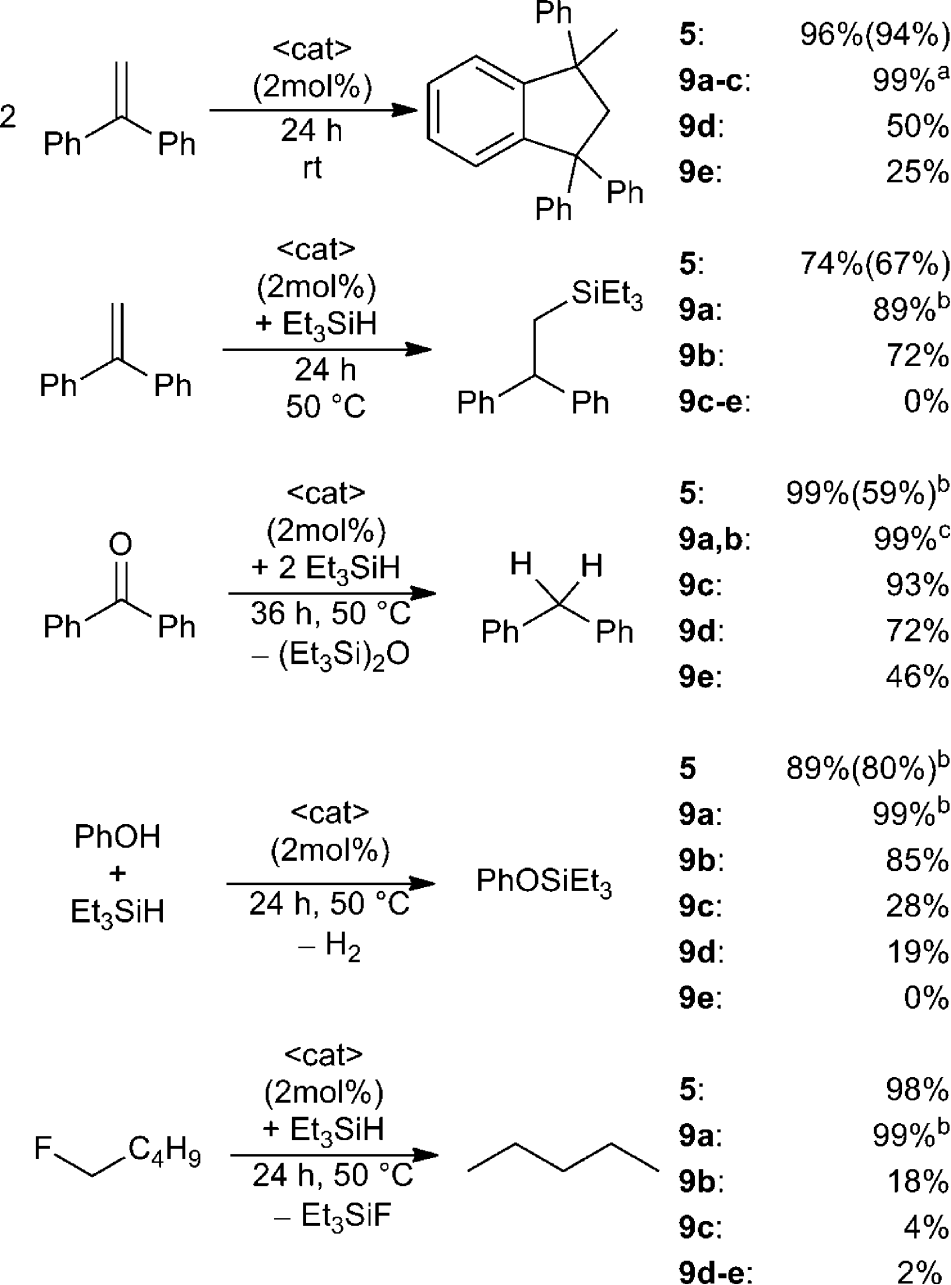
Lewis acid catalysis by salts **5** and **9a–e** as catalysts for selected Lewis acid mediated transformations. Conversions were determined by ^1^H NMR or ^19^F NMR spectroscopy and isolated yields are given in parenthesis. (a) Ambient temperature and one hour reaction time (only for **9a**). (b) Ambient temperature and one hour reaction time. (c) Ambient temperature and one hour (**9a**) or three hours (**9b**) reaction time.

The utility of the dications **5** and **9a–e** in a variety of Lewis acid catalysed reactions was probed (Scheme 5). 1,1-Diphenylethylene in CD_2_Cl_2_ was treated with 2 mol% of **5** or **9a–e**. In the case of **5** almost quantitative Friedel–Crafts dimerization was observed within 24 h at ambient temperature and the resulting product was isolated in 94% yield.^38^ Complete conversion was also achieved using **9a–c**, however, **9a** required only one hour reaction time, while **9b,c** were complete in 24 h. In the case of **9d–e** under the same conditions after 24 h, the Friedel–Crafts product was formed in lesser yields (**9d**: 50%, **9e**: 25%). The corresponding reaction of 1,1-diphenylethylene with Et_3_SiH in the presence of these Lewis acid catalysts was assessed (Scheme 5).^9,10a^ Using **9a** as the catalyst gave 89% of the hydrosilylated product in one hour at ambient temperature whereas **9b** and **5** achieve comparable yields after 24 h at 50 °C. No conversion is observed in the case of less Lewis acidic catalysts **9c–e**.

In a similar fashion treatment of benzophenone with two equivalents of Et_3_SiH in the presence of Lewis acid catalysts **5** and **9a,b** led to hydrodeoxygenation and complete conversion to diphenylmethane and Et_3_SiOSiEt_3_ after one hour (**5**, **9a**) or three hours (**9b**) respectively (Scheme 5). Compounds **9c–e** required elevated temperatures and 36 h reaction time and afforded product in decreased yields (**9c**: 93%, **9d**: 72%, **9e**: 46%).

A fourth test involved the utility of these catalysts in the dehydrocoupling of Si–H bonds in silanes with phenol (Scheme 5).^10*b*^ Compounds **5** and **9a** result in rapid dehydrogenative coupling which gives PhOSiEt_3_ in high yields within one hour at ambient temperature. In contrast, elevated temperature (50 °C) and 24 h reaction time were required to observe similar reactivity for catalyst **9b**. With an increase in methylene-linker length a decrease in conversion was observed (**9c**: 30%, **9d**: 19%) and only traces of the coupling product are detected using **9e**.

Finally, these EPCs were evaluated as catalysts for the hydrodefluorination of fluoropentane using Et_3_SiH as the hydride source (Scheme 5).^8,9^ Compounds **9a** and **5** catalyse the hydrodefluorination affording almost complete conversion after one hour at ambient temperature for (**9a**) or 24 h at 50 °C for (**5**). The less Lewis acidic species **9b–e** effect incomplete to trace conversions (**9b**: 18%, **9c**: 4%, **9d**: 2%, **9e**: 2%) after 24 h at 50 °C.

The above results reveal that these dications are generally effective catalysts for a variety or Lewis acid mediated reactions. Moreover the proximity of two fluorophosphonium centers as in **5** and **9a–b** acts to enhance the Lewis acidity and the resulting reactivity by a distal P–F interaction. Indeed, the longer chain species (**9c–e**) show diminished reactivity behaving more like independent [Ph_2_PF(alkyl)]^+^ cations. In this regard it is also noteworthy that in each of the above Lewis acid-catalysed reactions the species [Ph_3_PF][B(C_6_F_5_)_4_] was completely ineffective, which further supports the view that Lewis acidity of the present species is enhanced by spatial proximity.

## Conclusions

In summary, stepwise oxidations of 1,8-bis-(diphenylphosphino)naphthalene and a series of bidentate (oligo)methylene-linked phosphines with XeF_2_ provide mono- and bisphosphorane species. Subsequent stepwise fluoride abstraction yielded phosphine/phosphonium, phosphonium/phosphorane and bisphosphonium species. The close spatial proximity in the naphthalene derived compound and the mono-methylene-linked species gives rise to Lewis acidity that is enhanced by the neighbouring fluorophosphonium moieties as evidenced by the reactivity of these species in the Lewis acid catalysed transformations including Friedel Crafts-type dimerization, hydrosilylation, dehydrocoupling, hydrodeoxygenation and hydrodefluorination. We are continuing to explore the facile and high yielding synthetic protocol that converts readily available bidentate donors providing Lewis acids of tuneable strength. New applications in Lewis acid catalysis and applications of these P(v) Lewis acids in frustrated Lewis pair (FLP) chemistry are the subject of on-going efforts.
