# Supplementary material for: Electrophilic bis-fluorophosphonium dications: Lewis acid catalysts from diphosphines
Source: Chem Sci. 2015 Jan 27;6(3):2016–21. doi: 10.1039/c5sc00051c (PMC5496503; doi:10.1039/c5sc00051c)
Supplement: Supplementary file 1 [file SC-006-C5SC00051C-s001.pdf]

Electronic Supplementary Material (ESI) for Chemical Science.
This journal is © The Royal Society of Chemistry 2015

Electrophilic *Bis*-Fluorophosphonium Dications:

Lewis Acid Catalysts from Diphosphines

Michael H. Holthausen, Rashi R. Hiranandani and Douglas W. Stephan

Supporting Information

This PDF file includes:

**1.**	**Materials and Methods**		S4
**2.**	**Syntheses and Spectroscopic Data**		S6
	**2.1.**	**Preparation of $(C_{10}H_6)(Ph_2P)(Ph_2PF_2)$ (1)**	S6
	**2.2.**	**Preparation of $(C_{10}H_6)(Ph_2PF_2)_2$ (2)**	S8
	**2.3.**	**Preparation of $[(C_{10}H_6)(Ph_2PF)(Ph_2P)][B(C_6F_5)_4]$ (3)**	S9
	**2.4.**	**Preparation of $[(C_{10}H_6)(Ph_2PF)(Ph_2PF_2)][B(C_6F_5)_4]$ (4)**	S11
	**2.5.**	**Preparation of $[(C_{10}H_6)(Ph_2PF)_2][B(C_6F_5)_4]_2$ (5)**	S13
	**2.6**	**Preparation of $(CH_2)_1(Ph_2PF_2)_2$ (8a)**	S15
	**2.7**	**Preparation of $(CH_2)_2(Ph_2PF_2)_2$ (8b)**	S17
	**2.8**	**Preparation of $(CH_2)_3(Ph_2PF_2)_2$ (8c)**	S18
	**2.9**	**Preparation of $(CH_2)_4(Ph_2PF_2)_2$ (8d)**	S20
	**2.10**	**Preparation of $(CH_2)_5(Ph_2PF_2)_2$ (8e)**	S21
	**2.11**	**Attempted stepwise oxidation of 1,1-bis(diphenylphosphino)methane**	S23
	**2.12**	**Preparation of $[(CH_2)_1(Ph_2PF)_2][B(C_6F_5)_4]_2$ (9a)**	S25
	**2.13**	**Preparation of $[(CH_2)_2(Ph_2PF)_2][B(C_6F_5)_4]_2$ (9b)**	S27
	**2.14**	**Preparation of $[(CH_2)_3(Ph_2PF)_2][B(C_6F_5)_4]_2$ (9c)**	S29
	**2.15**	**Preparation of $[(CH_2)_4(Ph_2PF)_2][B(C_6F_5)_4]_2$ (9d)**	S31
	**2.16**	**Preparation of $[(CH_2)_5(Ph_2PF)_2][B(C_6F_5)_4]_2$ (9e)**	S33
	**2.17**	**$^{31}P\{^1H\}$ and $^{19}F\{^1H\}$ NMR parameters of 8a-e and 9a-e**	S35
	**2.18**	**Preparation of Ph_3PF_2**	S36
	**2.19**	**Preparation of $[Ph_3PF][B(C_6F_5)_4]$**	S36
	**2.20**	**Reaction of 5, $[Ph_3PF][B(C_6F_5)_4]$ and 9a-e with Et_3PO (Gutmann-Becket test)**	S37
	**2.21**	**Friedel-Crafts dimerization of 1,1-diphenylethylene with 5, $[Ph_3PF][B(C_6F_5)_4]$ and 9a-e as catalysts**	S43
	**2.22**	**Dehydrocoupling of Et_3SiH and Phenol with 5, $[Ph_3PF][B(C_6F_5)_4]$ and 9a-e as catalysts**	S45
	**2.23**	**Hydrosilylation of 1,1-diphenylethylene with Et_3SiH**	S47

using 5, $[Ph_3PF][B(C_6F_5)_4]$ and 9a-e as catalysts

2.24 Deoxygenation of benzophenone in the presence of Et_3SiH using 5, $[Ph_3PF][B(C_6F_5)_4]$ and 9a-e as catalysts S49

2.25 Hydrodefluorination of fluoropentane in the presence of Et_3SiH using 5, $[Ph_3PF][B(C_6F_5)_4]$ and 9a-e as catalysts S51

3. Crystallographic Details S53

4. References S55

1. Materials and Methods

General Remarks

All manipulations were performed in a glove box MB Unilab produced by MBraun or using standard Schlenk techniques[S1] under an inert atmosphere of anhydrous N_2. Dry, oxygen-free solvents (CH_2Cl_2, *n*-pentane, toluene) were prepared using an Innovative Technologies solvent purification system. Fluorobenzene (C_6H_5F) was distilled from CaH_2 and stored over molecular sieves (4 Å) prior to use. Deuterated dichloromethane (CD_2Cl_2) and bromobenzene (C_6D_5Br) were purchased from Sigma-Aldrich, distilled from CaH_2 and stored over molecular sieves (4 Å) for at least two days prior to use. Reagents such as 1,8-Bis(diphenylphosphino)naphthalene, 1,1-Bis(diphenylphosphino)methane, 1,2-Bis(diphenylphosphino)ethane, 1,3 Bis(diphenylphosphino) propane, 1,4-Bis(diphenylphosphino)butane, 1,5-Bis(diphenylphosphino)pentane, XeF_2, Et_3PO, Et_3SiH, Ph_3P, 1-fluoropentane, 1,1-diphenylethylene, phenol and benzophenone were purchased either from Sigma-Aldrich, Strem Chemicals or Alfa Aesar and, if applicable, distilled prior to use. Compound $[Et_3Si][B(C_6F_5)_4]*2(C_7H_8)$ was prepared according to a literature known procedure.[S2] All glassware was oven-dried at temperatures above 180°C prior to use. NMR spectra were measured on a Bruker AVANCE 400 (^1H: 400 MHz, ^{11}B: 128 MHz, ^{13}C: 101 MHz, ^{31}P: 162 MHz, ^{19}F: 377 MHz) or Agilent DD2 500 (^1H: 500 MHz, ^{13}C: 125 MHz, ^{31}P: 202 MHz, ^{19}F: 471 MHz) at ambient temperature. All ^{13}C NMR spectra were exclusively recorded with composite pulse decoupling. Assignments of the carbon atoms in the ^{13}C spectra were performed via indirect deduction from the cross-peaks in 2D correlation experiments (HMBC; HSQC). Chemical shifts were referenced to $\delta_{TMS} = 0.00$ ppm (^1H, ^{13}C), $\delta_{BF3*OEt2} = 0.00$ ppm (^{11}B), $\delta_{CFCl3} = 0.00$ ppm (^{19}F) and $\delta_{H3PO4(85\%)} = 0.00$ ppm (^{31}P, externally). Chemical shifts (δ) are reported in ppm, multiplicity is reported as follows (s = singlet, d = doublet, t = triplet, m = multiplet) and coupling constants (J) are reported in Hz. Assignments of individual resonances were done using 2D techniques (HMBC, HSQC, HH-COSY) when necessary. Yields of products in solution were determined by integration of all resonances observed in the respective NMR spectra if not stated otherwise. High-resolution mass spectra (HRMS) were obtained on a micro mass 70S-250 spectrometer (EI), an ABI/Sciex QStar Mass Spectrometer (DART), or on a JOEL AccuTOF-DART (DART). Elemental analyses (C, H, N) were performed at the University of Toronto employing a Perkin Elmer 2400 Series II CHNS Analyzer.

X-ray Diffraction Studies.

Single crystals were coated with Paratone-N oil, mounted using a glass fibre pin and frozen in the cold nitrogen stream of the goniometer. Data sets were collected on a Siemens Smart System CCD diffractometer which was equipped with a rotation anode using graphite-monochromated MoKα radiation (λ = 0.71073 Å) Data reduction was performed using the Bruker SMART[S3] software package. Data sets were corrected for absorption effects using SADABS routine (empirical multi-scan method). Structure solutions were found with the SHELXS-97 package using the direct method and were refined with SHELXL-97[S4] against F^2 using first isotropic and anisotropic thermal parameters for all non-hydrogen atoms. The unit cell of **4** contains 5 molecules CH_2Cl_2 which have been treated as a diffuse contribution to the overall scattering without specific atom positions by SQUEEZE/PLATON due to their high degree of disorder. Hydrogen atoms bonded to carbon atoms were generated with idealized geometries and isotropically refined using a riding model. Further details are given in tables S3.1 and S3.2 (pages S54-S55).

2. Syntheses and Spectroscopic Data

2.1. Preparation of $(C_{10}H_6)(Ph_2PF_2)(Ph_2P)$ (1)

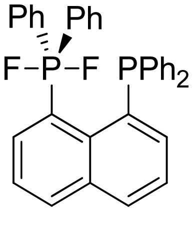

1

XeF_2 (76 mg, 0.45 mmol, 0.9 eq.) was added portionwise to a solution of 1,8-bis(diphenylphosphino)naphthalene (248 mg, 0.50 mmol, 1.0 eq.) in CH_2Cl_2 (15 mL) at −35 °C. The solution was slowly warmed to ambient temperature and stirred for one hour. All volatiles were removed *in vacuo* and the remaining yellowish solid was washed with *n*-pentane (3 x 3 mL) yielding **1** as a colourless material (208 mg, 87% yield).

^{1}H NMR (CD_2Cl_2, [ppm]): δ = 6.94 - 7.00 (4H, m, Ph), 7.16 - 7.24 (8H, m, Ph), 7.26 - 7.37 (8H, m, Ph), 7.44 - 7.49 (1H, m, naphthyl), 7.51 - 7.55 (1H, m, naphthyl), 7.78 - 7.82 (1H, m, naphthyl), 7.85 - 7.91 (2H, m, naphthyl), 7.91 - 7.96 (1H, m, naphthyl); **^{13}C{^{1}H} NMR (CD_2Cl_2, [ppm]):** δ = 124.8 (1C, d, *p*-naphthyl, $^4J_{CP}$ = 23.5 Hz), 125.9 (1C, d, *p*-naphthyl, $^4J_{CP}$ = 6.1 Hz), 128.3 (4C, s(br), Ph), 128.5 (4C, m, Ph), 130.4 (4C, s(br), Ph), 131.5 (1C, m, naphthyl), 131.8 (1C, m, naphthyl), 133.2 (4C, d, Ph, $^nJ_{CP}$ = 18.0 Hz), 134.0 (1C, t, naphthyl, $^nJ_{CF}$ = 10.5 Hz), 134.2 (4C, s(br), Ph), 135.1 (1C, m, naphthyl), 135.5 (1C, m, naphthyl), 137.1 (1C, t, naphthyl, $^nJ_{CF}$ = 1.8 Hz), 137.7 (2C, d, *i*-Ph, $^1J_{CP}$ = 12.3 Hz), 139.4 (2C, d, *i*-Ph, $^1J_{CP}$ = 212.4 Hz), resonances for the quaternary carbon atoms of the naphthyl-moiety were not observed; **^{19}F{^{1}H} NMR (CD_2Cl_2, [ppm]):** δ = −42.0 (2F, dd, $^1J_{FP}$ = 742 Hz, $^5J_{FP}$ = 17 Hz); **^{31}P{^{1}H} NMR (CD_2Cl_2, [ppm]):** δ = −55.5 (1P, td, $^1J_{PF}$ = 742 Hz, $^4J_{PP}$ = 10 Hz), −18.5 (1P, $^5J_{PF}$ = 17 Hz, td, $^4J_{PP}$ = 10 Hz); **elemental analysis** for $C_{34}H_{26}F_2P_2$: calcd.: C 76.4, H 4.9, found: C 76.3, H 5.3; **ESI MS**: m/z: 515.1479 (calcd. for $M{-}F^+$: 515.1488).

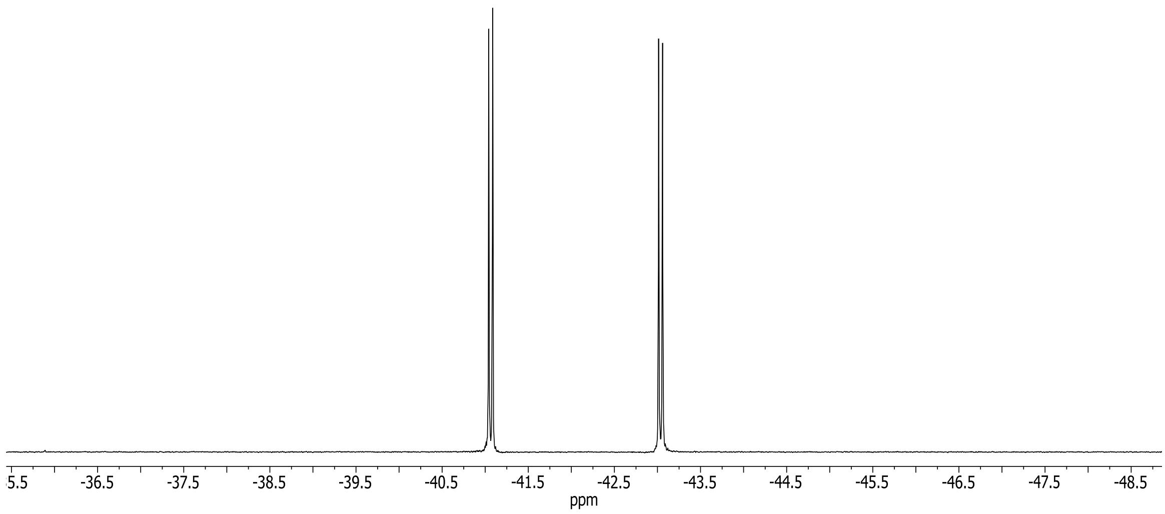

$^{19}F\{^1H\}$ NMR spectrum of compound **1** (CD_2Cl_2).

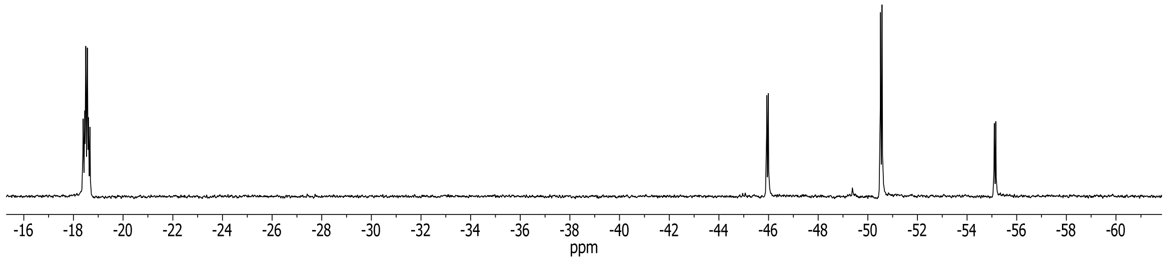

$^{31}P\{^1H\}$ NMR spectrum of compound **1** (CD_2Cl_2).

2.2. Preparation of $(C_{10}H_6)(Ph_2PF_2)_2$ (2)

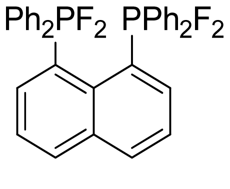

2

XeF_2 (406 mg, 2.4 mmol, 2.2 eq.) was added portionwise to a yellow solution of 1,8-bis(diphenylphosphino)naphthalene (496 mg, 1.00 mmol, 1.0 eq.) in CH_2Cl_2 (10 mL). The reaction mixture was stirred at ambient temperature for six hours giving a colourless solution. All volatiles were removed *in vacuo* yielding **2** as a colourless solid (509 mg, 95% yield).

^{1}H NMR (CD_2Cl_2, [ppm]): δ = 7.05 - 7.13 (8H, m, *m*-Ph), 7.21 - 7.27 (4H, m, *p*-Ph), 7.33 - 7.42 (8H, m, *o*-Ph), 7.44 - 7.50 (2H, m, *p*-naphthyl), 7.94 - 8.01 (2H, m, *m*-naphthyl), 8.46 - 8.57 (2H, m, *o*-naphthyl); **^{13}C{^{1}H} NMR (CD_2Cl_2, [ppm]):** δ = 125.0 (2C, d, *p*-naphthyl, $^4J_{CP}$ = 20.9 Hz), 128.1 (8C, d, *m*-Ph, $^3J_{CP}$ = 17.2 Hz), 130.3 (4C, d, *p*-Ph, $^4J_{CP}$ = 3.5 Hz), 133.3 (8C, dt, *o*-Ph, $^2J_{CP}$ = 12.8 Hz, $^3J_{CF}$ = 8.1 Hz), 133.8 (2C, m, *m*-$C_{naphthyl}$H), 137.4 (2C, dtdt, *i*-naphthyl, $^1J_{CP}$ = 202.4 Hz, $^2J_{CF}$ = 31.6 Hz, $^3J_{CP}$ = 3.6 Hz, $^4J_{CF}$ = 3.6 Hz), 138.5 (2C, dt, *o*-$C_{naphtyl}$H, $^2J_{CP}$ = 14.5 Hz, $^3J_{CF}$ = 14.5 Hz), 139.8 (4C, dt, *i*-Ph, $^1J_{CP}$ = 182.3 Hz, $^2J_{PF}$ = 28.4 Hz), resonances for the quaternary carbon atoms of the naphthyl-moiety were not observed; **^{19}F{^{1}H} NMR (CD_2Cl_2, [ppm]):** δ = −34.3 (4F, X_2X_2' part of AA'X_2X_2' spin system, $^1J_{AX}$ = $^1J_{A'X'}$ = 717 Hz, $^5J_{A'X}$ = $^5J_{AX'}$ = −18 Hz, $^6J_{XX'}$ = 0 Hz); **^{31}P{^{1}H} NMR (CD_2Cl_2, [ppm]):** δ = −49.4 (2P, AA' part of AA'X_2X_2' spin system, $^5J_{AA'}$ = 3 Hz); **elemental analysis** for $C_{34}H_{26}F_4P_2$: calcd.: C 71.2, H 4.6, found: C 71.0, H 5.0; **ESI MS**: m/z: 553.1 (calcd. for M−F^+: 553.1).

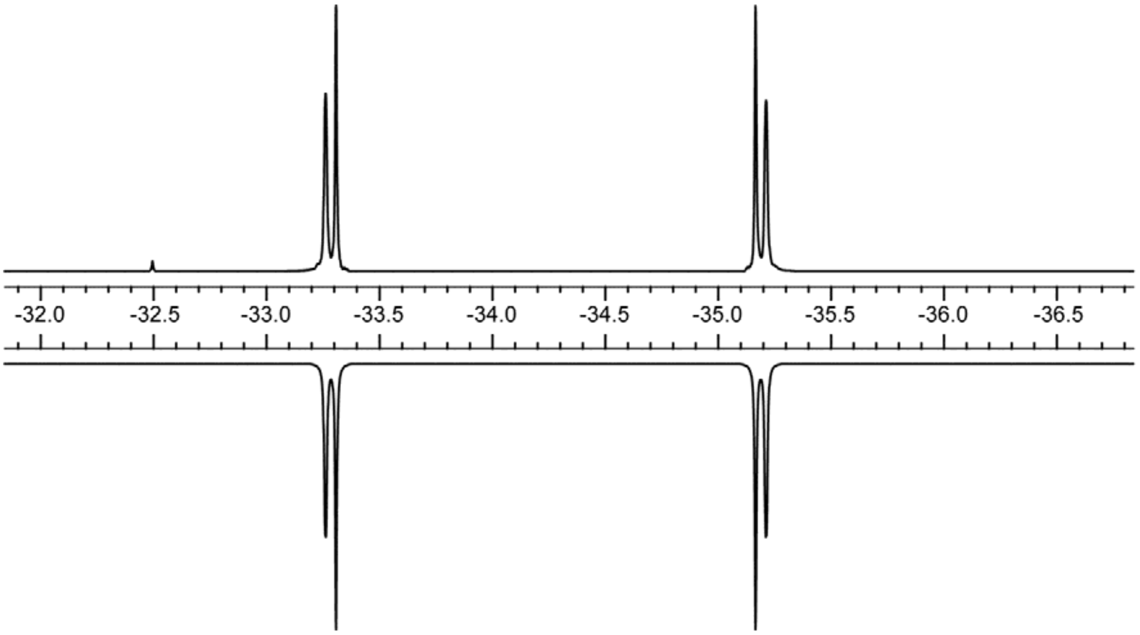

^{19}F{^{1}H} NMR spectrum of compound **2** (CD_2Cl_2, upwards) and simulated spectrum (downwards).

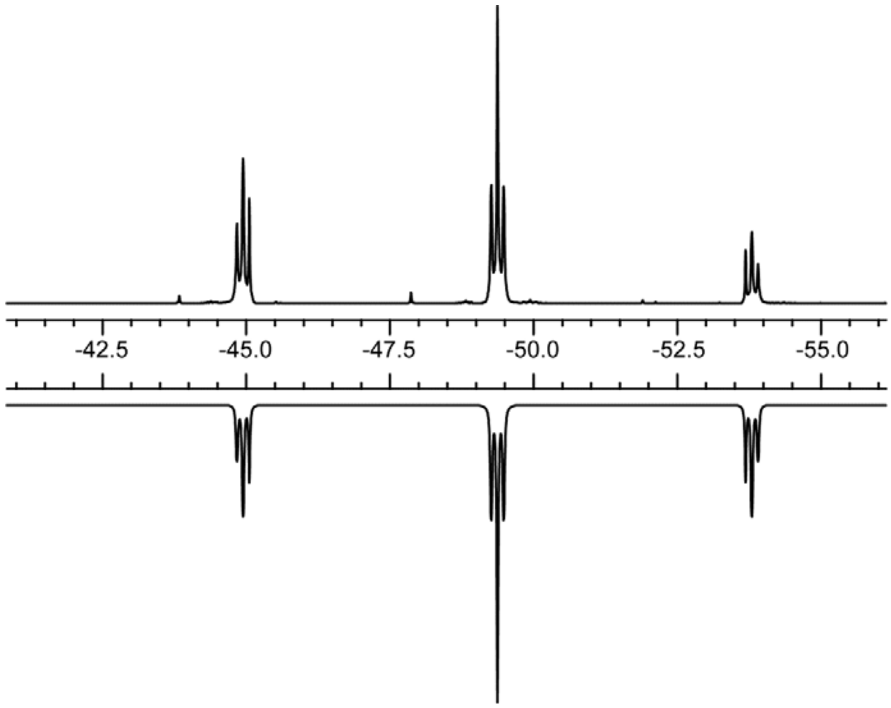

$^{31}P\{^1H\}$ NMR spectrum of compound **2** (CD_2Cl_2, upwards) and simulated spectrum (downwards).

2.3. Preparation of [($C_{10}H_6$)(Ph_2PF)(Ph_2P)][B(C_6F_5)$_4$] (3)

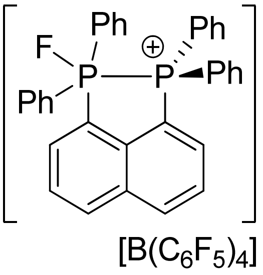

3

Freshly prepared [Et_3Si][B(C_6F_5)$_4$]*2(C_7H_8) (196 mg, 0.20 mmol, 1.0 eq.) was added portion wise to a solution of **1** (107 mg, 0.20 mmol, 1.0 eq.) in C_6H_5F (5 mL). The clear, yellowish reaction mixture was stirred for 30 min at ambient temperature. Addition of *n*-pentane (10 mL) gave a colourless precipitate. The supernatant was removed and the residue was washed with *n*-pentane (3 x 3 mL). Removal of all volatiles *in vacuo* gave **3** as colourless, microcrystalline solid (208 mg, 87% yield).

^{1}H NMR (CD_2Cl_2, [ppm]): δ = 6.67 - 6.65 (4H, m, Ph), 7.09 - 7.16 (4H, m, Ph), 7.20 - 7.28 (4H, m, Ph), 7.44 - 7.49 (8H, m, Ph), 7.82 - 7.94 (2H, m, naphthyl), 8.09 - 8.15 (1H, m, naphthyl), 8.46 - 8.51 (1H, m, naphthyl), 8.58 - 8.62 (1H, m, naphthyl), 8.98 - 9.05 (1H, m, naphthyl); **^{11}B{^{1}H} NMR (CD_2Cl_2, [ppm]):** δ = −16.6 (s); **^{13}C{^{1}H} NMR (CD_2Cl_2, [ppm]):** δ = 115.6 (1C, d, *i*-naphthyl, $^1J_{CP}$ = 20.9), 118.0 (1C, ddd, *i*-napthyl, $^1J_{CP}$ = 140.0 Hz, $^2J_{CF}$ = 40.7 Hz, $^3J_{CP}$ = 20.9 Hz), 121.1 (2C, dd, *i*-Ph, $^1J_{CP}$ = 40.5 Hz, $^4J_{CF}$ = 3.0 Hz), 125.0 (2C, ddd, *i*-Ph, ddd,

$^{1}J_{CF}$ = 38.7 Hz, $^{5}J_{CP}$ = 1.6 Hz, $^{6}J_{CF}$ = 1.6 Hz), 128.8 - 129.2 (6C, m, Ph / naphthyl), 129.7 (4C, d, Ph, J_{CP} = 10.4 Hz), 130.0 (4C, dd, Ph, J_{CP} = 17.1 Hz, J_{CF} = 1.7 Hz), 131.9 (2C, d, Ph, J_{CP} = 3.1 Hz), 132.4 (4C, dd, Ph, J_{CP} = 9.1 Hz, J_{CP} = 0.9 Hz), 132.7 (2C, m, Ph), 134.7 (1C, ddd, naphthyl, J_{CP} = 11.7 Hz, J_{CF} = 9.9 Hz, J_{CP} = 2.2 Hz), 136.7 (8C, d(br), C_6F_5, $^{1}J_{CF}$ = 246 Hz), 137.6 - 137.9 (2C, m, naphthyl), 138.6 (4C, d(br), C_6F_5, $^{1}J_{CF}$ = 237 Hz), 143.3 (1C, ddd, naphthyl, J_{CP} = 16.7 Hz, $J_{CF/P}$ = 13.5 Hz, $J_{CF/P}$ = 10.6 Hz), 148.5 (8C, d(br), C_6F_5, $^{1}J_{CF}$ = 241 Hz), resonances for the quaternary carbon atoms of the naphthyl-moiety were not observed; **^{19}F{^{1}H} NMR (CD_2Cl_2, [ppm]):** $\delta = -167.6$ (8F, m, *m*-C_6F_5), −163.7 (4F, t, *p*-C_6F_5, $^{3}J_{FF}$ = 20 Hz), −133.1 (8F, m, *o*-C_6F_5), −36.5 (1F, dd, PF, $^{1}J_{FP}$ = 783 Hz, $^{2}J_{FP}$ = 164 Hz); **^{31}P{^{1}H} NMR (CD_2Cl_2, [ppm]):** $\delta = -17.4$ (1P, dd, PPh_2F, $^{1}J_{PF}$ = 783 Hz, $^{1}J_{PP}$ = 138 Hz), −5.5 (1P, dd, PPh_2, $^{2}J_{PF}$ = 164 Hz, $^{1}J_{PP}$ = 138 Hz); **elemental analysis** for $C_{58}H_{26}F_{21}BP_2$: calcd.: C 58.3, H 2.2, found: C 57.7, H 2.3; **ESI MS**: m/z: 515.1487 (calcd. for M^+: 515.1488).

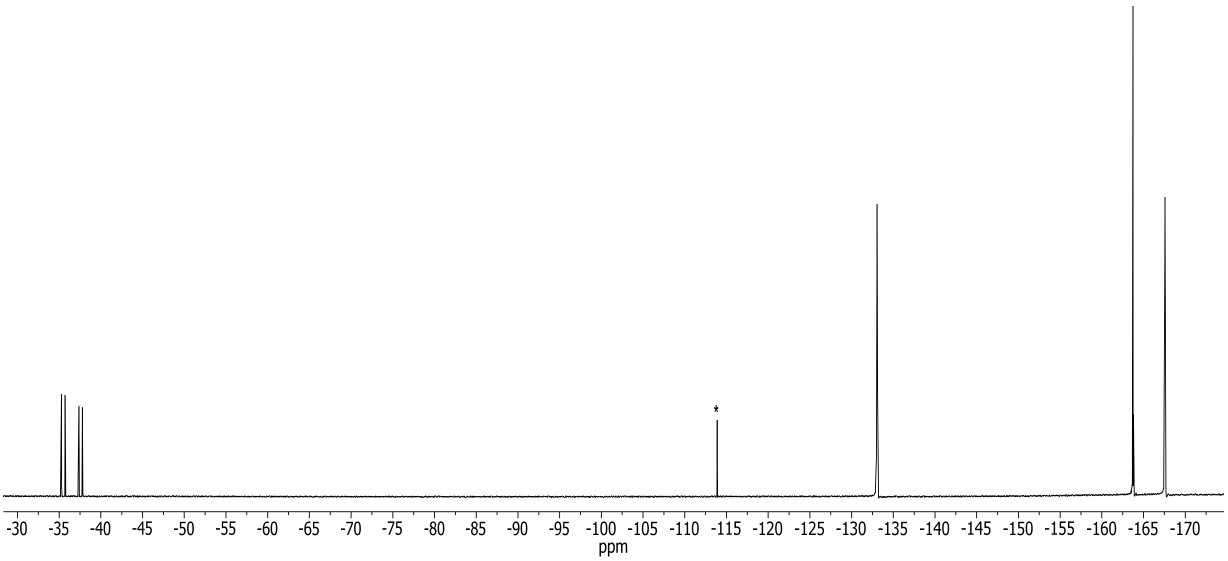

^{19}F{^{1}H} NMR spectrum of compound **3** (CD_2Cl_2), * indicates traces of C_6H_5F.

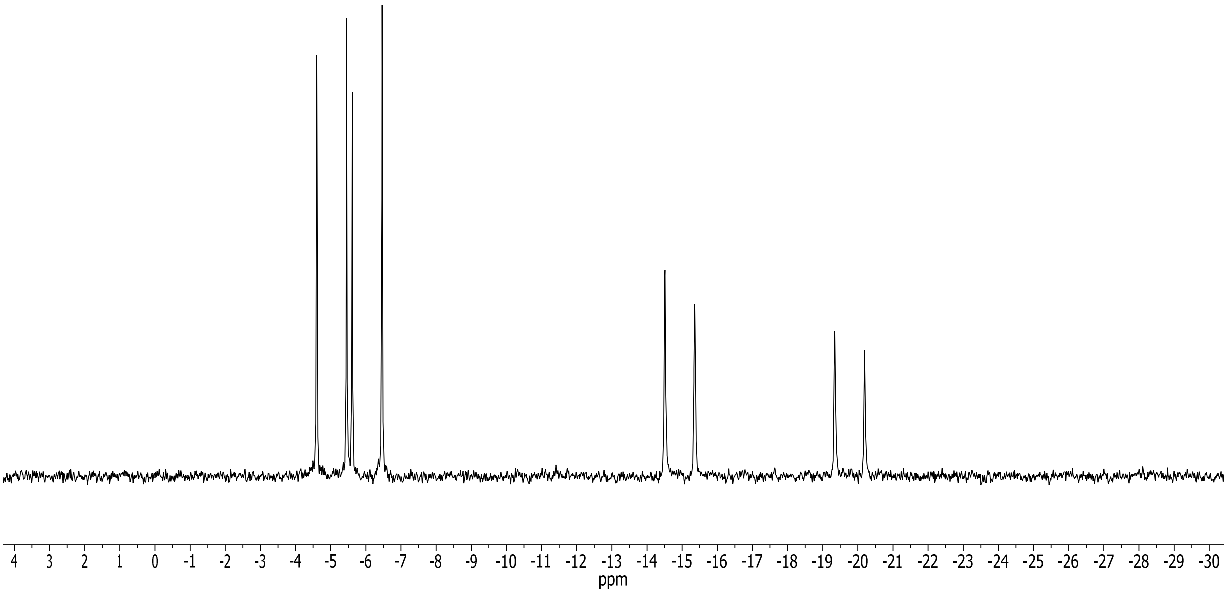

$^{31}P\{^1H\}$ NMR spectrum of compound **3** (CD_2Cl_2).

2.4. Preparation of $[(C_{10}H_6)(Ph_2PF)(Ph_2PF_2)][B(C_6F_5)_4]$ (4)

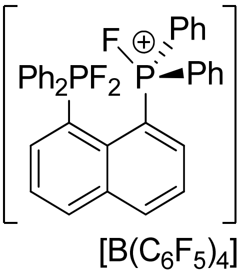

4

Freshly prepared $[Et_3Si][B(C_6F_5)_4]$*2(C_7H_8) (156 mg, 0.16 mmol, 1.0 eq.) was added portionwise to a solution of **2** (85 mg, 0.16 mmol, 1.0 eq.) in C_6H_5F (5 mL). The clear, yellowish solution was stirred for 30 min at ambient temperature. Addition of *n*-pentane (10 mL) gave a colourless precipitate. The supernatant was removed and the residue was washed with *n*-pentane (3 x 3 mL). Removal of all volatiles *in vacuo* gave **4** as colourless, microcrystalline solid (189 mg, 96% yield). Single crystals of **4**, as its dichloromethane solvate **4***(CH_2Cl_2), were obtained by slow diffusion of *n*-pentane into a CH_2Cl_2 solution at −35 °C and were suitable of X-ray single crystal structure determination. Portions of the crystalline material were isolated by decanting the supernatant and either removing remaining volatiles *in vacuo* or by evaporating in one atmosphere of N_2. Both samples were investigated by ^1H NMR spectroscopy. While 0.7 equivalent CH_2Cl_2 solvate were observed in the ^1H NMR spectrum of the sample that was dried *in vacuo*, the latter indicated the presence of one CH_2Cl_2 solvate molecule. This indicates that the solvate molecules are only weakly bound.

^{1}H NMR (CD_2Cl_2, [ppm]): δ = 7.12 - 7.27 (4H, m(br), Ph), 7.32 - 7.48 (12H, m(br), Ph), 7.52 - 7.68 (4H, m(br), Ph), 7.68 - 7.80 (2H, m(br), naphthyl), 7.82 - 7.92 (1H, m(br), naphthyl), 8.20 - 8.31 (1H, m(br), naphthyl), 8.36 - 8.47 (1H, m(br), naphthyl), 8.78 - 8.90 (2H, m(br), naphthyl); **^{11}B{^{1}H} NMR (CD_2Cl_2, [ppm]):** δ = −16.7 (s); **^{13}C{^{1}H} NMR (CD_2Cl_2, [ppm]):** δ = 116.0 (s(br)), 119.1 (s(br)), 119.9 (s(br)), 123.5 (s(br)), 127.0 (s(br)), 127.9 (s(br)), 132.8 (s(br)), 134.7 (s(br)), 136.1 (8C, d(br), C_6F_5, $^{1}J_{CF}$ = 244 Hz), 136.6 (s(br)), 137.8 (4C, d(br), C_6F_5, $^{1}J_{CF}$ = 236 Hz), 142.5 (s(br)), 148.1 (8C, d(br), C_6F_5, $^{1}J_{CF}$ = 242 Hz); **^{19}F{^{1}H} NMR (CD_2Cl_2, [ppm]):** δ = −167.3 (8F, m, *m*-C_6F_5), −163.4 (4F, m, *p*-C_6F_5), −132.8 (8F, m, *o*-C_6F_5), −125.0 (d(br), $^{1}J_{PF}$ = 1012 Hz), −34.5 (d(br), $^{1}J_{PF}$ = 717 Hz); **^{31}P{^{1}H} NMR (CD_2Cl_2, [ppm]):** δ = −51.7 (t(br), $^{1}J_{PF}$ = 717 Hz), 96.6 (d(br), $^{1}J_{PF}$ = 1012 Hz); **elemental analysis** for $C_{82}H_{26}F_{43}B_2P_2$: calcd.: C 56.5, H 2.1, found: C 56.5, H 1.5; **ESI MS**: m/z: 529.1 (calcd. for $[M+H_2O-2HF]^{+}$: 529.1).

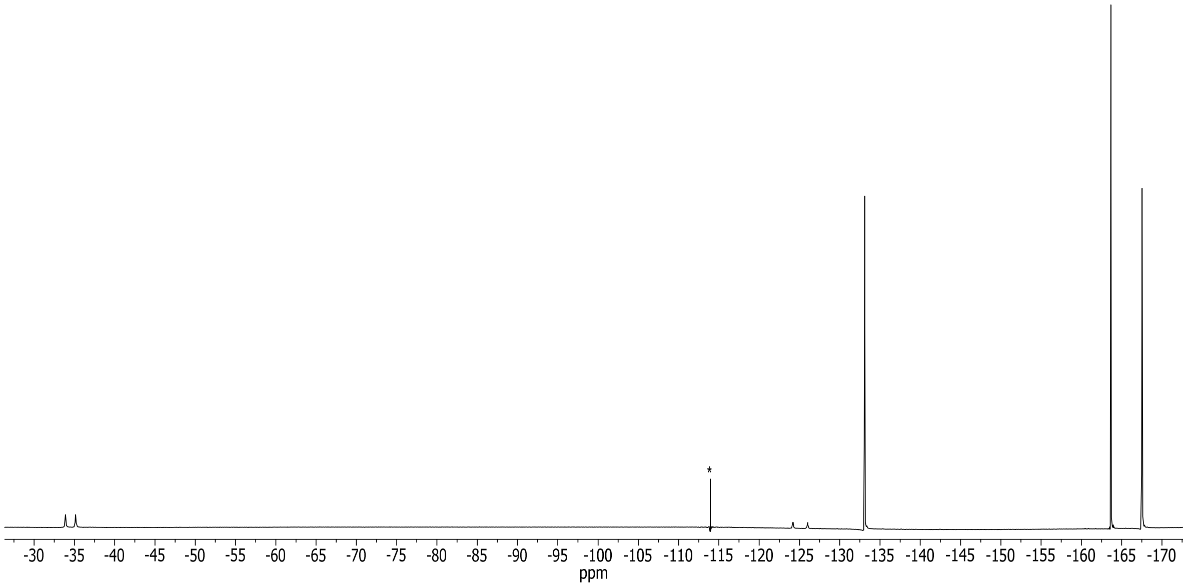

^{19}F{^{1}H} NMR spectrum of compound **4** (CD_2Cl_2), * indicates traces of C_6H_5F.

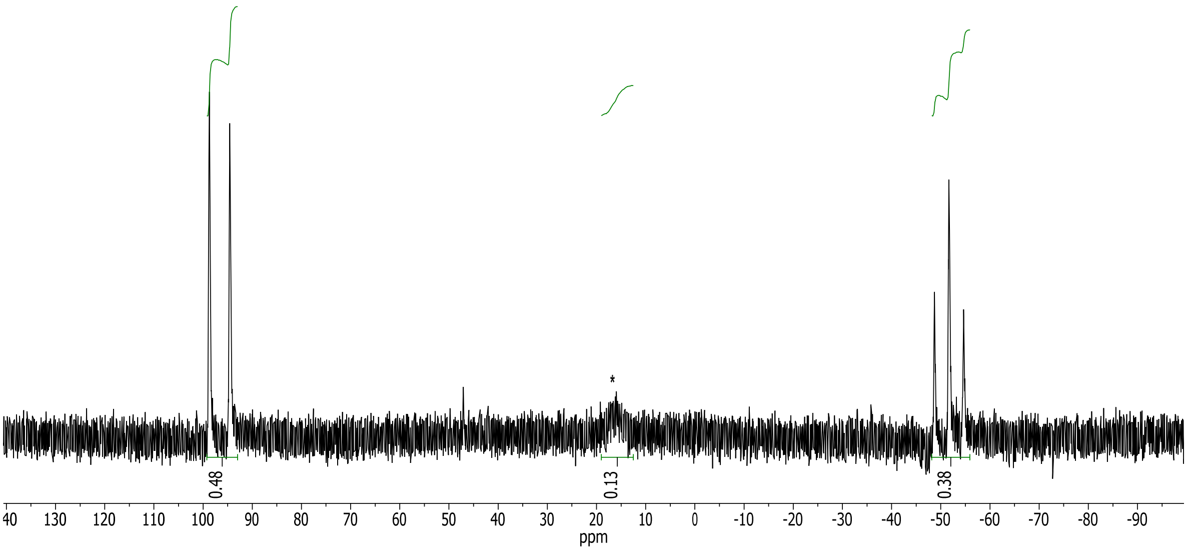

$^{31}P\{^1H\}$ NMR spectrum of compound **4** (CD_2Cl_2). *despite several tested workup procedures the broad resonance at 16 ppm persisted. Based on this, it is tentatively assigned to an isomer of **4** which is present in small amounts only. This isomer is assumed to feature a bridging P−F−P interaction caused by coordination of the σ*(P−F) acceptor orbital of the phosphonium moiety to one of the fluoro-groups of the adjacent difluorophosphorane.

2.5. Preparation of $[(C_{10}H_6)(Ph_2PF)_2][B(C_6F_5)_4]_2$ (5)

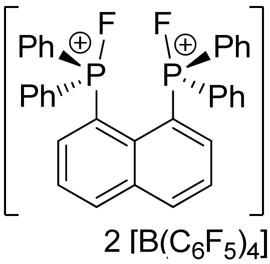

5

Freshly prepared $[Et_3Si][B(C_6F_5)_4]$*$2(C_7H_8)$ (1029 mg, 1.05 mmol, 2.0 eq.) was added to a solution of **2** (281 mg, 0.53 mmol, 1.0 eq.) in C_6H_5F (10 mL). Instantly the formation of a colourless oil was observed. The supernatant was removed and the residue was washed with *n*-pentane (3 x 3 mL). During this process, the oil turned into a colourless, microcrystalline solid. Removal of all volatiles *in vacuo* gave **5** was colourless, microcrystalline solid (842 mg, 84% yield).

^{1}H NMR (CD_2Cl_2, [ppm]): δ = 7.39 - 7.48 (8H, m, *o*-Ph), 7.67 - 7.76 (8H, m, *m*-Ph), 7.96 – 8.06 (6H, m, *p*-Ph/*p*-naphthyl), 8.08 - 8.18 (2H, m, *o*-naphthyl), 8.73 – 8.80 (2H, m, *m*-naphthyl); **$^{11}B\{^1H\}$ NMR (CD_2Cl_2, [ppm]):** δ = −16.7 (s); **$^{13}C\{^1H\}$ NMR (CD_2Cl_2, [ppm]):** δ = 111.1 (2C, dd, *i*-naphthyl, $^1J_{CP}$ = 106.9 Hz, $^2J_{CF}$ = 16.0 Hz), 116.9 (4C, dd, *i*-Ph, $^1J_{CP}$ = 109.8 Hz, $^2J_{CF}$ = 15.0 Hz), 123.8 (4C, s(br), C_6F_5), 127.9 (2C, d, *p*-$C_{naphthyl}$H, $^4J_{CP}$ = 17.3 Hz), 131.3 (8C, d, *m*-Ph, $^3J_{CP}$ = 14.7 Hz), 133.4 (8C, d, *o*-Ph, $^2J_{CP}$ = 13.1 Hz), 136.2 (16C, d(br), C_6F_5, $^1J_{CF}$ = 246 Hz), 138.1 (8C, d(br), C_6F_5, $^1J_{CF}$ = 243 Hz), 139.2 (4C, s, *p*-Ph), 142.5 - 142.6 (2C, m,

m-$C_{naphthyl}$H), 145.2 (2C, d(br), *o*-$C_{naphthyl}$H), 148.1 (16C, d(br), $^{1}J_{CF}$ = 242 Hz), resonances for the quaternary carbon atoms of the naphthyl-moiety were not observed; **^{19}F{^{1}H} NMR (CD_2Cl_2, [ppm]):** δ = −167.4 (16F, m, *m*-C_6F_5), −163.4 (8F, m, *p*-C_6F_5), −132.9 (16F, m, *o*-C_6F_5), −117.1 (2F, XX' part of AA'XX' spin system, $^{1}J_{AX}$ = $^{1}J_{A'X'}$ = 1004 Hz, $^{5}J_{A'X}$ = $^{5}J_{AX'}$ = −13 Hz, $^{6}J_{XX'}$ was not determined due to broad ^{19}F{^{1}H} resonance); **^{31}P{^{1}H} NMR (CD_2Cl_2, [ppm]):** δ = 96.8 (2P, AA' part of AA'XX' spin system, $^{5}J_{AA'}$ = 3 Hz); **elemental analysis** for $C_{82}H_{26}B_2F_{42}P_2$: calcd.: C 52.0, H 1.4, found: C 52.8, H 1.7; **ESI MS**: m/z: 529.1469 (calcd. for $[M{-}2F{+}H_2O]^{+}$: 529.1469), 543.1626 (calcd. for $[M{+}e^{-}]^{+}$: 543.1478).

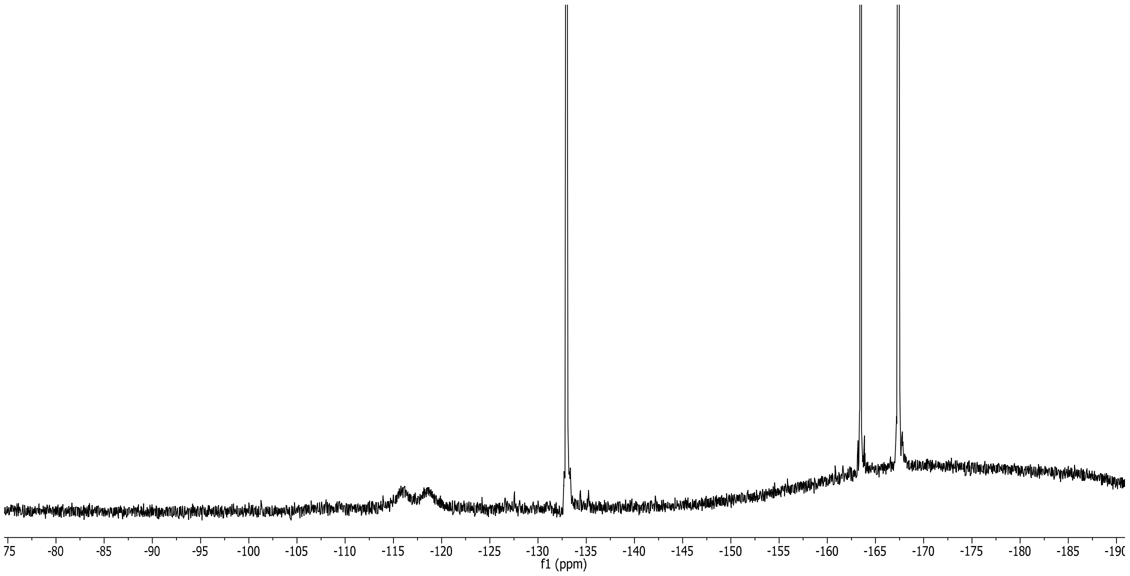

^{19}F{^{1}H} NMR spectrum of compound **5** (CD_2Cl_2).

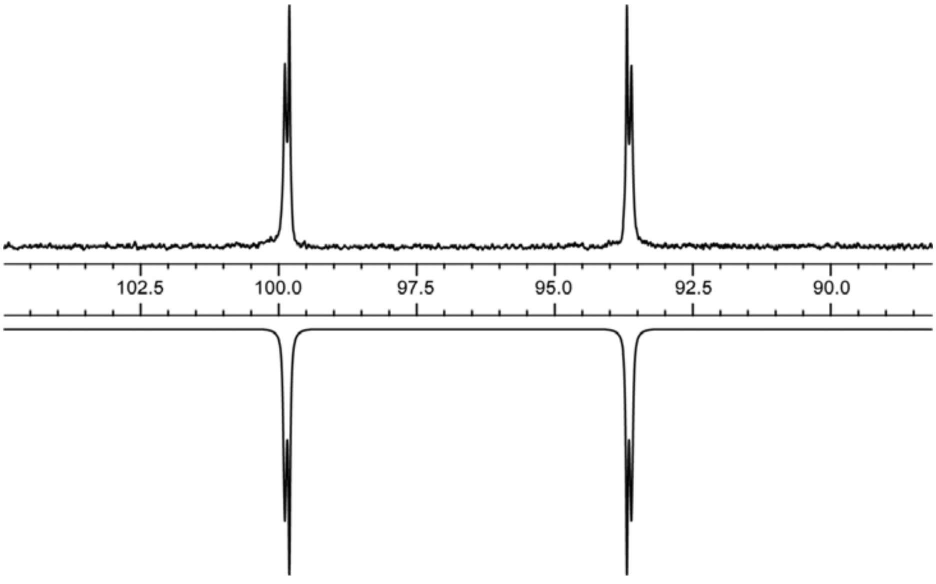

^{31}P{^{1}H} NMR spectrum of compound **5** (CD_2Cl_2, upwards) and simulated spectrum (downwards).

2.6. Preparation of $(CH_2)_1(Ph_2PF_2)_2$ (8a)[S5]

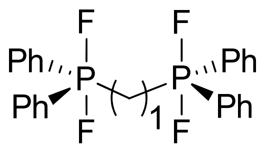

8a

A solution of XeF_2 (480 mg, 2.84 mmol, 2.2 eq.) in CH_2Cl_2 (5 mL) was added dropwise to a solution of 1,1-bis(diphenylphosphino)methane (503 mg, 1.31 mmol, 1.0 eq.) in CH_2Cl_2 (5 mL). The reaction mixture was stirred at ambient temperature for 15 min. giving a colourless solution. All volatiles were removed *in vacuo* yielding a white solid (579 mg, 96% yield). Single crystals of **8a**, suitable for X-ray single crystal structure determination were obtained by slow diffusion of *n*-pentane into a CH_2Cl_2 solution. Multi-nuclear magnetic resonance experiments were in accordance to literature reported values.[S5]

^{1}H NMR (CD_2Cl_2, [ppm]): δ = 3.57 (2H, m, CH_2), 7.37 (8H, m, *m*-Ph), 7.46 (4H, m, *p*-Ph), 7.88 (8H, m, *o*-Ph);**^{13}C{^{1}H} NMR (CD_2Cl_2, [ppm]):** δ = 46.3 (1C, td, CH_2, $^1J_{CP}$ = 132 Hz, $^2J_{CF}$ = 35 Hz), 128.6 (8C, dm, *m*-Ph, $^3J_{CP}$ = 16 Hz), 131.9 (4C, d, *p*-Ph, $^4J_{CP}$ = 5 Hz), 134.9 (8C, m, *o*-Ph), 136.1 (4C, dt, *i*-Ph, $^1J_{PF}$ = 232 Hz, $^2J_{CF}$ = 32 Hz); **^{19}F{^{1}H} NMR (CD_2Cl_2, [ppm]):** δ = –27.7 (4F, X_2X_2' part of AA'X_2X_2' spin system, $^1J_{AX}$ = $^1J_{A'X'}$ = 644 Hz, $^3J_{A'X}$ = $^3J_{AX'}$ = –18 Hz, $^4J_{XX'}$ = –7 Hz); **^{31}P{1H} NMR (CD_2Cl_2, [ppm]):** δ = –46.8 (2P, AA' part of AA'X_2X_2' spin system, $^2J_{AA'}$ = 26 Hz); **elemental analysis** for $C_{25}H_{22}F_4P_2$: calcd.: C 65.2, H 4.8, found: C 64.9, H 5.0; **DART MS**: m/z: 441.1 (calcd. for $M{-}F^+$: 441.1).

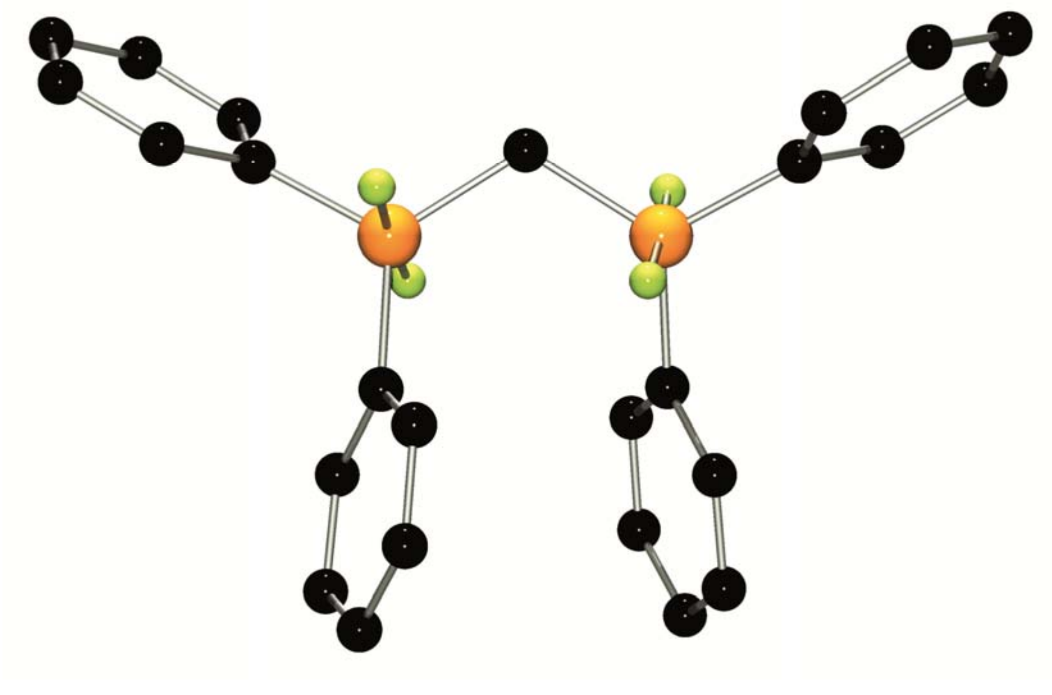

Figure 2.6.1. POV-ray depiction of **8a**. P: orange, F: yellow-green, C: black.

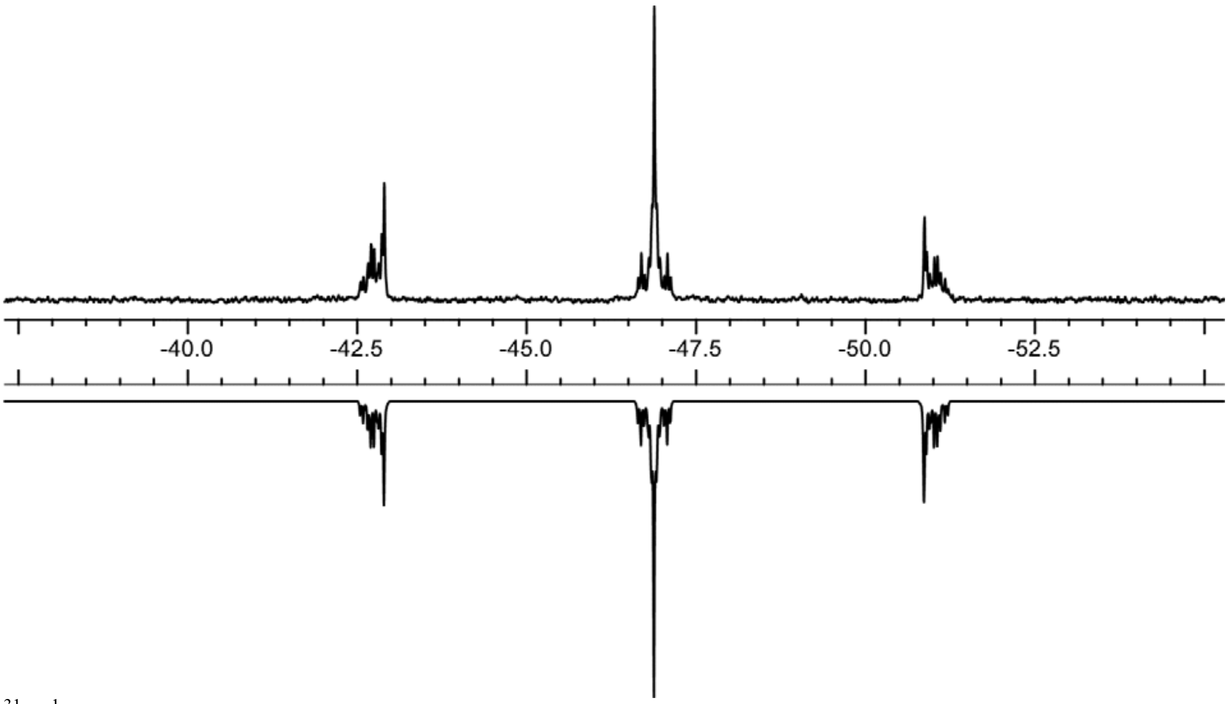

$^{31}P\{^{1}H\}$ NMR spectrum of compound **8a** (CD_2Cl_2, upwards) and simulated spectrum (downwards).

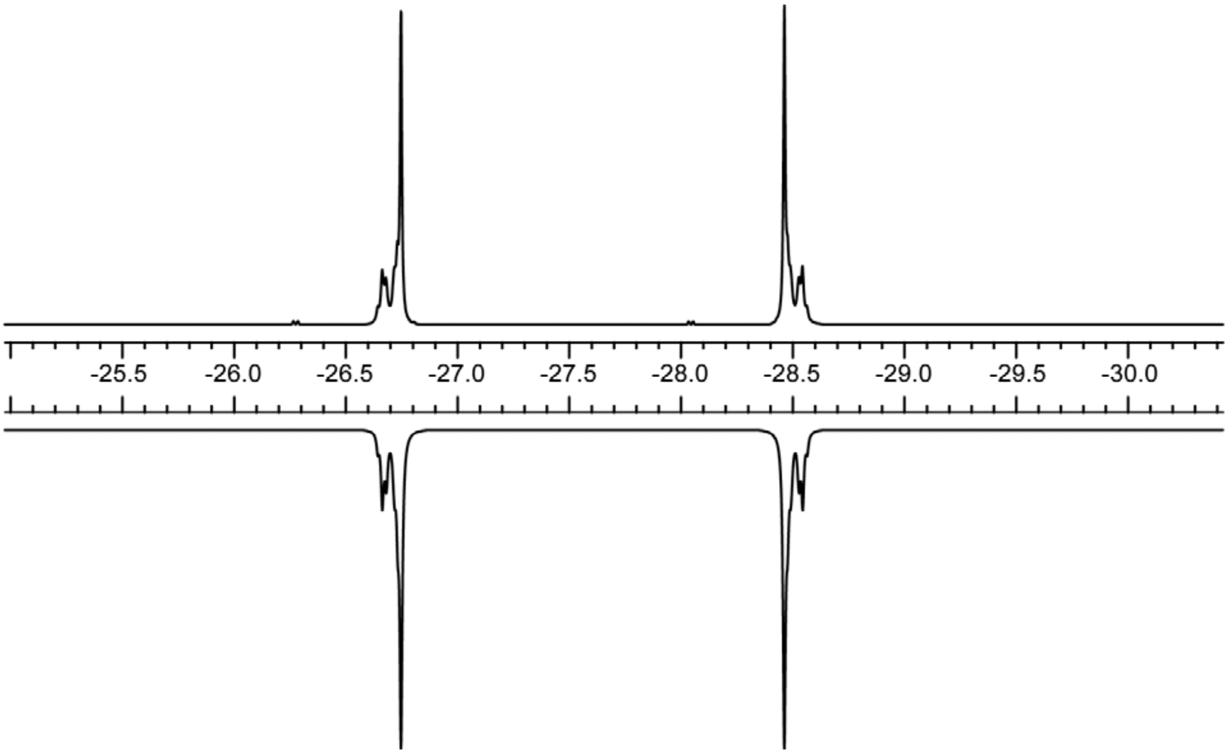

$^{19}F\{^{1}H\}$ NMR spectrum of compound **8a** (CD_2Cl_2, upwards) and simulated spectrum (downwards).

2.7. Preparation of $(CH_2)_2(Ph_2PF_2)_2$ (8b)[S5]

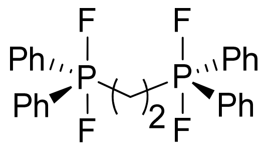

8b

A solution of XeF_2 (103 mg, 0.61 mmol, 2.4 eq.) in CH_2Cl_2 (5 mL) was added dropwise to a solution of 1,2 bis(diphenylphosphino)ethane (99 mg, 0.25 mmol, 1.0 eq.) in CH_2Cl_2 (5 mL). The reaction mixture was stirred at ambient temperature for 15 min giving a colourless solution. All volatiles were removed *in vacuo* yielding a white solid (117 mg, 99% yield). Multi-nuclear magnetic resonance experiments were in accordance to literature reported values.[S5]

^{1}H NMR (CD_2Cl_2, [ppm]): $\delta = 2.74$ (4H, m, CH_2), 7.46 (8H, m, *m*-Ph), 7.52 (4H, m, *p*-Ph), 7.97 (8H, m, *o*-Ph); **^{13}C{1H} NMR (CD_2Cl_2, [ppm]):** $\delta = 31.0$ (2C, dm, CH_2, $^1J_{CP} = 126$ Hz), 128.8 (8C, dm, *m*-Ph, $^3J_{CP} = 15$ Hz), 132.0 (4C, s(br), *p*-Ph), 134.5 (8C, m, *o*-Ph), 135.8 (4C, dt, *i*-Ph, $^1J_{PF} = 172$ Hz, $^2J_{CF} = 26$ Hz); **^{19}F{^{1}H} NMR (CD_2Cl_2, [ppm]):** $\delta = -39.7$ (4F, X_2X_2' part of AA'X_2X_2' spin system, $^1J_{AX} = {}^1J_{A'X'} = 653$ Hz, $^4J_{A'X} = {}^4J_{AX'} = -6$ Hz, $^5J_{XX'} = 0$ Hz); **^{31}P{^{1}H} NMR (CD_2Cl_2, [ppm]):** $\delta = -43.6$ (2P, AA' part of AA'X_2X_2' spin system, $^3J_{AA'} = 115$ Hz); **elemental analysis** for $C_{26}H_{24}F_4P_2$: calcd.: C 65.8, H 5.1, found: C 65.5, H 5.5; **DART MS**: m/z: 455.1 (calcd. for $M{-}F^+$: 455.1).

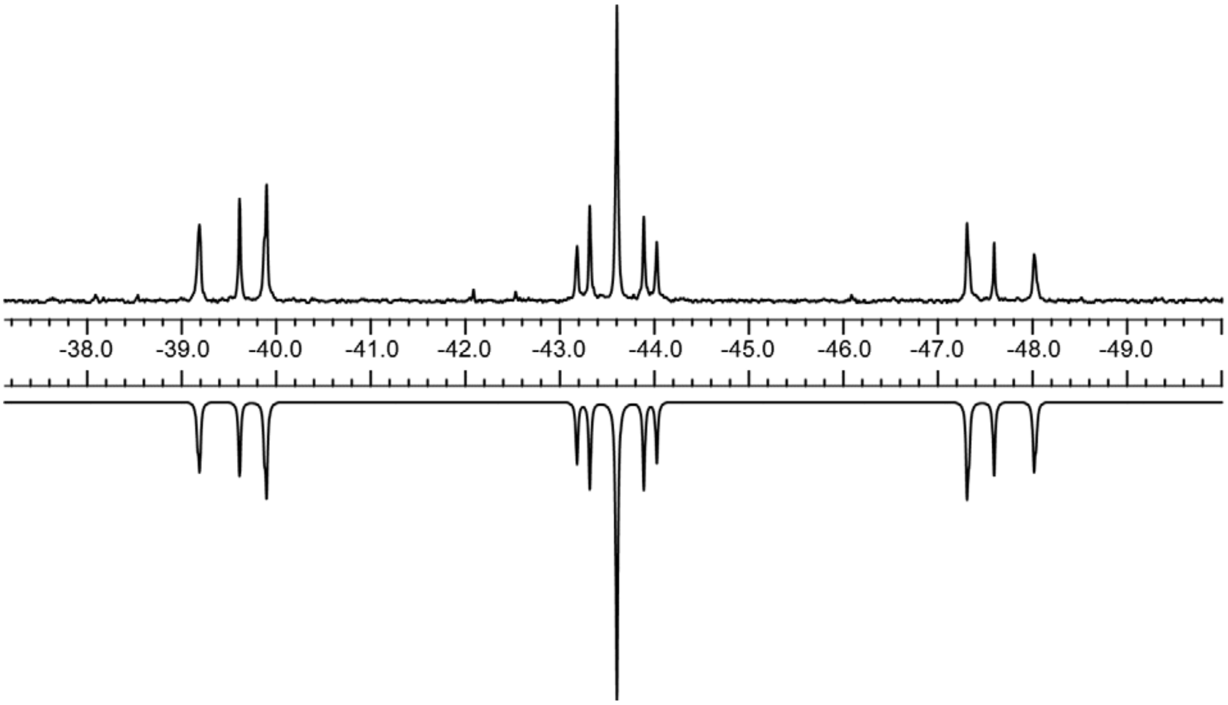

^{31}P{^{1}H} NMR spectrum of compound **8b** (CD_2Cl_2, upwards) and simulated spectrum (downwards).

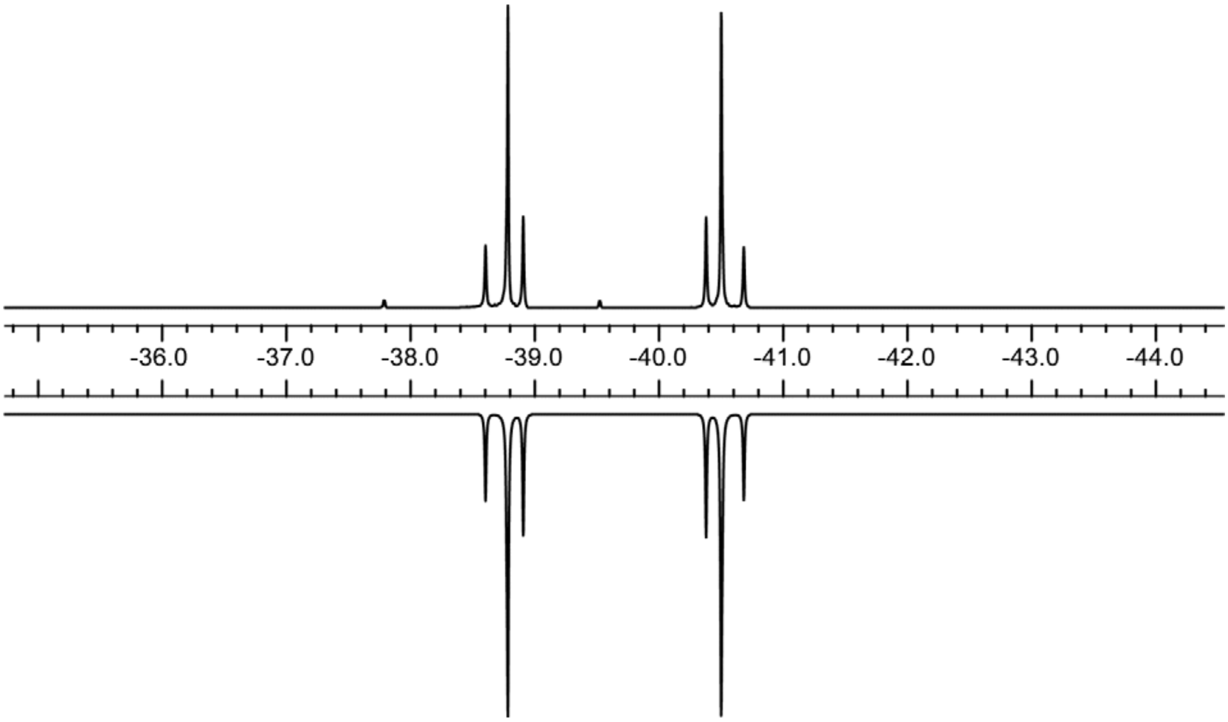

$^{19}F\{^{1}H\}$ NMR spectrum of compound **8b** (CD_2Cl_2, upwards) and simulated spectrum (downwards).

2.8. Preparation of $(CH_2)_3(Ph_2PF_2)_2$ (8c)[S6]

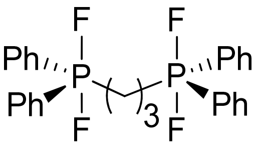

8c

A solution of XeF_2 (112 mg, 0.56 mmol, 2.1 eq.) in CH_2Cl_2 (5 mL) was added dropwise to a solution of 1,3 bis(diphenylphosphino)propane (131 mg, 0.32 mmol, 1.0 eq.) in CH_2Cl_2 (5 mL). The reaction mixture was stirred at ambient temperature for 15 min giving a colourless solution. All volatiles were removed *in vacuo* yielding a white solid (142 mg, 88% yield). Multi-nuclear magnetic resonance experiments were in accordance to literature reported values.[S6]

^{1}H NMR (CD_2Cl_2, [ppm]): δ = 2.20 (2H, m, CH_2), 2.53 (4H, m, P-CH_2), 7.45 (8H, m, *m*-Ph), 7.52 (4H, m, *p*-Ph), 7.92 (8H, m, *o*-Ph); **^{13}C {^{1}H} NMR (CD_2Cl_2, [ppm]):** δ = 19.6 (1C, m, CH_2), 37.6 (1C, dm, P-CH_2, $^{1}J_{CP}$ = 111 Hz), 128.7 (8C, dm, *m*-Ph, $^{3}J_{CP}$ = 16 Hz), 131.8 (4C,m, *p*-Ph), 134.2 (8C, m, *o*-Ph), 136.3 (4C, dt, *i*-Ph, $^{1}J_{CP}$ = 172 Hz, $^{2}J_{CF}$ = 26 Hz); **^{19}F{^{1}H} NMR (CD_2Cl_2, [ppm]):** δ = –39.0 (4F, X_2X_2' part of AA'X_2X_2' spin system, $^{1}J_{AX} = {}^{1}J_{A'X'}$ = 644 Hz, $^{5}J_{A'X} = {}^{5}J_{AX'}$ = −2 Hz, $^{6}J_{XX'}$ = 0 Hz); **^{31}P{^{1}H} NMR (CD_2Cl_2, [ppm]):** δ = −43.2 (2P, AA' part of

AA'X_2X_2' spin system, $^4J_{AA'}$ = 12 Hz); **elemental analysis** for $C_{27}H_{26}F_4P_2$: calcd.: C 66.4, H 5.4, found: C 66.3, H 5.0; **DART MS**: m/z: 469.2 (calcd. for $M{-}F^+$: 469.2).

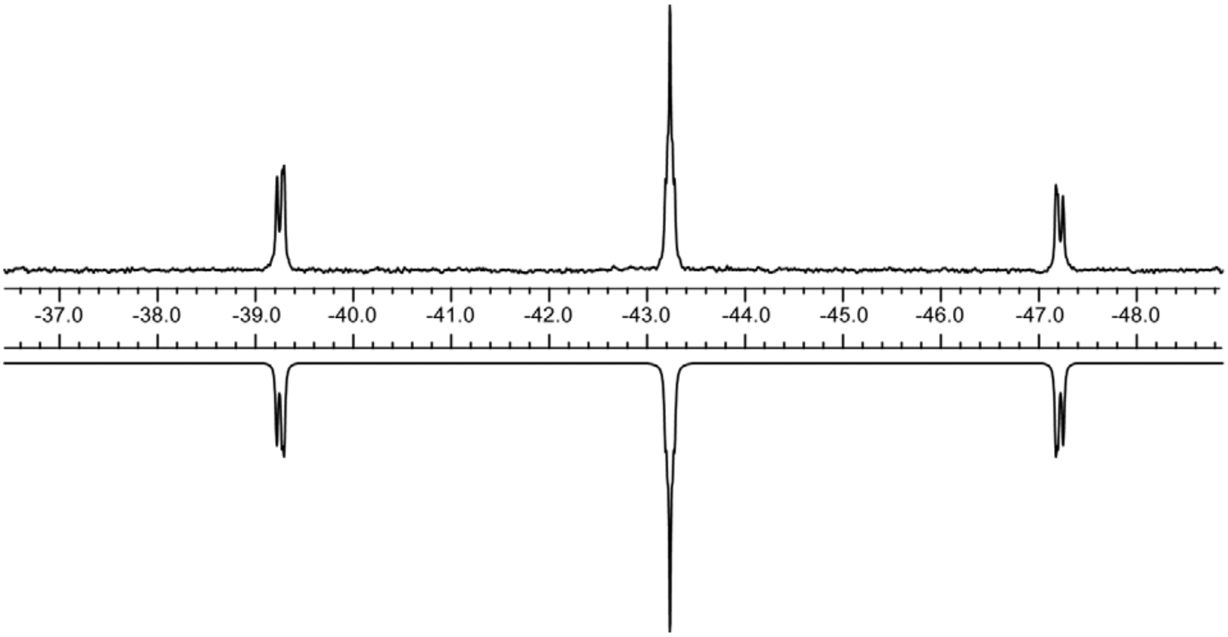

$^{31}P\{^1H\}$ NMR spectrum of compound **8c** (CD_2Cl_2, upwards) and simulated spectrum (downwards).

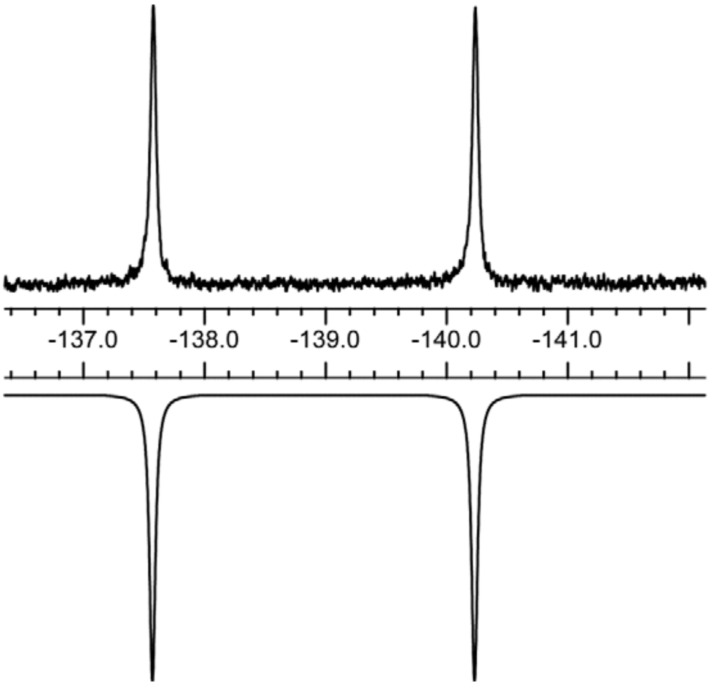

$^{19}F\{^1H\}$ NMR spectrum of compound **8c** (CD_2Cl_2, upwards) and simulated spectrum (downwards).

2.9. Preparation of $(CH_2)_4(Ph_2PF_2)_2$ (8d)[S6]

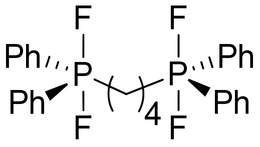

8d

A solution of XeF_2 (86 mg, 0.51 mmol, 2.1 eq.) in CH_2Cl_2 (5 mL) was added dropwise to a solution of 1,4 bis(diphenylphosphino)butane (104 mg, 0.24 mmol, 1.0 eq.) in CH_2Cl_2 (5 mL). The reaction mixture was stirred at ambient temperature for 15 min giving a colourless solution. All volatiles were removed *in vacuo* yielding a white solid (121 mg, 98% yield). Multi-nuclear magnetic resonance experiments were in accordance to literature reported values.[S6]

^{1}H NMR (CD_2Cl_2, [ppm]): δ = 1.80 (4H, m, CH_2), 2.42 (4H, m, P-CH_2), 7.44 (8H, m, *m*-Ph), 7.50 (4H, m, *p*-Ph), 7.90 (8H, m, *o*-Ph); **^{13}C{^{1}H} NMR (CD_2Cl_2, [ppm]):** δ = 25.6 (2C, m, CH_2), 36.0 (2C, dtd, P-CH_2, $^{1}J_{CP}$ = 130 Hz, $^{2}J_{CF}$ = 29 Hz, $^{4}J_{CP}$ = 1 Hz), 128.6 (8C, dt, *m*-Ph, $^{3}J_{CP}$ = 15 Hz, $^{4}J_{CF}$ = 1 Hz), 131.7 (4C, m, *p*-Ph), 134.2 (8C, m, *o*-Ph), 136.4 (4C, dt, *i*-Ph, $^{1}J_{PF}$ = 171 Hz, $^{2}J_{CF}$ = 28 Hz); **^{19}F{^{1}H} NMR (CD_2Cl_2, [ppm]):** δ = –38.9 (4F, X_2X_2' part of AA'X_2X_2' spin system, $^{1}J_{AX}$ = $^{1}J_{A'X'}$ = 641 Hz, $^{6}J_{A'X}$ = $^{6}J_{AX'}$ = 0 Hz, $^{7}J_{XX'}$ = 0 Hz); **^{31}P{^{1}H} NMR (CD_2Cl_2, [ppm]):** δ = −42.4 (2P, AA' part of AA'X_2X_2' spin system, $^{5}J_{AA'}$ = 0 Hz); **elemental analysis** for $C_{27}H_{28}F_4P_2$: calcd.: C 66.9, H 5.6, found: C 66.9, H 6.0; **DART MS**: m/z: 483.2 (calcd. for M−F^{+}: 483.2).

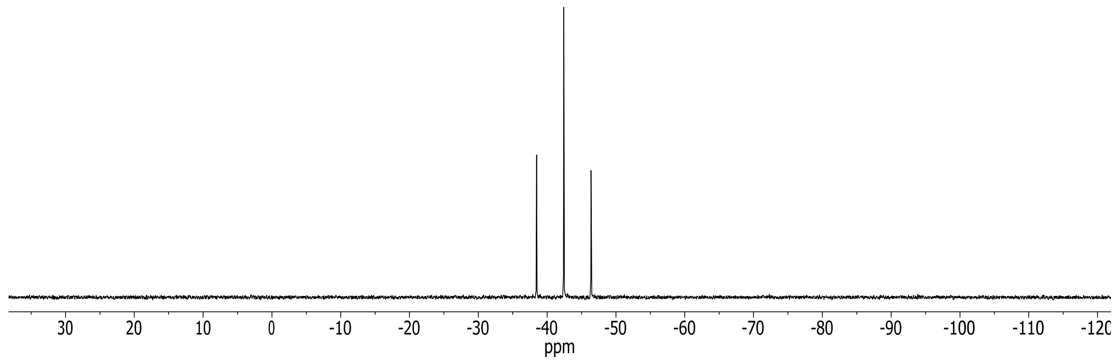

^{31}P{^{1}H} NMR spectrum of compound **8d** (CD_2Cl_2).

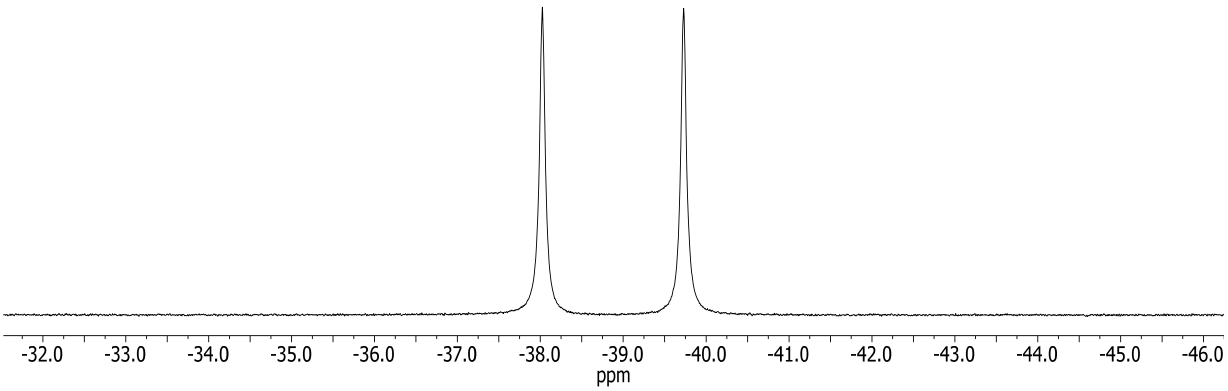

$^{19}F\{^{1}H\}$ NMR spectrum of compound **8d** (CD_2Cl_2).

2.10. Preparation of $(CH_2)_5(Ph_2PF_2)_2$ (8e)

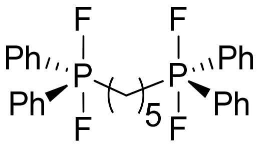

8e

A solution of XeF_2 (86 mg, 0.51 mmol, 2.2 eq.) in CH_2Cl_2 (5 mL) was added dropwise to a solution of 1,4 bis(diphenylphosphino)pentane (101 mg, 0.23 mmol, 1.0 eq.) in CH_2Cl_2 (5 mL). The reaction mixture was stirred at ambient temperature for 15 min giving a colourless solution. All volatiles were removed *in vacuo* yielding a white solid (118 mg, 99% yield).

^{1}H NMR (CD_2Cl_2, [ppm]): δ = 1.47 (2H, p, CH_2, $^{3}J_{HH}$ = 10 Hz), 1.76 (4H, m, CH_2), 2.41 (4H, s(br), P-CH_2), 7.45 (8H, m, *m*-Ph), 7.51 (4H, m, *p*-Ph), 7.95 (8H, m, *o*-Ph); **$^{13}C\{^{1}H\}$ NMR (CD_2Cl_2, [ppm]):** δ = 24.0 1C, CH_2), 32.5 (2C, t, CH_2, $^{2}J_{CP}$ = 21 Hz) 36.2 (2C, dt, P-CH_2, $^{1}J_{CP}$ = 128 Hz, $^{2}J_{CF}$ = 24 Hz, $^{4}J_{CP}$ = 1 Hz), 128.6 (8C, dt, *m*-Ph, $^{3}J_{CP}$ = 16 Hz, $^{4}J_{CF}$ = 2 Hz), 131.7 (4C, d, *p*-Ph, $^{4}J_{CP}$ = 4 Hz), 134.2 (8C, dt, *o*-Ph, $^{2}J_{CP}$ = 12 Hz, $^{3}J_{CF}$ = 10 Hz), 136.5 (4C, dt, *i*-Ph, $^{1}J_{PF}$ = 172 Hz, $^{2}J_{CF}$ = 25 Hz); **$^{19}F\{^{1}H\}$ NMR (CD_2Cl_2, [ppm]):** δ = –38.9 (4F, X_2X_2’ part of AA’X_2X_2’ spin system, $^{1}J_{AX}$ = $^{1}J_{A'X'}$ = 641 Hz, $^{7}J_{A'X}$ = $^{7}J_{AX'}$ = 0 Hz, $^{8}J_{XX'}$ = 0 Hz); **$^{31}P\{^{1}H\}$ NMR (CD_2Cl_2, [ppm]):** δ = −42.1 (2P, AA’ part of AA’X_2X_2’ spin system, $^{5}J_{AA'}$ = 0 Hz); **elemental analysis** for $C_{29}H_{30}F_4P_2$: calcd.: C 67.4, H 5.9, found: C 67.8, H 6.2; **DART MS**: m/z: 497.2 (calcd. for $M{-}F^{+}$: 497.2).

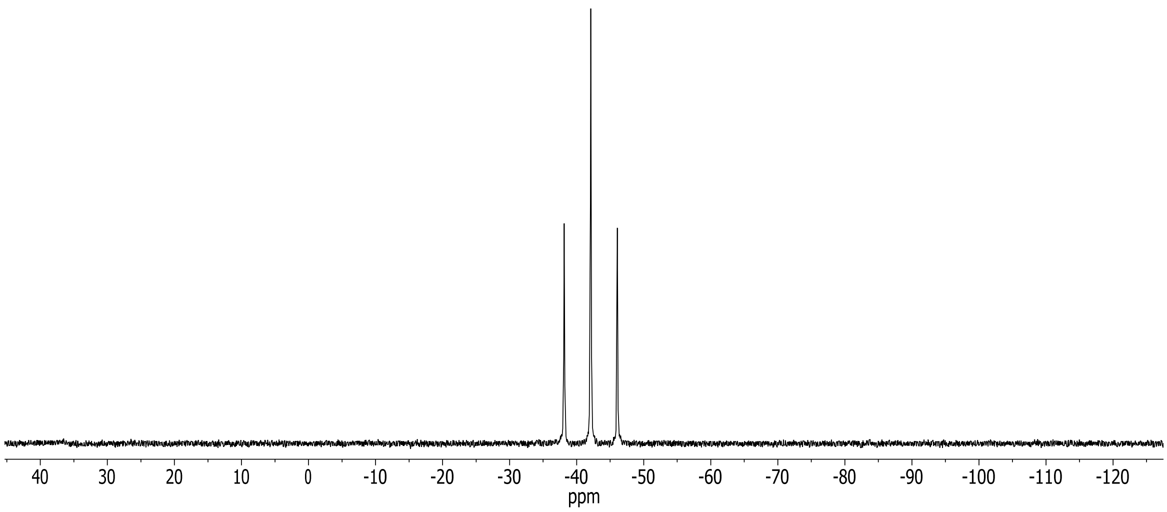

$^{31}P\{^1H\}$ NMR spectrum of compound **8e** (CD_2Cl_2).

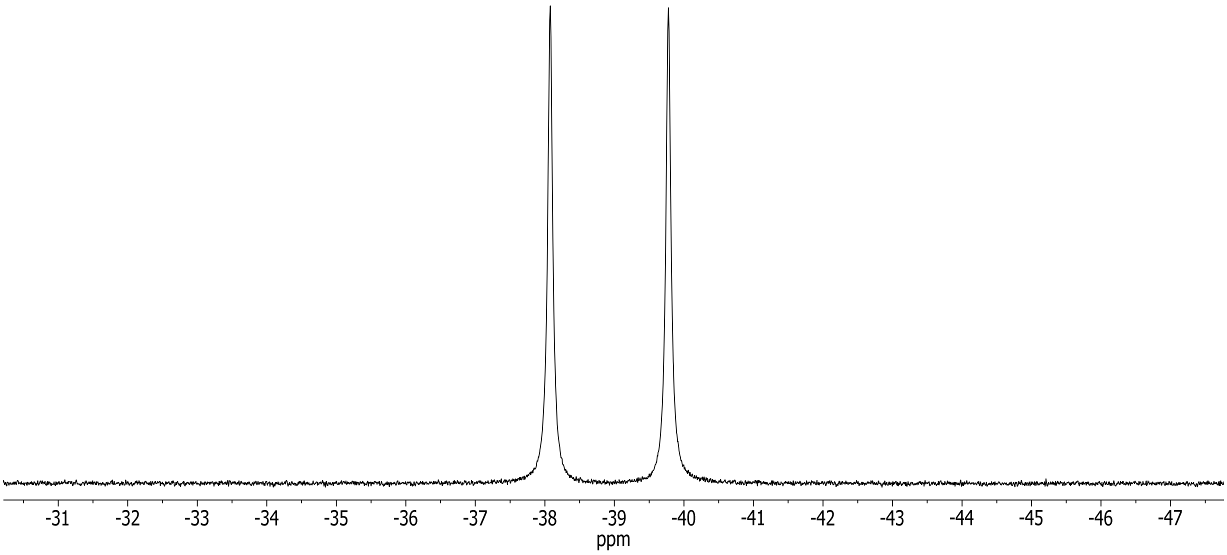

$^{19}F\{^1H\}$ NMR spectrum of compound **8e** (CD_2Cl_2).

2.11. Attempted stepwise oxidation of 1,1-bis(diphenylphosphino)methane

A solution of XeF_2 (15 mg, 0.089 mmol, 1.0 eq.) in CH_2Cl_2 (2 mL) was added dropwise to a solution of 1,1-bis(diphenylphosphino)methane (35 mg, 0.089 mmol, 1.0 eq.) in CH_2Cl_2 (2 mL) at −35 °C. The reaction mixture was stirred for one hour and all volatiles were removed *in vacuo* yielding a white solid. The residue was dissolved in CD_2Cl_2 and investigated by multi-nuclear magnetic resonance spectroscopy which indicated the presence of 1,1-bis(diphenylphosphino)methane, monophosphorane $(CH_2)_1(Ph_2P)(Ph_2PF_2)$ and bisphosphorane **8a** a 1 : 2 : 1 ratio.

^{1}H NMR (CD_2Cl_2, [ppm]): δ 2.91 (t, $(CH_2)_1(Ph_2P)_2$, $^2J_{HP}$ = 4 Hz), 3.19 (m, $(CH_2)_1(Ph_2P)(Ph_2PF_2)$), 3.63 (m, **8a**), 7.37 (8H, m, *m*-Ph), 7.32-7.98 ($(CH_2)_1(Ph_2P)_2$, $(CH_2)_1(Ph_2P)(Ph_2PF_2)$, **8a**); **^{19}F{^{1}H} NMR (CD_2Cl_2, [ppm]):** δ –32.0 (dd, $(CH_2)_1(Ph_2P)(Ph_2PF_2)$, $^1J_{FP}$ = 650 Hz, $^3J_{FP}$ = 16 Hz), –27.5 (m, **8a**); **^{31}P{^{1}H} NMR (CD_2Cl_2, [ppm]):** δ −46.8 (m, **8a**), −44.8 (td, $(CH_2)_1(Ph_2P)(Ph_2PF_2)$, $^1J_{PF}$ = 650 Hz, $^2J_{PP}$ = 63 Hz), δ -24.40 (dt, $(CH_2)_1(Ph_2P)(Ph_2PF_2)$, $^2J_{PP}$ = 63 Hz, $^3J_{PF}$ = 16 Hz,), -22.81 (s, $(CH_2)_1(Ph_2P)_2$).

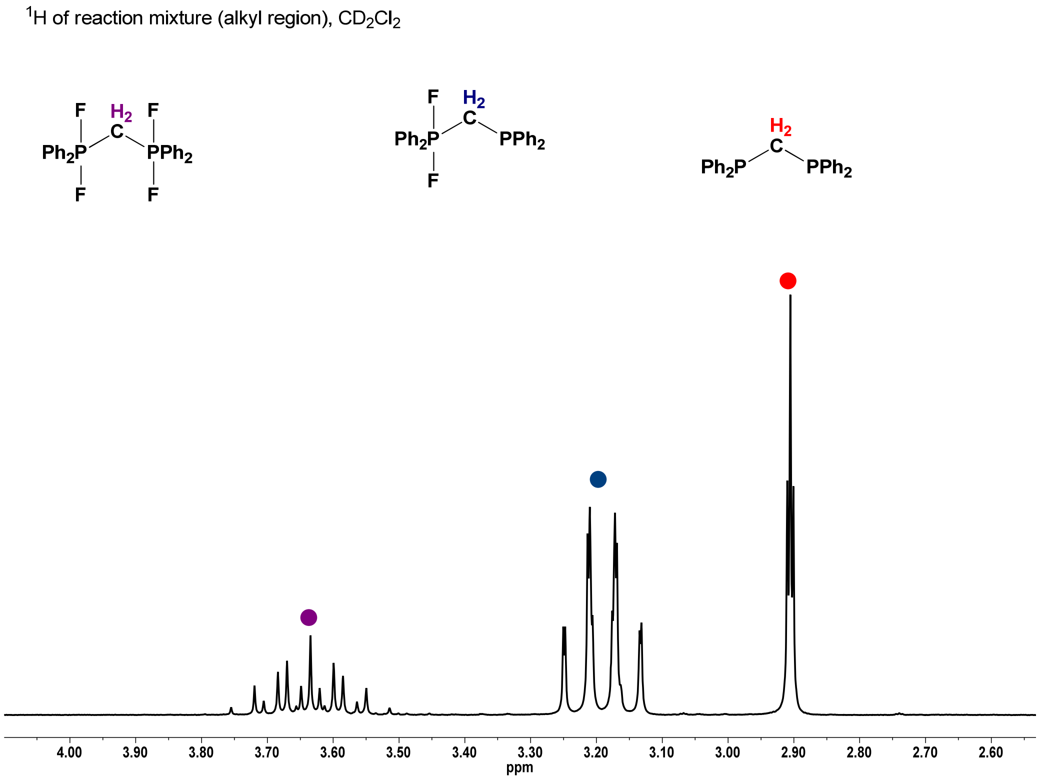

^{19}F of reaction mixture, CD_2Cl_2

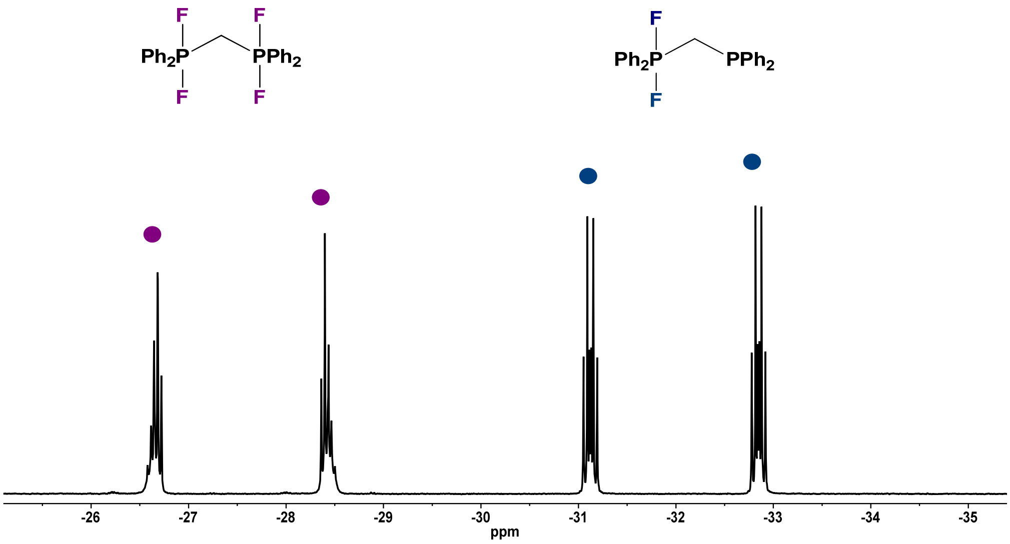

^{31}P {1H} of reaction mixture, CD_2Cl_2

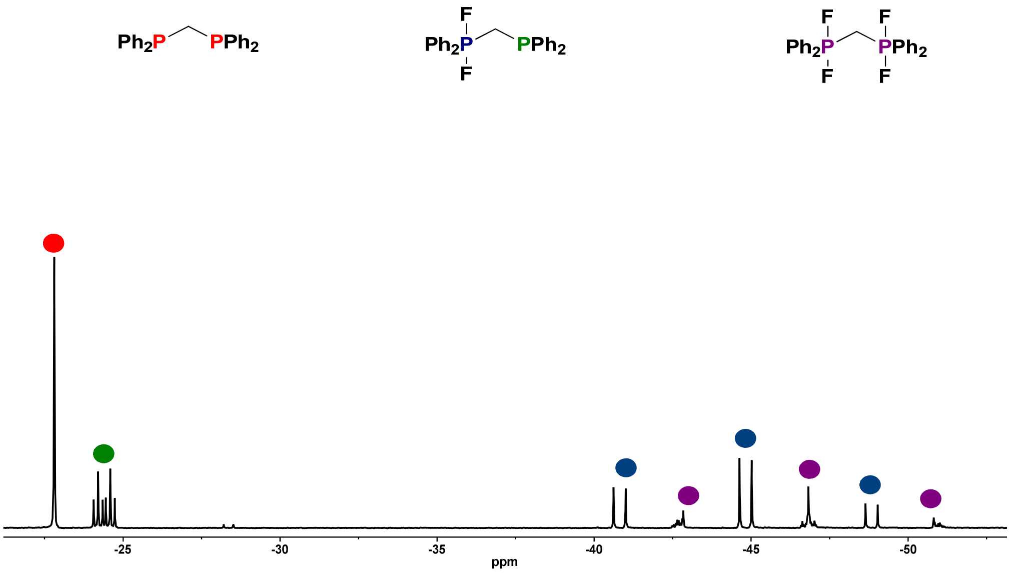

2.12. Preparation of $[(CH_2)_1(Ph_2PF)_2][B(C_6F_5)_4]_2$ (9a)

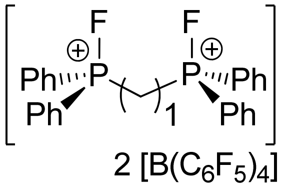

9a

Freshly prepared $[Et_3Si][B(C_6F_5)_4]*2(C_7H_8)$ (411 mg, 0.42 mmol, 1.8 eq.) was added to a solution of **8a** (104 mg, 0.23 mmol, 1.0 eq.) in toluene (5 mL). The formation of a brown oil was observed. The supernatant was removed and the residue was washed with *n*-pentane (3 x 3 mL). During this process, the oil turned into a white solid. The residue was dissolved in CH_2Cl_2 (2 mL) and addition of *n*-pentane (3 mL) resulted in the precipitation of **9a**. The supernatant was removed and the residue was dried *in vacuo* to afford **9a** as a white solid (349 mg, 93% yield).

^{1}H NMR (CD_2Cl_2, [ppm]): $\delta = 4.78$ (2H, m, CH_2), 7.73 (8H, m, *o*-Ph), 7.82 (8H, m, *m*-Ph), 8.15 (4H, t, *p*-Ph, $^3J_{HH}$ = 8 Hz); **^{11}B{^{1}H} NMR (CD_2Cl_2, [ppm]):** $\delta = -16.7$ (s); **^{13}C{^{1}H} NMR (CD_2Cl_2, [ppm]):** $\delta = 25.8$ (1C, tt, CH_2, $^1J_{CP}$ = 60 Hz, $^2J_{CF}$ = 16 Hz), 111.8 (4C, dm, *i*-Ph, $^1J_{CP}$ = 115 Hz), 123.8 (8C, m(br), *i*-C_6F_5), 132.0 (8C, dm, *m*-Ph, $^3J_{CP}$ = 15 Hz), 134.9 (8C, dm, *o*-Ph, $^2J_{CP}$ = 14 Hz), 136.2 (16C, dt, *p*-C_6F_5, $^1J_{CF}$ = 241 Hz, $^3J_{FF}$ = 14 Hz), 138.1 (8C, dt, *p*-C_6F_5, $^1J_{CF}$ = 238 Hz, $^3J_{FF}$ = 12 Hz), 148.1 (16C, d(br), $^1J_{CF}$ = 238 Hz); **^{19}F{^{1}H} NMR (CD_2Cl_2, [ppm]):** $\delta = -167.1$ (16F, m, *m*-C_6F_5), −163.1 (8F, m, *p*-C_6F_5), −133.0 (16F, m, *o*-C_6F_5), −126.3 (2F, XX’ part of AA’XX’ spin system, $^1J_{AX} = {}^1J_{A'X'}$ = 1024 Hz, $^3J_{A'X} = {}^3J_{AX'}$ = 3 Hz, $^4J_{XX'}$ = 12 Hz); **^{31}P{^{1}H} NMR (CD_2Cl_2, [ppm]):** $\delta = 94.5$ (2P, AA’ part of AA’XX’ spin system, $^5J_{AA'}$ = 20 Hz); **elemental analysis** for $C_{73}H_{22}B_2F_{42}P_2$: calcd.: C 49.2, H 1.2, found: C 49.2, H 1.1; **ESI MS**: m/z: 417.1161 (calcd. for $[M{-}2F{+}HO_2]^+$: 417.1173)

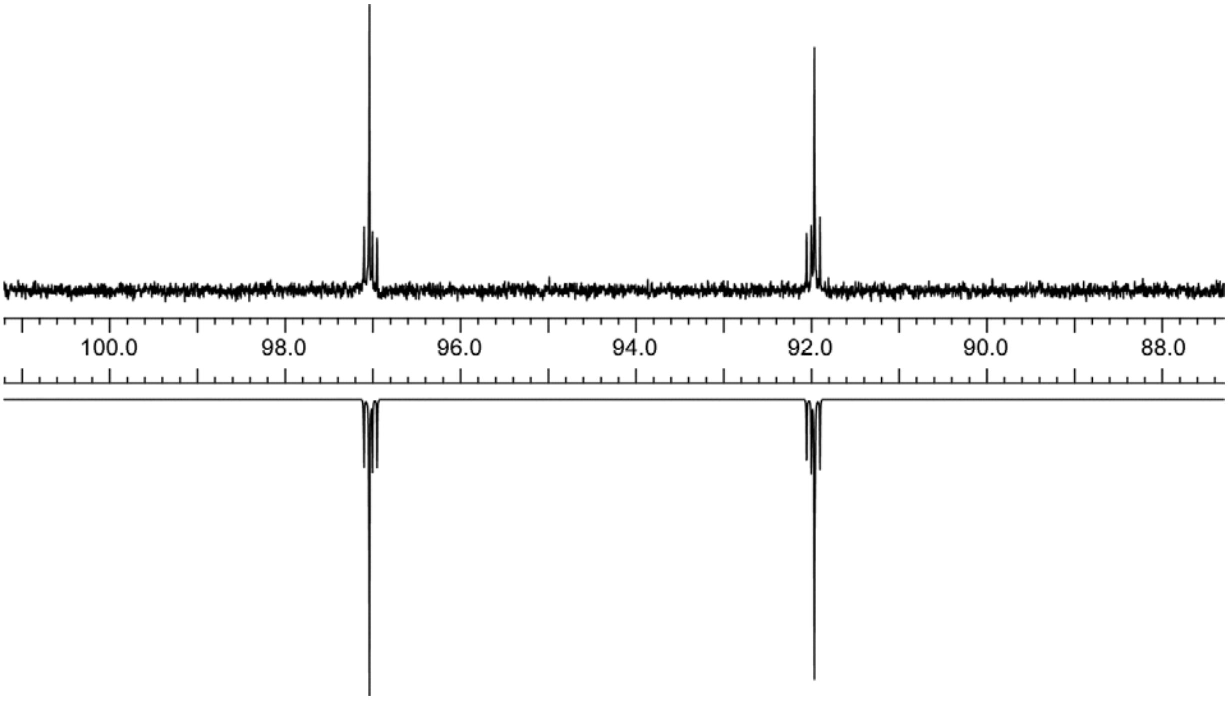

$^{31}P\{^1H\}$ NMR spectrum of compound **9a** (CD_2Cl_2, upwards) and simulated spectrum (downwards).

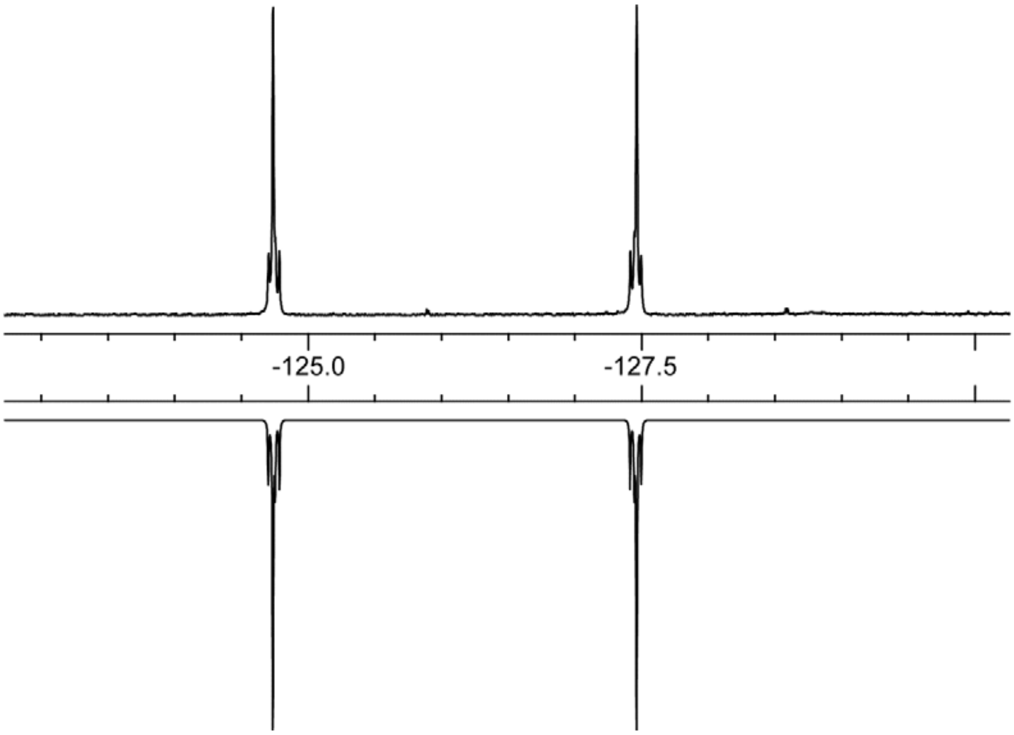

$^{19}F\{^1H\}$ NMR spectrum of compound **9a** (CD_2Cl_2, upwards) and simulated spectrum (downwards).

2.13. Preparation of $[(CH_2)_2(Ph_2PF)_2][B(C_6F_5)_4]_2$ (9b)

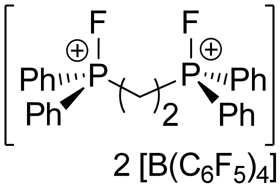

9b

Freshly prepared $[Et_3Si][B(C_6F_5)_4]$*$2(C_7H_8)$ (283 mg, 0.29 mmol, 1.7 eq.) was added to a solution of **9b** (81 mg, 0.17 mmol, 1.0 eq.) in toluene (5 mL). The solution was stirred for 24 hours, the supernatant was removed, and the residue was washed with *n*-pentane (3 x 3 mL). During this process, the oil turned into a white solid. The residue was dissolved in CH_2Cl_2 (2 mL) and addition of *n*-pentane (3 mL) resulted in the precipitation of **9b**. The supernatant was removed and the residue was dried *in vacuo* to afford **9b** as a white solid (242 mg, 93% yield).

^{1}H NMR (CD_3CN, [ppm]): δ = 3.69 (4H, m, CH_2), 7.85 (8H, m, *o*-Ph), 7.98 (8H, m, *m*-Ph), 8.07 (4H, t, *p*-Ph, $^3J_{CF}$ = 8 Hz); **^{11}B{^{1}H} NMR (CD_3CN, [ppm]):** δ = −16.7 (s); **^{13}C{^{1}H} NMR (CD_3CN, [ppm]):** δ = 18.5 (2C, dd, CH_2, $^1J_{CP}$ = 60 Hz, $^2J_{CF}$ = 11 Hz), 115.5 (4C, dd, *i*-Ph, $^1J_{CP}$ = 103 Hz, $^2J_{CF}$ = 13 Hz), 125.0 (8C, m(br), *i*-C_6F_5), 131.7 (8C, d, *m*-Ph, $^3J_{CP}$ = 15 Hz), 134.7 (8C, dm, *o*-Ph, $^2J_{CP}$ = 13 Hz), 137.3 (16C, dm, C_6F_5, $^1J_{CF}$ = 241 Hz), 139.3 (8C, dm, C_6F_5, $^1J_{CF}$ = 244 Hz), 139.0 (4C, s, *p*-Ph), 149.1 (16C, d(br), C_6F_5 $^1J_{CF}$ = 235 Hz); **^{19}F{^{1}H} NMR (CD_3CN, [ppm]):** δ = −168.3 (16F, m, *m*-C_6F_5), −163.9 (8F, m, *p*-C_6F_5), −138.2 (2F, XX' part of AA'XX' spin system, $^1J_{AX} = {}^1J_{A'X'}$ = 1008 Hz, $^4J_{A'X} = {}^4J_{AX'}$ = 0 Hz, $^5J_{XX'}$ = 0 Hz), −133.7 (16F, m, *o*-C_6F_5); **^{31}P{^{1}H} NMR (CD_3CN, [ppm]):** δ = 106.55 (2P, AA' part of AA'XX' spin system, $^3J_{AA'}$ = 70 Hz); **elemental analysis** for $C_{73}H_{22}B_2F_{42}P_2$: calcd.: C 49.5, H 1.35, found: 49.8, H 1.9; **ESI MS**: m/z: 432.1 (calcd. for $[M{-}2F{+}H_2O_2]^+$: 432.1)

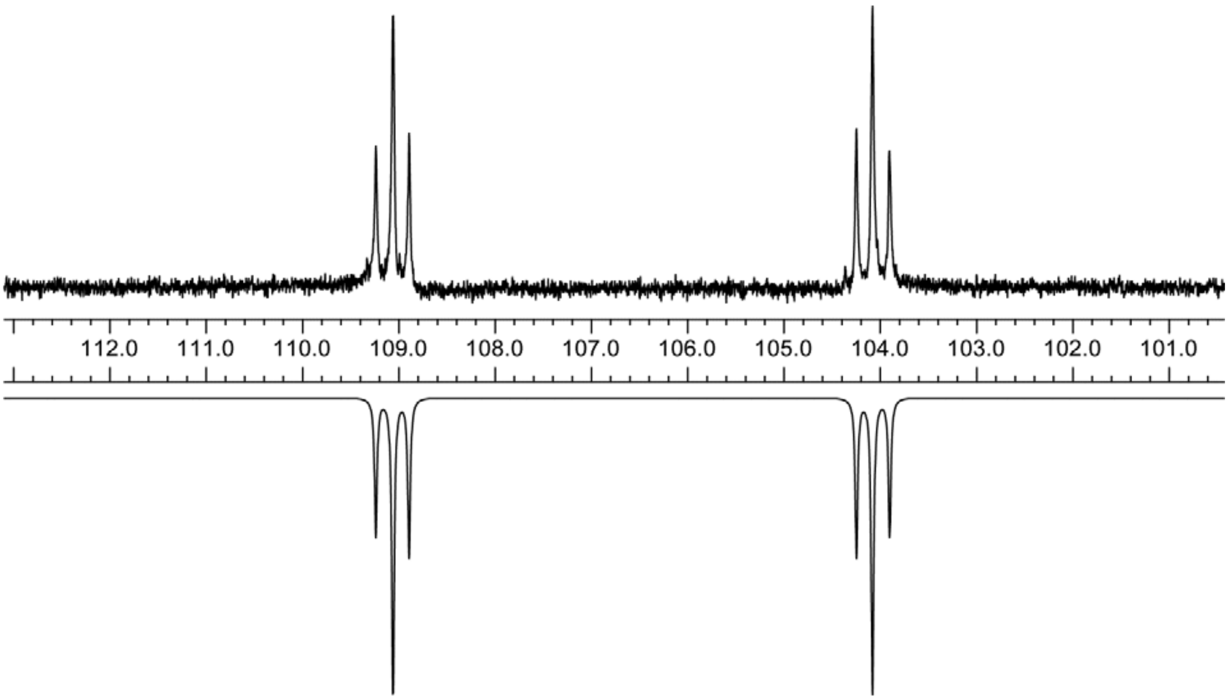

$^{31}P\{^1H\}$ NMR spectrum of compound **9b** (CD_3CN, upwards) and simulated spectrum (downwards).

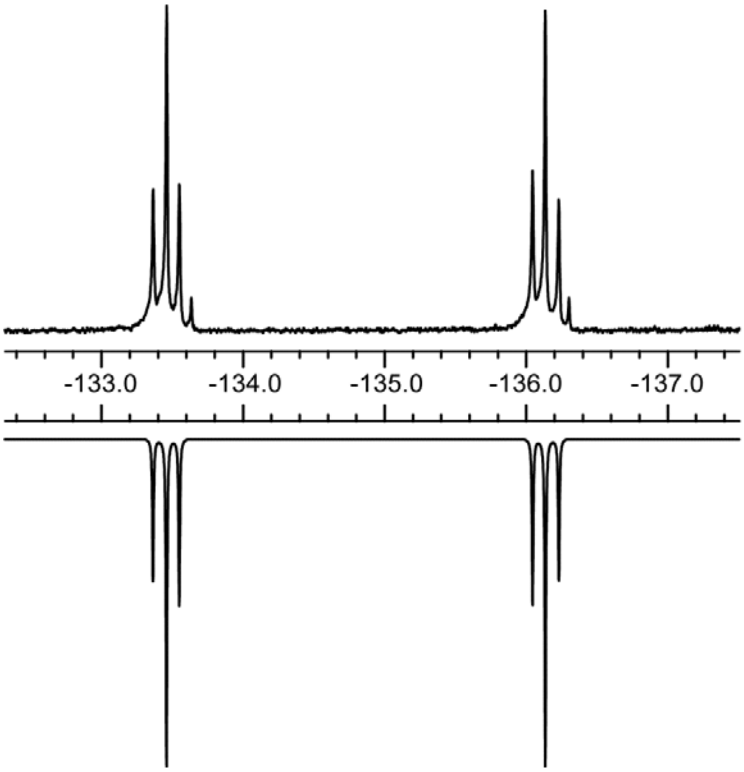

$^{19}F\{^1H\}$ NMR spectrum of compound **9b** (CD_3CN, upwards) and simulated spectrum (downwards).

2.14. Preparation of $[(CH_2)_3(Ph_2PF)_2][B(C_6F_5)_4]_2$ (9c)

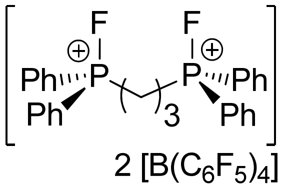

9c

Freshly prepared $[Et_3Si][B(C_6F_5)_4]$*2(C_7H_8) (176 mg, 0.18 mmol, 1.8 eq.) was added to a solution of **8c** (49 mg, 0.10 mmol, 1.0 eq.) in toluene (5 mL). The formation of a brown oil was observed. The supernatant was removed and the residue was washed with *n*-pentane (3 x 3 mL). During this process, the oil turned into a white solid. The solid was dissolved in dichloromethane (2 mL) and addition of *n*-pentane (3 mL) resulted in the precipitation of **9c**. The supernatant was removed and the residue was dried *in vacuo* to afford **9c** as a white solid (154 mg, 95% yield).

^{1}H NMR (CD_2Cl_2, [ppm]): $\delta = 2.25$ (2H, m (br), CH_2), 3.20 (4H, br, P-CH_2), 7.78 (8H, br, *o*-Ph), 7.81 (8H, br, *m*-Ph), 8.11 (4H, br, *p*-Ph); **^{11}B{^{1}H} NMR (CD_2Cl_2, [ppm]):** $\delta = -16.7$ (s); **^{13}C{^{1}H} NMR (CD_2Cl_2, [ppm]):** $\delta = 14.1$ (1C, br, CH_2), 25.9 (2C, d (br), CH_2, $^1J_{CP} = 63$ Hz), 113.8 (4C, dd, *i*-Ph, $^1J_{CP} = 108$ Hz, $^2J_{CF} = 18$ Hz), 123.8 (8C, m(br), *i*-C_6F_5), 131.8 (8C, m, *m*-Ph), 134.9 (8C, dm, *o*-Ph, $^2J_{CP} = 14$ Hz), 136.6 (16C, dm, C_6F_5, $^1J_{CF} = 249$ Hz), 138.5 (8C, dm, C_6F_5, $^1J_{CF} = 246$ Hz), 148.4 (16C, d(br), C_6F_5 $^1J_{CF} = 244$ Hz); **^{19}F{^{1}H} NMR (CD_2Cl_2, [ppm]):** $\delta = -167.2$ (16F, m, *m*-C_6F_5), −163.1 (8F, m, *p*-C_6F_5), −138.9 (2F, XX' part of AA'XX' spin system, $^1J_{AX} = {}^1J_{A'X'} = 1002$ Hz, $^5J_{A'X} = {}^5J_{AX'} = -1$ Hz, $^6J_{XX'} = 0$ Hz), −133.0 (16F, m, *o*-C_6F_5); **^{31}P{^{1}H} NMR (CD_2Cl_2,[ppm]):** $\delta = 104.88$ (2P, AA' part of AA'XX' spin system, $^4J_{AA'} = 7.5$ Hz); **elemental analysis** for $C_{75}H_{26}B_2F_{42}P_2$: calcd.: C 49.8, H 1.45,found: C 49.6, H 1.8; **ESI MS**: m/z: 445.1 (calcd. for $[M-2F+HO_2]^+$: 459.2).

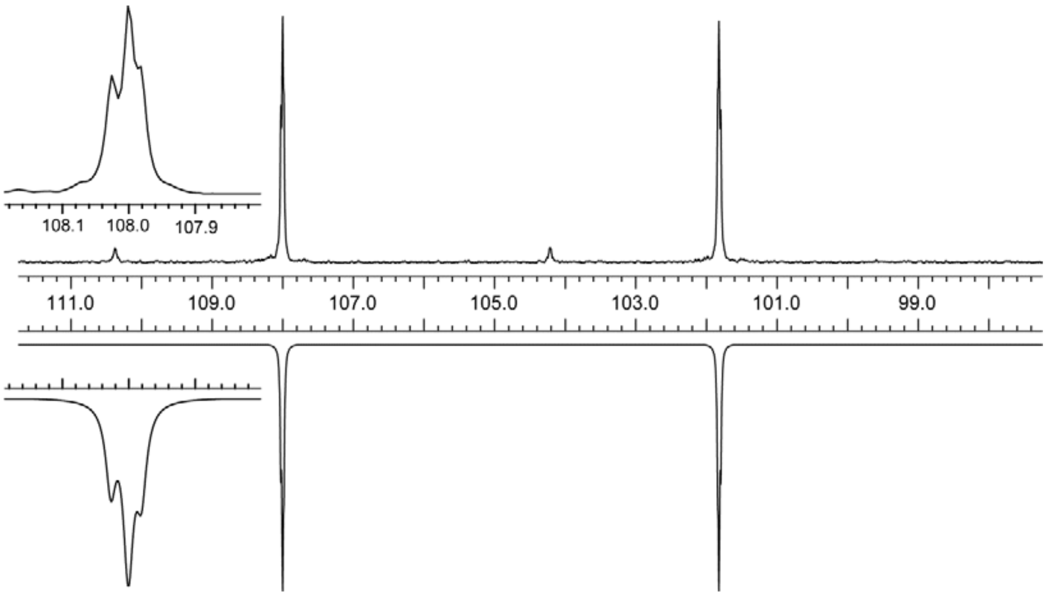

$^{31}P\{^{1}H\}$ NMR spectrum of compound **9c** (CD_2Cl_2, upwards) and simulated spectrum (downwards).

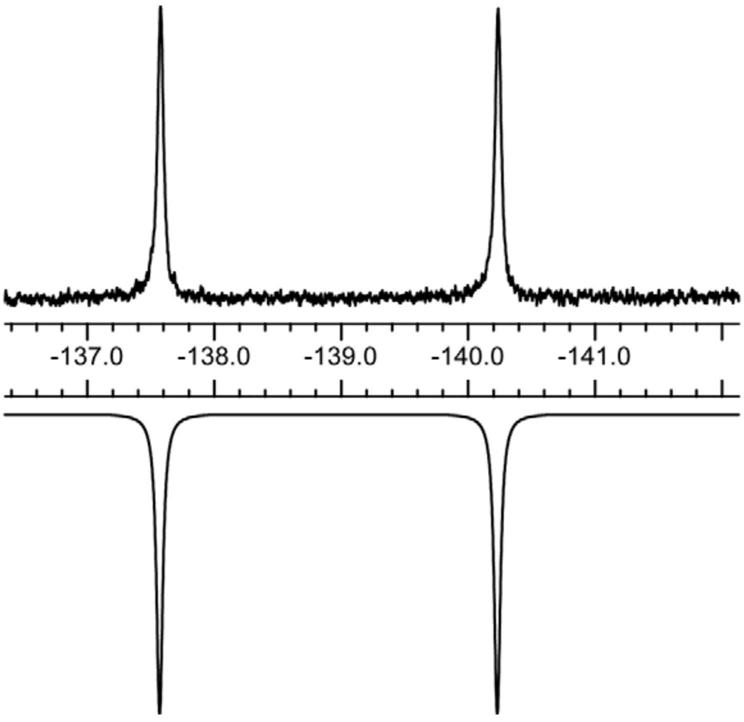

$^{19}F\{^{1}H\}$ NMR spectrum of compound **9c** (CD_2Cl_2, upwards) and simulated spectrum (downwards).

2.15. Preparation of $[(CH_2)_4(Ph_2PF)_2][B(C_6F_5)_4]_2$ (9d)

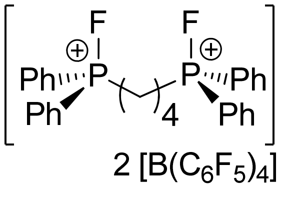

9d

Freshly prepared $[Et_3Si][B(C_6F_5)_4]*2(C_7H_8)$ (254 mg, 0.26 mmol, 1.7 eq.) was added to a solution of **8d** (75 mg, 0.15 mmol, 1.0 eq.) in toluene (5 mL). The formation of a brown oil was observed. The supernatant was removed and the residue was washed with *n*-pentane (3 x 3 mL). During this process, the oil turned into a white solid. The residue was dissolved in dichloromethane (2 mL) and addition of *n*-pentane (3 mL) resulted in the precipitation of **9d**. The supernatant was removed and the residue was dried *in vacuo* to afford **9d** as a white solid (201 mg, 85% yield).

^{1}H NMR (CD_3CN, [ppm]): δ = 1.90 (4H, m, CH_2), 3.27 (4H, m, P-CH_2), 7.80 (8H, m, *o*-Ph), 7.92 (8H, m, *m*-Ph), 8.11 (4H, tm, *p*-Ph, $^{3}J_{CH}$ = 8 Hz); **^{11}B{^{1}H} NMR (CD_2Cl_2, [ppm]):** δ = −16.6 (s); **^{13}C{^{1}H} NMR (CD_3CN, [ppm]):** δ = 22.0 (2C, dd, CH_2, $^{2}J_{CP}$ = 19 Hz, $^{3}J_{CF}$ = 4 Hz), 24.3 (2C, ddm, P-CH_2, $^{1}J_{CP}$ = 61 Hz, $^{2}J_{CF}$ = 12 Hz), 117.3 (4C, dd, *i*-Ph, $^{1}J_{CP}$ = 101 Hz, $^{2}J_{CF}$ = 12 Hz), 124.9 (8C, m(br), *i*-C_6F_5), 131.4 (8C, d, *m*-Ph, $^{3}J_{CP}$ = 14 Hz), 134.2 (8C, dd, *o*-Ph, $^{2}J_{CP}$ = 12 Hz, $^{3}J_{CF}$ = 1 Hz), 137.4 (16C, dm, C_6F_5, $^{1}J_{CF}$ = 244 Hz), 139.3 (8C, dm, C_6F_5, $^{1}J_{CF}$ = 244 Hz), 139.0 (4C, m, *p*-Ph), 149.2 (16C, d(br), C_6F_5 $^{1}J_{CF}$ = 239 Hz); **^{19}F{^{1}H} NMR (CD_2Cl_2, [ppm]):** δ = −167.3 (16F, m, *m*-C_6F_5), −163.3 (8F, m, *p*-C_6F_5), −139.3 (2F, XX' part of AA'XX' spin system, $^{1}J_{AX}$ = $^{1}J_{A'X'}$ = 999 Hz, $^{6}J_{A'X}$ = $^{6}J_{AX'}$ = 0 Hz, $^{7}J_{XX'}$ = 0 Hz), −133.0 (16F, m, *o*-C_6F_5); **^{31}P{^{1}H} NMR (CD_2Cl_2, [ppm]):** δ = 106.13 (2P, AA' part of AA'XX' spin system, $^{5}J_{AA'}$ = 0 Hz); **elemental analysis** for $C_{76}H_{28}B_2F_{42}P_2$: calcd.: C 50.1, H 1.55, found: C 50.2, H 1.54; **ESI MS**: m/z: 459.1640 (calcd. for $[M{-}2F{+}HO_2]^{+}$: 459.1637).

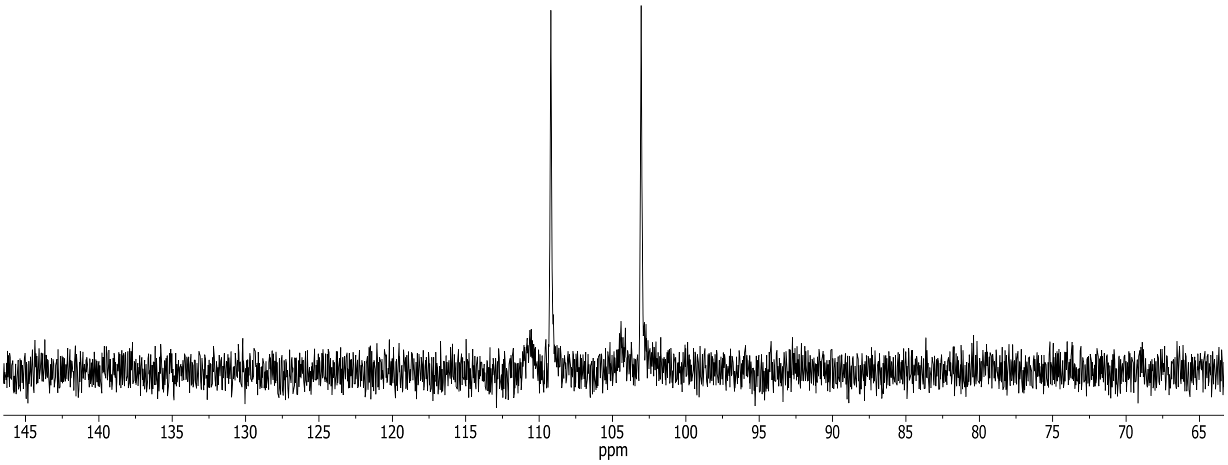

$^{31}P\{^{1}H\}$ NMR spectrum of compound **9d** (CD_2Cl_2).

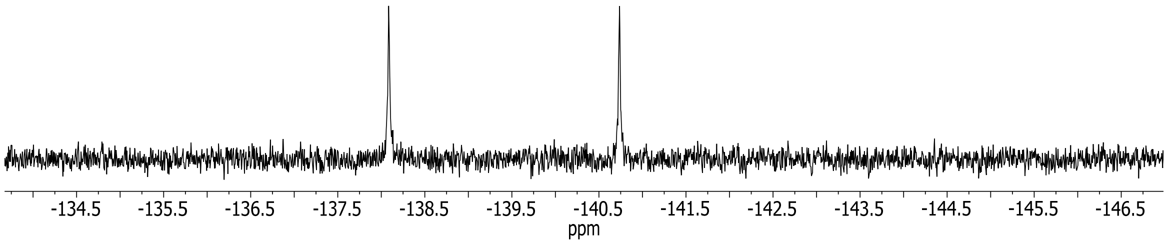

$^{19}F\{^{1}H\}$ NMR spectrum of compound **9d** (CD_2Cl_2).

2.16. Preparation of $[(CH_2)_5(Ph_2PF)_2][B(C_6F_5)_4]_2$ (9e)

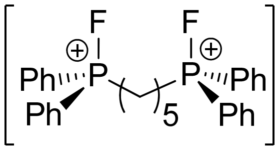

2 $[B(C_6F_5)_4]$

9e

Freshly prepared $[Et_3Si][B(C_6F_5)_4]$*2(C_7H_8) (343 mg, 0.35 mmol, 1.5 eq.) was added to a solution of **8e** (117 mg, 0.23 mmol, 1.0 eq.) in toluene (5 mL). The formation of a brown oil was observed. The supernatant was removed and the residue was washed with *n*-pentane (3 x 3 mL). During this process, the oil turned into a white solid. The residue was dissolved in dichloromethane (2 mL) and addition of *n*-pentane (3 mL) resulted in the precipitation of **9e**. The supernatant was removed and the residue was dried *in vacuo* to afford **9e** as a white solid (301 mg, 94% yield).

^{1}H NMR (CD_2Cl_2, [ppm]): δ = 1.73 (6H, m (br), CH_2), 2.96 (4H, br, P-CH_2), 7.78 (16H, br, *o*-Ph, *m*-Ph), 8.03 (4H, br, *p*-Ph); **^{11}B{^{1}H} NMR (CD_2Cl_2, [ppm]):** δ = −16.7 (s); **^{13}C{^{1}H} NMR (CD_2Cl_2, [ppm]):** δ = 20.81 (1C, br, CH_2), 25.1 (2C, d (br), CH_2, $^{1}J_{CP}$ = 69 Hz), δ 31.38 (2C, br, CH_2), 115.3 (4C, dm, *i*-Ph, $^{1}J_{CP}$ = 102 Hz), 124.3 (8C, m(br), *i*-C_6F_5), 131.5 (8C, d, *m*-Ph, $^{2}J_{CP}$ = 14 Hz), 132.8 (8C, d, *o*-Ph, $^{2}J_{CP}$ = 11 Hz), 136.7 (16C, dm, C_6F_5, $^{1}J_{CF}$ = 240 Hz), 138.6 (8C, dm, C_6F_5, $^{1}J_{CF}$ = 246 Hz), 139.2 (8C, s, *p*-Ph), 148.5 (16C, d(br), C_6F_5 $^{1}J_{CF}$ = 238 Hz); **^{19}F{^{1}H} NMR (CD_2Cl_2, [ppm]):** δ = −167.3 (16F, m, *m*-C_6F_5), −163.3 (8F, m, *p*-C_6F_5), −139.7 (2F, XX' part of AA'XX' spin system, $^{1}J_{AX}$ = $^{1}J_{A'X'}$ = 998 Hz, $^{7}J_{A'X}$ = $^{7}J_{AX'}$ = 0 Hz, $^{8}J_{XX'}$ = 0 Hz), −133.0 (16F, m, *o*-C_6F_5); **^{31}P{^{1}H} NMR (CD_2Cl_2, [ppm]):** δ = 107.0 (2P, AA' part of AA'XX' spin system, $^{6}J_{AA'}$ = 0 Hz); **elemental analysis** for $C_{77}H_{30}B_2F_{42}P_2$: calcd.: C 50.4, H 1.6, found: C 51.4, H 1.1; **ESI MS**: m/z: 473.2 (calcd. for $[M{-}2F{+}HO_2]^{+}$: 473.2).

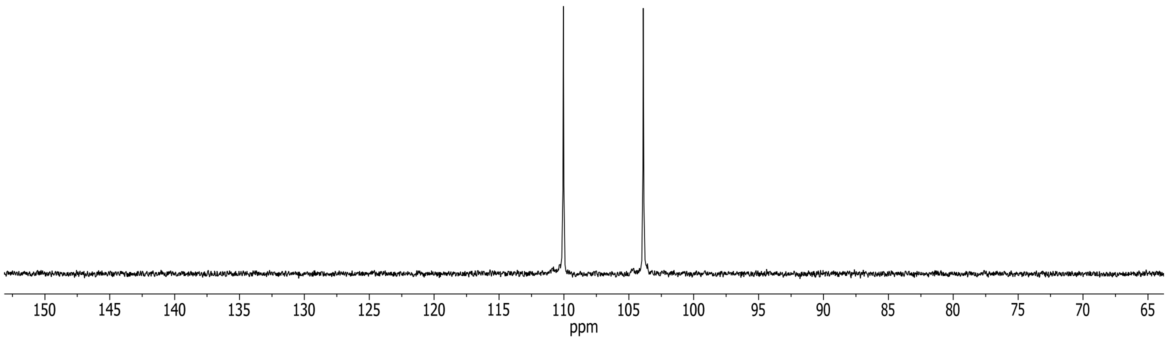

$^{31}P\{^1H\}$ NMR spectrum of compound **9e** (CD_2Cl_2).

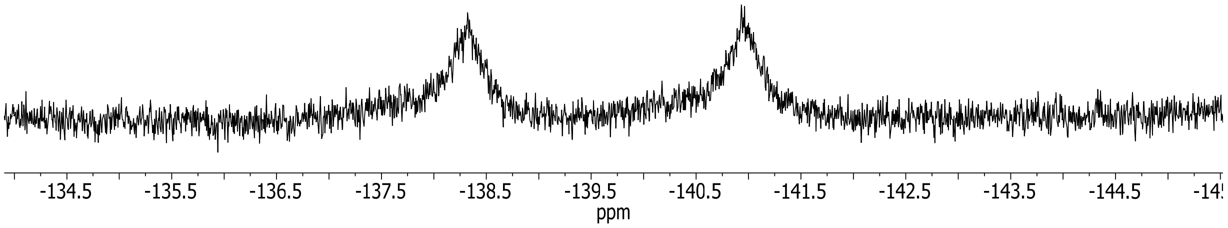

$^{19}F\{^1H\}$ NMR spectrum of compound **9e** (CD_2Cl_2).

2.17. ^{31}P{^{1}H} and ^{19}F{^{1}H} NMR parameters of 8a-e and 9a-e

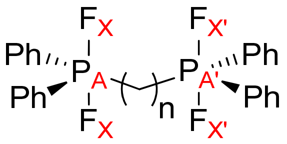

8a-e

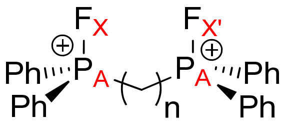

9a-e

**8**	Spin system AA'X_2X_2'					
	A (^{31}P)	X (^{19}F)	$^{1}J_{AX} = {}^{1}J_{A'X'}$	$^{n+2}J_{AX} = {}^{n+2}J_{A'X}$	$^{n+1}J_{AA'}$	$^{n+3}J_{XX'}$
**a**	−46.8	−27.7	664	−18	26	−7
**b**	−43.6	−39.7	653	−6	115	-
**c**	−43.2	−39.0	644	−2	12	-
**d**	−42.4	−38.9	641	-	-	-
**e**	−42.1	−38.9	641	-	-	-
**9**	Spin system AA'XX'					
	A (^{31}P)	X (^{19}F)	$^{1}J_{AX} = {}^{1}J_{A'X'}$	$^{n}J_{AX} = {}^{n}J_{A'X}$	$^{n}J_{AA'}$	$^{n}J_{XX'}$
**a**	94.5	−126.3	1024	3	20	12
**b**	106.4	−134.8	1008	-	70	-
**c**	104.9	−138.9	1002	−1	8	-
**d**	106.1	−139.4	999	-	-	-
**e**	107.0	−139.7	997	-	-	-

Note: Compounds **8a-c** and **9a-c** showed higher order resonances and their ^{31}P and ^{19}F NMR parameters (CD_2Cl_2) were obtained by means of full-lineshape iteration,[S7] spectra for **9b** were recorded in CD_3CN solution.

Discussion: The ^{31}P NMR resonances of phosphoranes **8a-e** are shifted to higher field with increased length of the (oligo)methylene-linker. A similar trend is observed for the ^{19}F NMR chemical shifts of bisphosphonium ions **9a-e**. For both substance classes, a decrease in the value of the $^{1}J_{PF}$ coupling constant occurs with an increase in (oligo)methylene-linker length. Commonly, highly electrophilic phosphonium ions show $^{1}J_{PF}$ coupling constants above 1000 Hz.[S8] Thus, significant electrophilicity is anticipated for methylene-bridged compound **9a** ($^{1}J_{AX}$ = 1024 Hz) and a stepwise decreases upon utilization of longer (oligo)methylene-linkers seems likely. Only methylene bridged compounds **8a**/**9a** reveal a significant coupling between fluorine atoms ($^{4}J_{XX'}$ = −7 Hz and 12 Hz) and observable P-P couplings occur up to propylene-linked compounds **8c**/**9c**. Remarkable large P-P coupling constants are observed for ethylene-

linked compounds **8b**/**9b** ($^{3}J_{AA'}$ = 114 Hz and 70 Hz) which might indicate a weak through space interaction between both P moieties.[S9]

2.18. Preparation of Ph_3PF_2[S10]

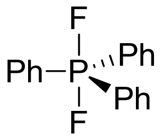

XeF_2 (56 mg, 0.33 mmol, 1.1 eq.) was added portion wise to a solution of triphenylphosphine (79 mg, 0.30 mmol, 1.0 eq.) in CH_2Cl_2 (10 mL). The reaction mixture was stirred for 30 min at ambient temperature. Removal of all volatiles *in vacuo* gave Ph_3PF_2 as colourless solid (92 mg, 99%). Multi-nuclear magnetic resonance experiments were in accordance to literature reported values.[S10]

^{1}H NMR (CD_2Cl_2, [ppm]): δ = 7.42 - 7.58 (9H, m), 7.94 - 8.06 (6H, m); **^{19}F{^{1}H} NMR (CD_2Cl_2, [ppm]):** δ = −39.6 (d, $^{1}J_{FP}$ = 660 Hz); **^{31}P{^{1}H} NMR (CD_2Cl_2, [ppm]):** δ = −54.9 (t, $^{1}J_{PF}$ = 660 Hz).

2.19. Preparation of $[Ph_3PF][B(C_6F_5)_4]$[S11]

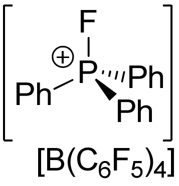

Freshly prepared $[Et_3Si][B(C_6F_5)_4]$*2(C_7H_8) (139 mg, 0.14 mmol, 1.0 eq.) was added to a solution of Ph_3PF_2 (42 mg, 0.14 mmol, 1.0 eq.) in C_6H_5F (5 mL). Addition of *n*-pentane (5 mL) to the reaction mixture led to the formation of a colourless precipitate. The supernatant was removed and the residue was washed with *n*-pentane (3 x 3 mL). Removal of all volatiles *in vacuo* gave $[Ph_3F][B(C_6F_5)_4]$ as a colourless, microcrystalline solid (119 mg, 87% yield). Multi-nuclear magnetic resonance experiments were in accordance to literature reported values of related AsF_6^- and BF_4^- salts.[S11]

^{1}H NMR (CD_2Cl_2, [ppm]): δ = 7.72 - 7.88 (12H, *m*-/*o*-Ph), 8.02 - 8.10 (3H, *p*-Ph); **^{11}B{^{1}H} NMR (CD_2Cl_2, [ppm]):** δ = −16.7 (s); **^{13}C{^{1}H} NMR (CD_2Cl_2, [ppm]):** δ = 131.3 (6C, d, *o*-/*m*-Ph, J_{CP} = 14.4 Hz), 134.3 (6C, dd, *o*-/*m*-Ph, J_{CP} = 13.2 Hz, J_{CF} = 1.1 Hz), 139.0 (3C, dd, *p*-Ph, $J_{CP/F}$ = 2.7 Hz, 1.8 Hz), 116.5 (3C, dd, *i*-Ph, $^{1}J_{CP}$ = 109.0 Hz, $^{2}J_{CF}$ = 14.6 Hz), 131.3 (6C, d, *o*-/*m*-Ph, J_{CP} = 14.6 Hz), 134.3 (6C, dd, *o*-/*m*-Ph, J_{CP} = 13.1 Hz, J_{CF} = 1.3 Hz), 136.7 (8C, d(br), C_6F_5, $^{1}J_{CF}$ = 245 Hz), 138.6 (4C, d(br), C_6F_5, $^{1}J_{CF}$ = 247 Hz), 139.0 (3C, dd, *p*-Ph, $^{4}J_{CP}$ = 2.8 Hz, $^{5}J_{CF}$ = 1.7 Hz), 148.5 (8C, d(br), C_6F_5, $^{1}J_{CF}$ = 241 Hz); **^{19}F{^{1}H} NMR (CD_2Cl_2, [ppm]):** δ = −167.6 (8F, m, *m*-C_6F_5), −163.7 (4F, t, *p*-C_6F_5, $^{3}J_{FF}$ = 20 Hz), −133.2 (8F, m, *o*-C_6F_5), −128.1 (d, $^{1}J_{FP}$ = 997 Hz); **^{31}P{^{1}H} NMR (CD_2Cl_2, [ppm]):** δ = −94.8 (d, $^{1}J_{PF}$ = 997 Hz).

2.20. Reaction of 5, $[Ph_3PF][B(C_6F_5)_4]$, and 9a-e with Et_3PO (Gutmann-Beckett test)

Et_3PO (4 mg, 0.03 mmol, 1.0 eq.) was added to solutions of **5** (57 mg, 0.03 mmol, 1.0 eq.), $[Ph_3PF][B(C_6F_5)_4]$, (58 mg, 0.06 mmol, 1.0 eq.) or **9a-e** (**a**: 53 mg, **b**: 54 mg, **c**: 54 mg, **d**: 55 mg, **e**: 55 mg, 0.03 mmol, 1.0 eq.) in CD_2Cl_2 (1 mL). The reaction mixtures were monitored by means of ^{31}P and ^{19}F NMR spectroscopy for 20 h at ambient temperature. The formation of **6**[S8b] and Ph_3PO[S12] was confirmed by comparison with literature known NMR data.

5 + Et_3PO (1 h):

^{19}F{^{1}H} NMR (CD_2Cl_2, [ppm]): $\delta = -117.2$ (d, **5**, $^1J_{FP} = 991$ Hz); **^{31}P{^{1}H} NMR (CD_2Cl_2, [ppm]):** $\delta = 60.5$ (s(br), Et_3PO), 96.7 (dd, **5**, $^1J_{PF} = 991$ Hz, $^5J_{PF} = 13$ Hz).

5 + Et_3PO (20 h):

^{19}F NMR (CD_2Cl_2, [ppm]): $\delta = -158.5$ (dsept., **6**, $^1J_{FP} = 964$ Hz, $^3J_{FH} = 12$ Hz), -24.5 (d(br), **7**, $^1J_{FP} = 697.0$ Hz); **^{31}P{^{1}H} NMR (CD_2Cl_2, [ppm]):** $\delta = -34.5$ (dd, **7**, $^1J_{FP} = 697$ Hz, $^2J_{PP} = 23$ Hz), 47.1 (dd, **7**, $^2J_{PP} = 23$ Hz, $^3J_{PF} = 2$ Hz), 147.9 (d, **6**, $^1J_{PF} = 964$ Hz).

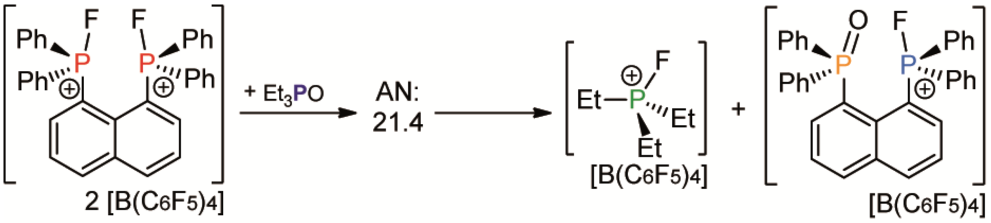

5 **6** **7**

20 h: 59% conversion

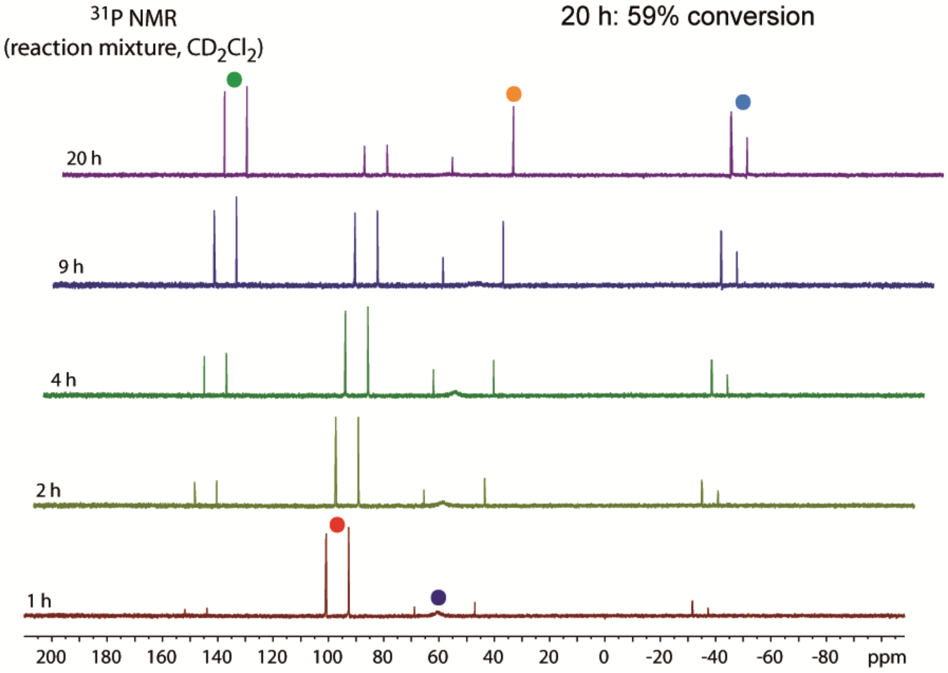

$[Ph_3PF][B(C_6F_5)_4]$ + Et_3PO (1 h):

$^{19}F\{^1H\}$ NMR (CD_2Cl_2, [ppm]): $\delta = -128.1$ (d, $[Ph_3PF]^+$, $^1J_{FP} = 998$ Hz); **$^{31}P\{^1H\}$ NMR (CD_2Cl_2, [ppm]):** $\delta = 51.1$ (s(br), Et_3PO), 94.8 (d, $[Ph_3PF]^+$, $^1J_{PF} = 998$ Hz).

$[Ph_3PF][B(C_6F_5)_4]$ + Et_3PO (20 h):

^{19}F NMR (CD_2Cl_2, [ppm]): $\delta = -158.5$ (dsept., **6**, $^1J_{FP} = 988$ Hz, $^3J_{FH} = 12$ Hz), -128.1 (d, $[Ph_3PF]^+$, $^1J_{FP} = 998$ Hz); **$^{31}P\{^1H\}$ NMR (CD_2Cl_2, [ppm]):** $\delta = -28.0$ (s, Ph_3PO), 94.8 (d, $[Ph_3PF]^+$, $^1J_{PF} = 998$ Hz), 147.4 (d, **6**, $^1J_{PF} = 988$ Hz).

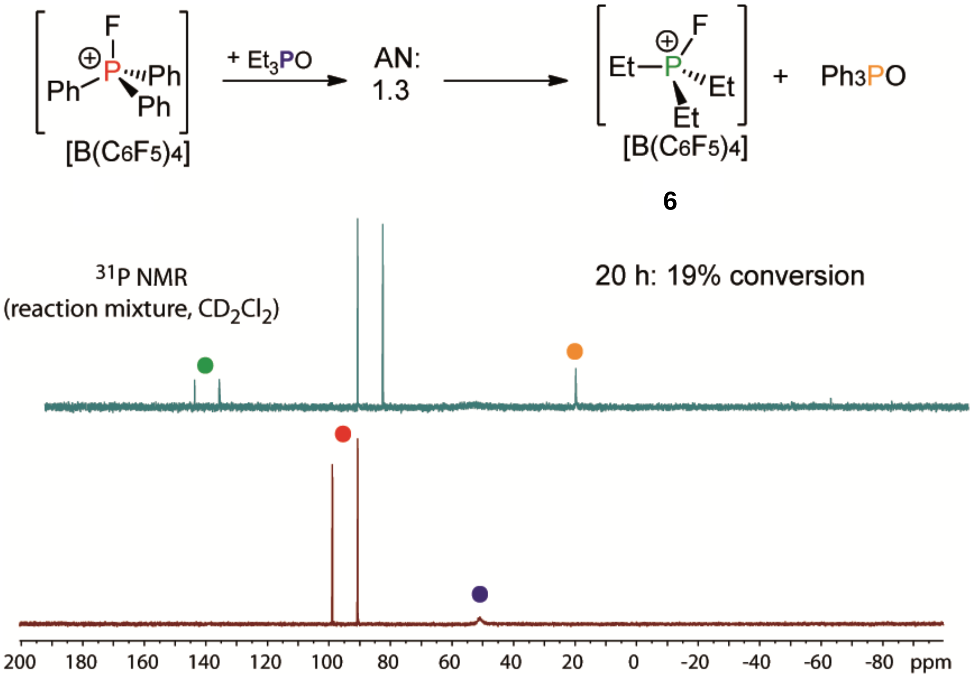

9a + Et_3PO (1 h):

$^{19}F\{^1H\}$ NMR (CD_2Cl_2, [ppm]): $\delta = -99.4$ (dm, **11**, $^1J_{FP} = 1014$ Hz); **$^{31}P\{^1H\}$ NMR (CD_2Cl_2, [ppm]):** $\delta = 74.1$ (s(br), **10**), 81.2 (dm), **11**, $^1J_{PF} = 1014$ Hz).

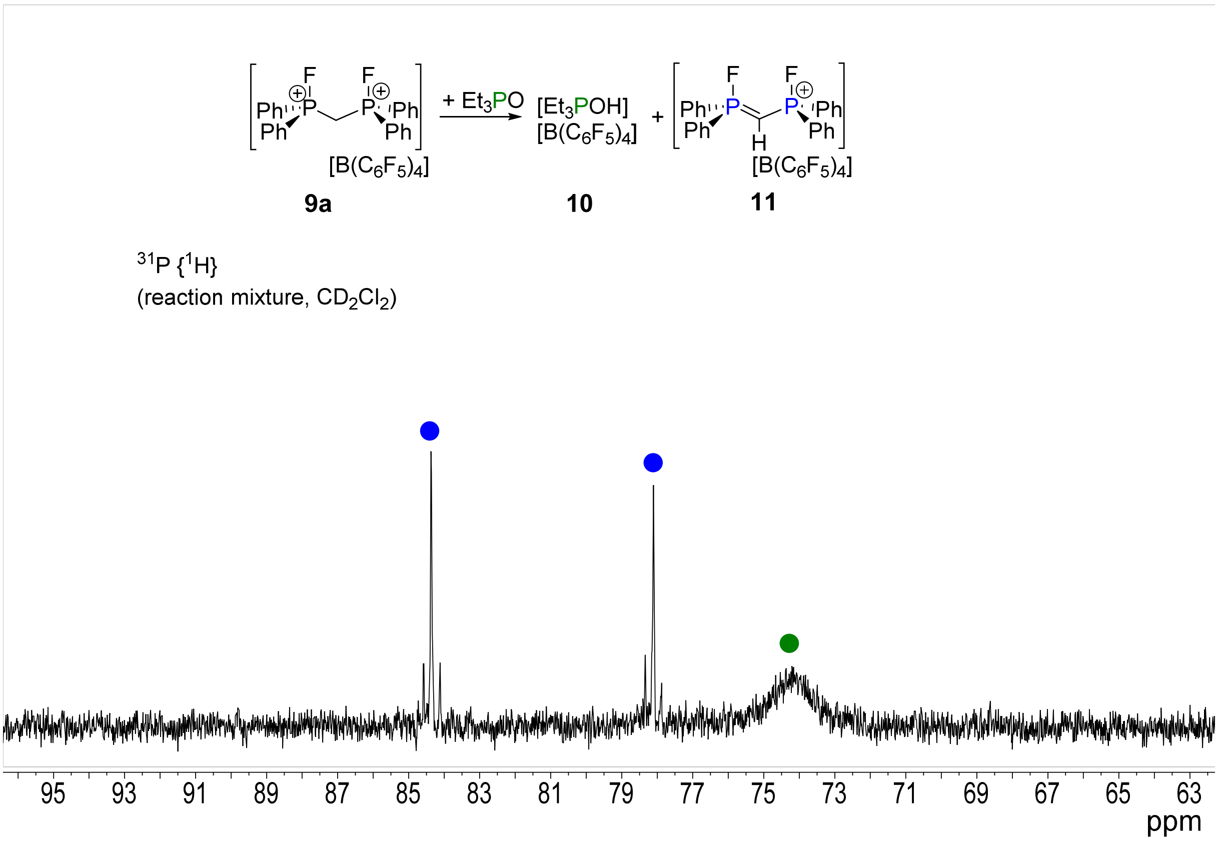

$^{31}P\{^1H\}$ NMR spectrum of the reaction of **9a** and Et_3PO (CD_2Cl_2).

Note:

The deprotonation of **9a** was independently investigated using *t*-Bu_3P as a base. Quantitative transformation to [*t*-$Bu_3PH]^+$ and **11** was observed according to ^1H, $^{19}F\{^1H\}$ and ^{31}P NMR spectroscopy.

9a + *t*-Bu_3P:

^{19}F NMR (CD_2Cl_2, [ppm]): $\delta = -99.6$ (ddm, **11**, $^1J_{FP} = 1013$ Hz, $^3J_{FH} = 5.6$ Hz); **$^{31}P\{^1H\}$ NMR (CD_2Cl_2, [ppm]):** $\delta = 81.5$ (dm, **11**, $^1J_{PF} = 1013$ Hz), 61.0 (s(br), [*t*-$Bu_3PH]^+$)

^{31}P NMR (CD_2Cl_2, [ppm]): $\delta = 81.5$ (d(br), **11**, $^1J_{PF} = 1013$ Hz), 61.0 (dm, $^1J_{PH} = 426$ Hz, [*t*-$Bu_3PH]^+$)

9b + Et_3PO (1 h):

^{19}F{^{1}H} NMR (CD_2Cl_2, [ppm]): $\delta = -159.1$ (d(br), **6**, $^{1}J_{FP} = 963$ Hz), -44.8 (d(br), **12b**, $^{1}J_{FP} = 720$ Hz); **^{31}P{^{1}H} NMR (CD_2Cl_2, [ppm]):** $\delta = 2.2$ (dd, **12b**, $^{1}J_{FP} = 720$ Hz, $^{3}J_{PP} = 7$ Hz), 53.1 (dd, **12b**, $^{3}J_{PP} = 7$ Hz, $^{4}J_{PF} = 2$ Hz), 147.4 (d, **6**, $^{1}J_{PF} = 963$ Hz).

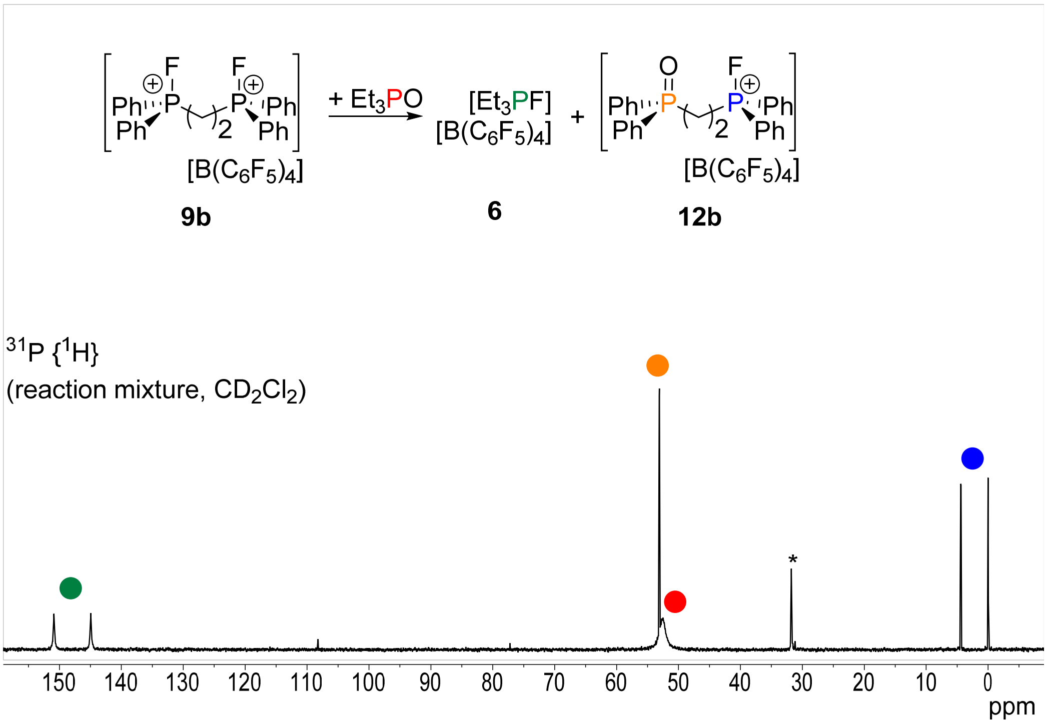

^{31}P{^{1}H} NMR spectrum of the reaction of **9b** and Et_3PO (CD_2Cl_2). * indicates some amounts of $(CH_2)_2(Ph_2PO)_2$.[S14]

9c + Et_3PO (1 h):

^{19}F{^{1}H} NMR (CD_2Cl_2, [ppm]): $\delta = -159.2$ (d, **6**, $^{1}J_{FP} = 967$ Hz), -86.4 (d, **12c**, $^{1}J_{FP} = 823$ Hz); **^{31}P{^{1}H} NMR (CD_2Cl_2, [ppm]):** $\delta = 44.05$ (d, **12c**, $^{4}J_{PP} = 12$ Hz), 45.2 (dd, **12c**, $^{1}J_{PF} = 823$ Hz, $^{4}J_{PP} = 12$ Hz), 148.0 (d, **6**, $^{1}J_{PF} = 967$ Hz).

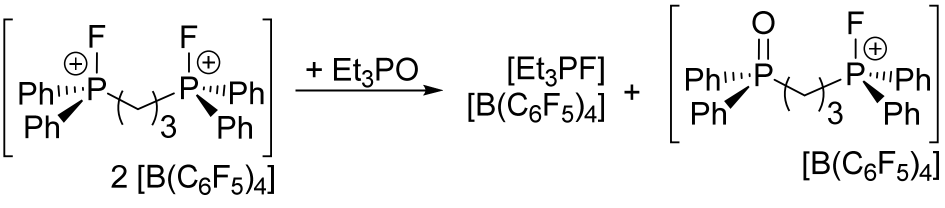

9c **6** **12c**

6

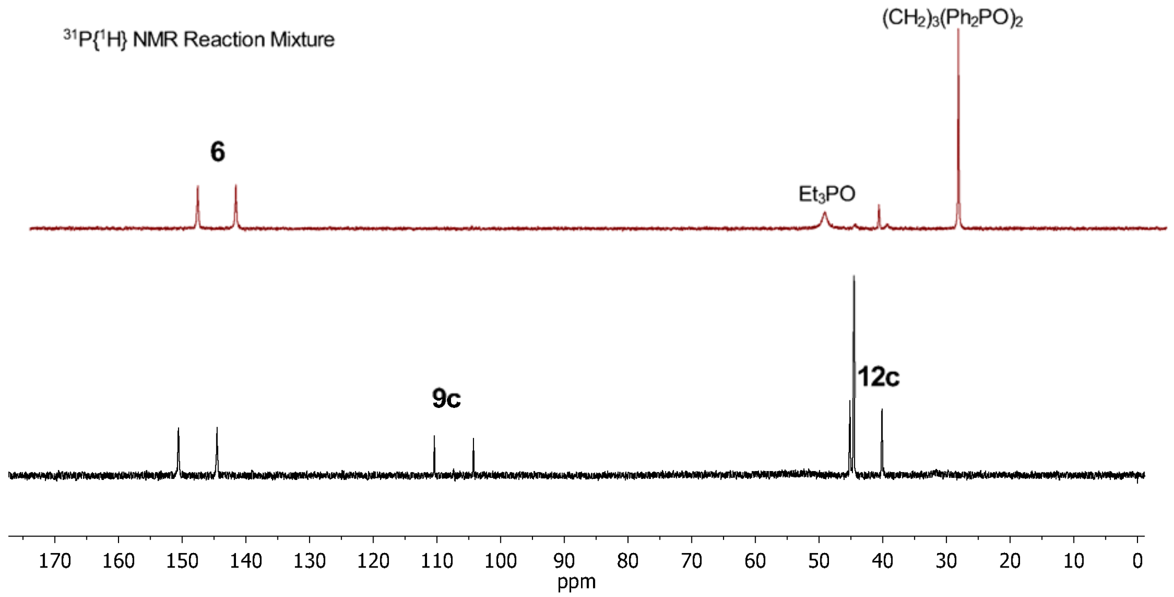

$^{31}P\{^1H\}$ NMR spectrum of the reaction of **9c** and Et_3PO (CD_2Cl_2).

9d + Et_3PO (1 h):

$^{19}F\{^1H\}$ NMR (CD_2Cl_2, [ppm]): $\delta = -139.6$ (dt, **9d**, $^1J_{FP} = 994$ Hz), **$^{31}P\{^1H\}$ NMR (CD_2Cl_2, [ppm]):** $\delta = 58.0$ (s(br), Et_3PO), 96.7 (dd, **9d**, $^1J_{PF} = 994$ Hz).

9d + Et_3PO (20 h):

$^{19}F\{^1H\}$ NMR (CD_2Cl_2, [ppm]): $\delta = -159.2$ (d(br), **6**, $^1J_{FP} = 969$ Hz), -136.2 (dt, **12d**, $^1J_{FP} = 990$ Hz), **$^{31}P\{^1H\}$ NMR (CD_2Cl_2, [ppm]):** $\delta = 33.1$ (s, **12d**), 109.6 (d, **12d**, $^1J_{PF} = 990$ Hz), 149.8 (d, **6**, $^1J_{PF} = 969$ Hz).

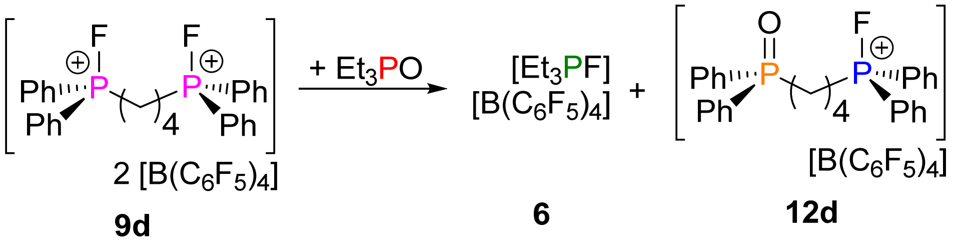

9d **6** **12d**

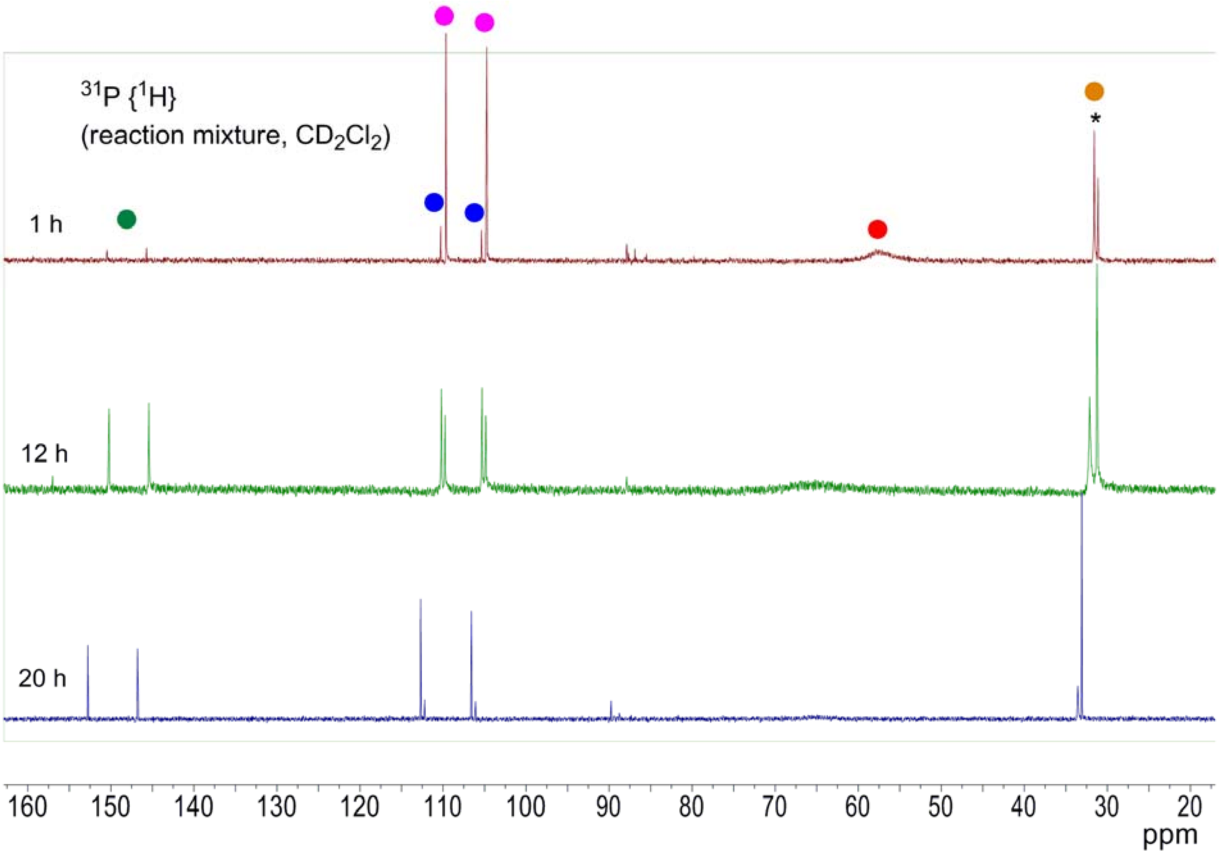

$^{31}P\{^{1}H\}$ NMR spectrum of the reaction of **9d** and Et_3PO (CD_2Cl_2). * indicates some amounts of $(CH_2)_4(Ph_2PO)_2$.

9e + Et_3PO (1 h):

$^{19}F\{^{1}H\}$ NMR (CD_2Cl_2, [ppm]): $\delta = -139.6$ (d, **9e**, $^{1}J_{FP} = 991$ Hz), -139.6 (d, **12e**, $^{1}J_{FP} = 991$ Hz); **$^{31}P\{^{1}H\}$ NMR (CD_2Cl_2, [ppm]):** $\delta = 31.8$ (d, **12e**), 52.8 (s(br), Et_3PO), 108.0 (d, **12e**, $^{1}J_{PF} = 991$ Hz), 107.2 (d, **9e**, $^{1}J_{PF} = 991$ Hz).

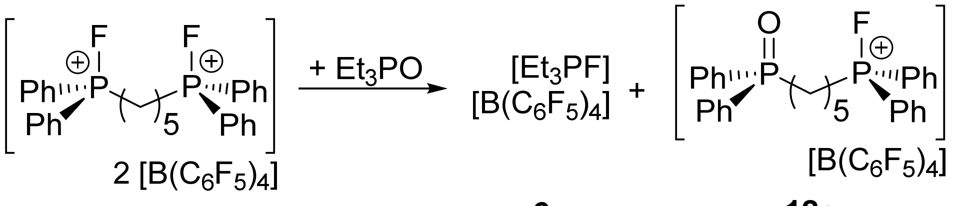

9e **6** **12e**

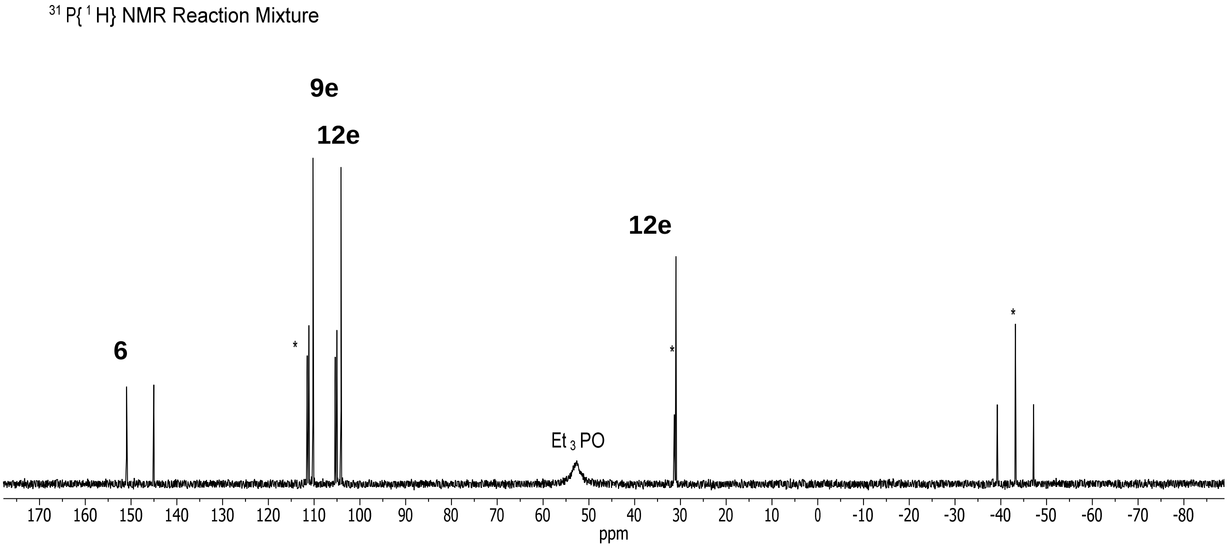

$^{31}P\{^{1}H\}$ NMR spectrum of the reaction of **9e** and Et_3PO (CD_2Cl_2). * indicates some amounts of $(CH_2)_5(Ph_2PO)_2$ and **8e**

2.21. Friedel-Crafts dimerization of 1,1-diphenylethylene with 5, $[Ph_3PF][B(C_6F_5)_4]$ and 9a-e as catalyst

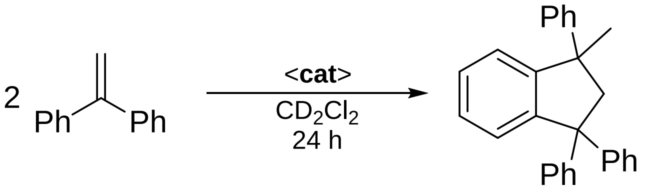

5 (2 mol%): 96% conversion (94% isolated yield)
$[Ph_3PF]^+$ (4 mol%): 1% conversion
9a (2 mol%): 99% conversion
9b (2 mol%): 99% conversion
9c (2 mol%): 99% conversion
9d (2 mol%): 50% conversion
9e (2 mol%): 25% conversion

Catalyst **5** (8 mg, 2 mol%), $[Ph_3PF][B(C_6F_5)_4]$ (8 mg, 4 mol%) or **9a-e** (6mg, 2 mol%) were added to a solution of 1,1-diphenylethylene (32 mg, 0.2 mmol) in CD_2Cl_2 (0.7 mL) at ambient temperature. The reaction mixture was left at ambient temperature for 24 h and investigated by

NMR spectroscopy. For catalyst **5**, all volatiles were removed *in vacuo* and the remaining residue was suspended in *n*-pentane. The mixture was filtered through a celite plug and the solvent was removed *in vacuo* giving 1-methyl-1,3,3-triphenyl-2,3-dihydro-1H-indene as a colourless solid (34 mg, 94% yield).[S8b]

^{1}H NMR (C_6D_6, [ppm]): δ = 1.48 (3H, s, CH_3), 3.02 (1H, d, CH_2, $^{3}J_{HH}$ = 13.5 Hz), 3.42 (1H, d, CH_2, $^{3}J_{HH}$ = 13.5 Hz), 6.90 - 7.23 (19H, m); **^{13}C{^{1}H} (C_6D_6, [ppm]):** δ = 29.1 (1C, s, CH_3), 51.5 (1C, s, CH_2), 61.4 (1C, s, CPh), 61.8 (1C, s, CPh), 125.4 (1C, s, Ph), 125.9 (1C, s, Ph), 126.0 (1C, s, Ph), 126.3 (1C, s, Ph), 127. 3 (1C, s, Ph), 127.3 (2C, s, Ph), 127.9 (2C, s, Ph), 128.0 (2C, s, Ph), 128.3 (2C, s, Ph), 128.3 (2C, s, Ph), 129.1 (2C, s, Ph), 129.3 (2C, s, Ph), 147.9 (1C, s, Ph), 149.1 (1C, s, Ph), 149.4 (1C, s, Ph), 149.7 (1C, s, Ph), 151.0 (1C, s, Ph).

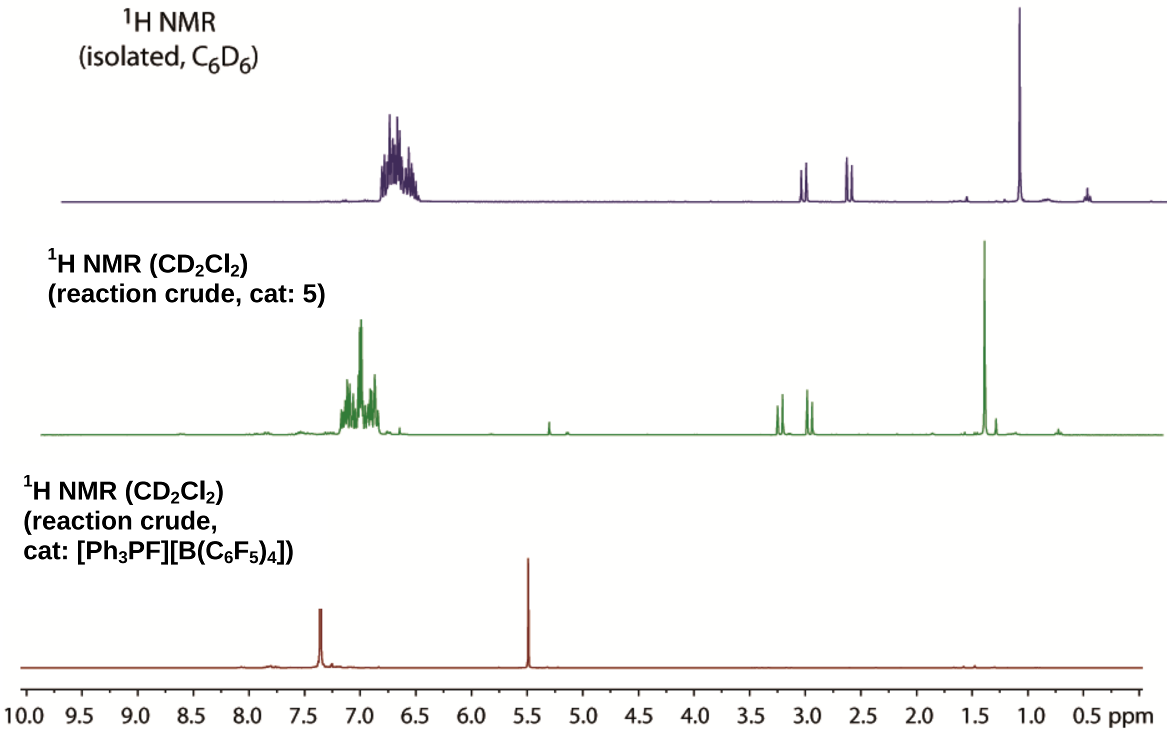

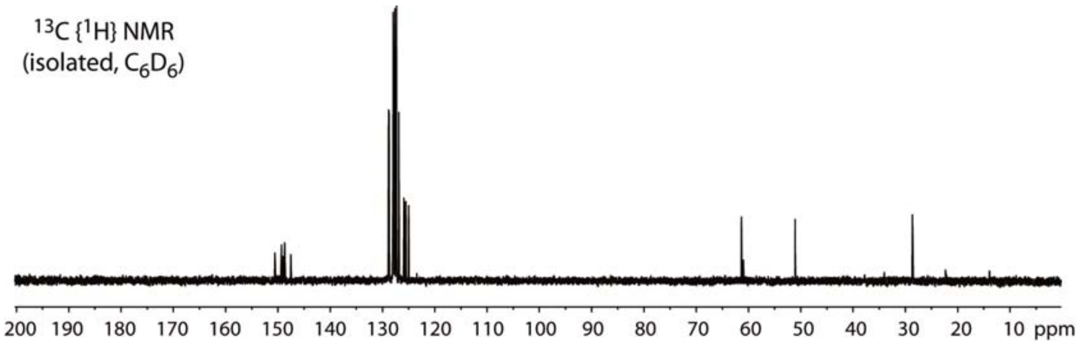

2.22 Dehydrocoupling of Et_3SiH and Phenol with 5, $[Ph_3PF][B(C_6F_5)_4]$ and 9a-e as catalysts.

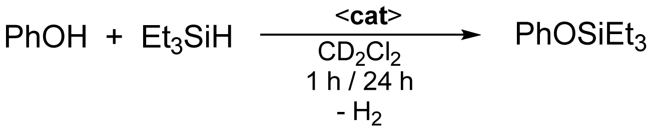

**5**	(2 mol%):	89% conversion (80% isolated yield)
**$[Ph_3PF]^+$**	(4 mol%):	< 1% conversion
**9a**	(2 mol%):	99% conversion
**9b**	(2 mol%):	85% conversion
**9c**	(2 mol%):	30% conversion
**9d**	(2 mol%):	19% conversion
**9e**	(2 mol%):	<1% conversion

Catalyst **5** (4 mg, 2 mol%), **9a-e** (6 mg, 2 mol%), or $[Ph_3PF][B(C_6F_5)_4]$ (4 mg, 4 mol%) were added to a solution of Et_3SiH (17 µL, 0.10 mmol) and PhOH (9 mg, 0.10 mmol) in CD_2Cl_2 (0.7 mL) at ambient temperature. The reaction mixture was left at ambient temperature for one hour (**5, 9a**) or 24 h ($[Ph_3PF]^+$) or heated to 50 °C for 24 h for **9b-e** and investigated by NMR spectroscopy. For catalyst **5**, all volatiles were removed *in vacuo* and the remaining residue was suspended in *n*-pentane. The mixture was filtered through a celite plug and the solvent was removed *in vacuo* giving triethyl(phenoxy)silane as a colourless oil (17 mg, 80% yield).[S14]

^{1}H NMR (C_6D_6, [ppm]): δ = 0.66 (6H, quart., CH_2, $^3J_{HH}$ = 7.8 Hz), 0.96 (9H, t, CH_3, $^3J_{HH}$ = 7.8 Hz), 6.85 (1H, t, *p*-Ph, $^3J_{HH}$ = 7.1 Hz), 6.89 - 6.94 (2H, m, *o-/m*-Ph), 7.07 - 7.15 (2H, m, *o-/m*-Ph); **$^{13}C\{^1H\}$ (C_6D_6, [ppm]):** δ = 5.4 (3C, s, CH_2), 6.9 (3C, s, CH_3), 120.3 (2C, s, *o-/m*-Ph), 121.7 (1C, s, *p*-Ph), 129.8 (2C, s, *o-/m*-Ph), 156.2 (1C, s, *i*-Ph).

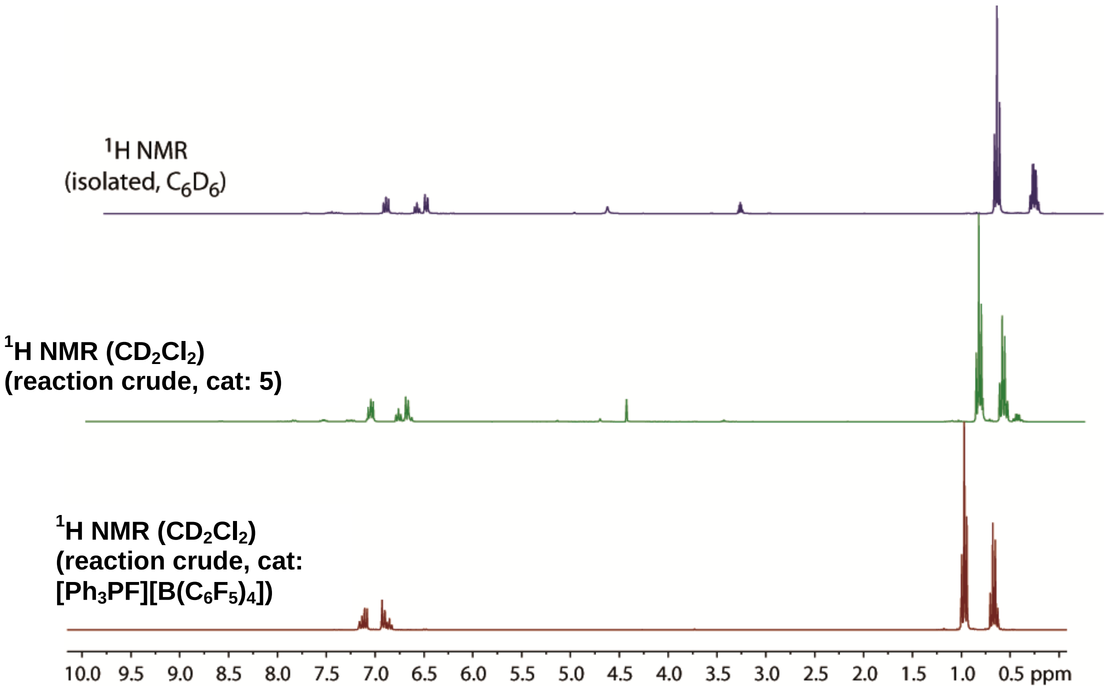
1H NMR
(isolated, C6D6)
1H NMR (CD2Cl2)
(reaction crude, cat: 5)
1H NMR (CD2Cl2)
(reaction crude, cat:
[Ph3PF][B(C6F5)4])
10.0 9.5 9.0 8.5 8.0 7.5 7.0 6.5 6.0 5.5 5.0 4.5 4.0 3.5 3.0 2.5 2.0 1.5 1.0 0.5 ppm

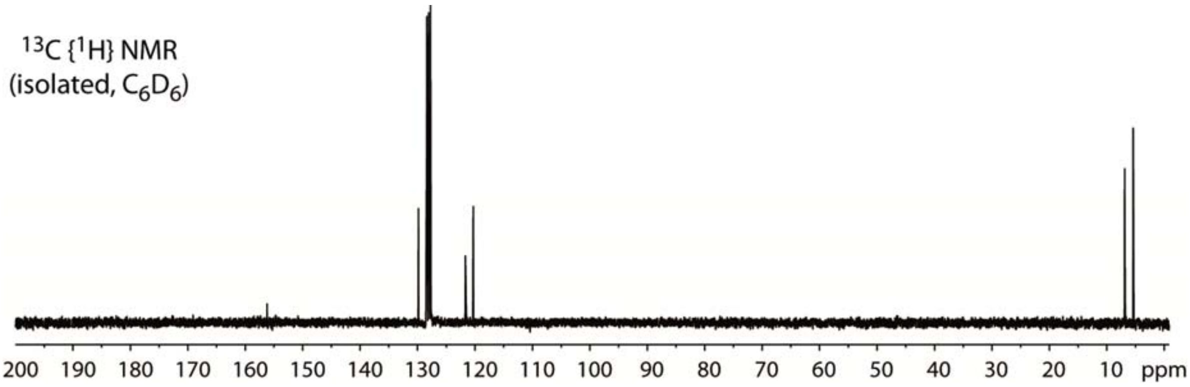
13C {1H} NMR
(isolated, C6D6)
200 190 180 170 160 150 140 130 120 110 100 90 80 70 60 50 40 30 20 10 ppm

2.23. Hydrosilylation of 1,1-diphenylethylene with Et_3SiH using 5, $[Ph_3PF][B(C_6F_5)_4]$ or 9a-e as catalysts

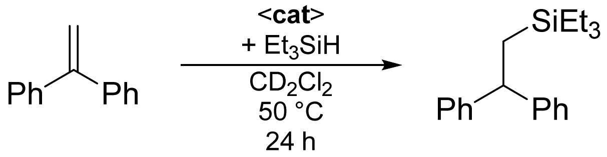

5 (2 mol%): 74% conversion (isolated yield 67%)
$[Ph_3PF]^+$ (4 mol%): 0% conversion
9a (2 mol%): 89% conversion (1h, R.T.)
9b (2 mol%): 72% conversion
9c-e (2 mol%): 0% conversion

Catalyst **5** (4 mg, 2 mol%), $[Ph_3PF][B(C_6F_5)_4]$ (4 mg, 4 mol%) or **9a-e** (6 mg, 2 mol%) were added to a solution of Et_3SiH (18 µL, 0.10 mmol) and 1,1-diphenylethylene (18 mg, 0.10 mmol) in CD_2Cl_2 (0.7 mL). The reaction mixtures were heated to 50 °C for 24 h and investigated by NMR spectroscopy. For catalyst **5**, all volatiles were removed *in vacuo* and the remaining residue was suspended in *n*-pentane. The mixture was filtered through a celite plug and the solvent was removed *in vacuo* giving (2,2-diphenylethyl)triethylsilane as a colourless oil (20 mg, 67% yield).[16]

^{1}H NMR (C_6D_6, [ppm]): $\delta = 0.33$ (6H, quart., $SiCH_2CH_3$, $^3J_{HH} = 7.8$ Hz), 0.83 (9H, t, $SiCH_2CH_3$, $^3J_{HH} = 7.9$ Hz), 1.34 (2H, s, CH_2, $^3J_{HH} = 7.9$ Hz), 4.03 (1H, t, CH, $^3J_{HH} = 7.9$ Hz), 6.94 - 7.00 (2H, m, *p*-Ph), 7.05 - 7.10 (4H, m, *m*-Ph), 7.17 - 7.21 (4H, m, *m*-Ph); **$^{13}C\{^1H\}$ (C_6D_6, [ppm]):** $\delta = 3.9$ (3C, s, $SiCH_2CH_3$), 7.7 (3C, s, $SiCH_2CH_3$), 19.4 (1C, s, CH_2), 47.6 (1C, s, CH), 126.3 (2C, s, *p*-Ph), 127.9 (4C, s, *m*-/*o*-Ph), 128.7 (4C, s, *m*-/*o*-Ph), 147.8 (2C, s, *i*-Ph).

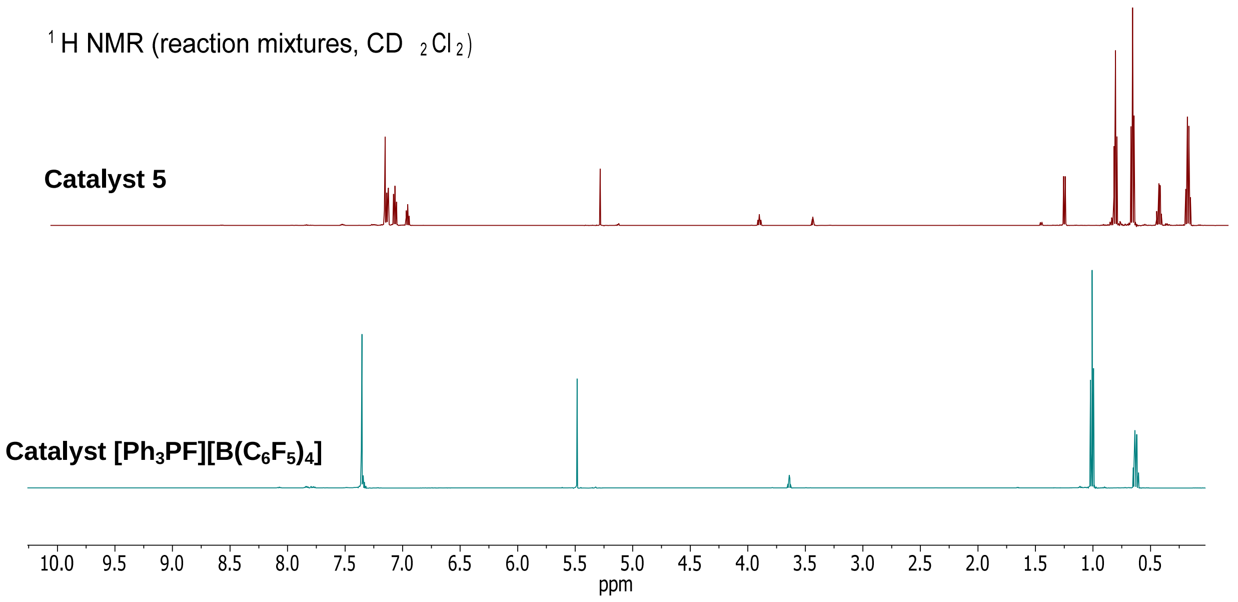
1H NMR (reaction mixtures, CD2Cl2)
Catalyst 5
Catalyst [Ph3PF][B(C6F5)4]
10.0 9.5 9.0 8.5 8.0 7.5 7.0 6.5 6.0 5.5 5.0 4.5 4.0 3.5 3.0 2.5 2.0 1.5 1.0 0.5
ppm

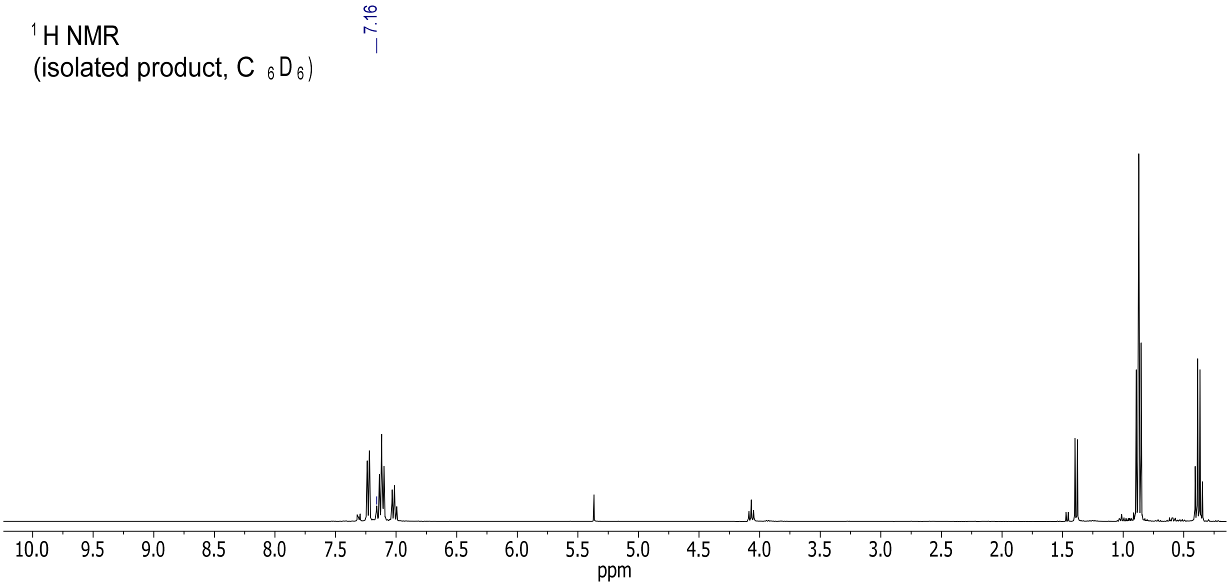
1H NMR
(isolated product, C6D6)
7.16
10.0 9.5 9.0 8.5 8.0 7.5 7.0 6.5 6.0 5.5 5.0 4.5 4.0 3.5 3.0 2.5 2.0 1.5 1.0 0.5
ppm

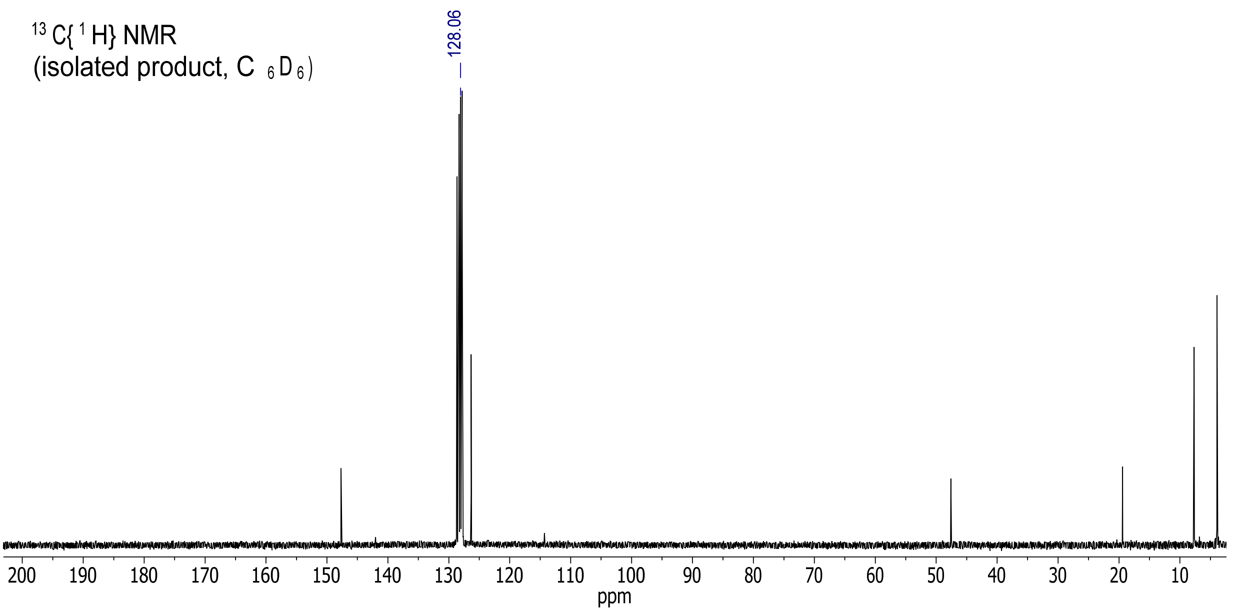

2.24. Hydrodeoxygenation of benzophenone in the presence of Et_3SiH using 5, $[Ph_3PF][B(C_6F_5)_4]$ and 9a-e as catalysts

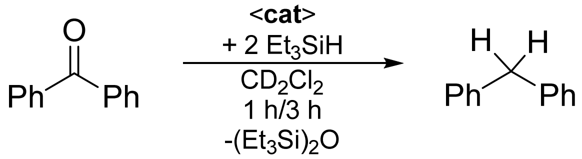

**5**	(2 mol%):	99% conversion (59% isolated yield)
**$[Ph_3PF]^+$**	(4 mol%):	0% conversion
**9a**	(2 mol%):	99% conversion
**9b**	(2 mol%):	99% conversion
**9c**	(2 mol%):	93% conversion
**9d**	(2 mol%):	72% conversion
**9e**	(2 mol%):	46% conversion

Catalyst **5** (4 mg, 2 mol%), $[Ph_3PF][B(C_6F_5)_4]$ (4 mg, 4 mol%) or **9a-e** (6 mg, 2 mol%) were added to a solution of Et_3SiH (18 µL, 0.10 mmol) and benzophenone (18 mg, 0.10 mmol) in CD_2Cl_2 (0.7 mL). The reaction mixtures were left at ambient temperature for one hour or heated to 50 °C for 36 h for **9c-e** and investigated by NMR spectroscopy. For catalyst **5**, all volatiles were removed *in vacuo* and the remaining residue was suspended in *n*-pentane. The mixture was purified by flash chromatography using a silica column (3 cm). The obtained *n*-pentane fraction

contained mainly $Et_3SiOSiEt_3$ and the product was obtained using Et_2O as an eluent. Removal of all volatiles *in vacuo* gave diphenylmethane as colourless oil (10 mg, 59% yield).

^{1}H NMR (C_6D_6 [ppm]): δ = 3.74 (2H, s, $PhCH_2$), 7.01 - 7.07 (6H, m, Ph), 7.09 - 7.14 (4H, m, Ph); **^{13}C{^{1}H} (C_6D_6, [ppm]):** δ = 42.2 (1C, s, CH_2), 126.3 (1C, s, *p*-Ph), 128.7 (2C, s, *o*-/*m*-Ph), 129.3 (2C, s, *o*-/*m*-Ph), 141.5 (1C, s, *i*-Ph).

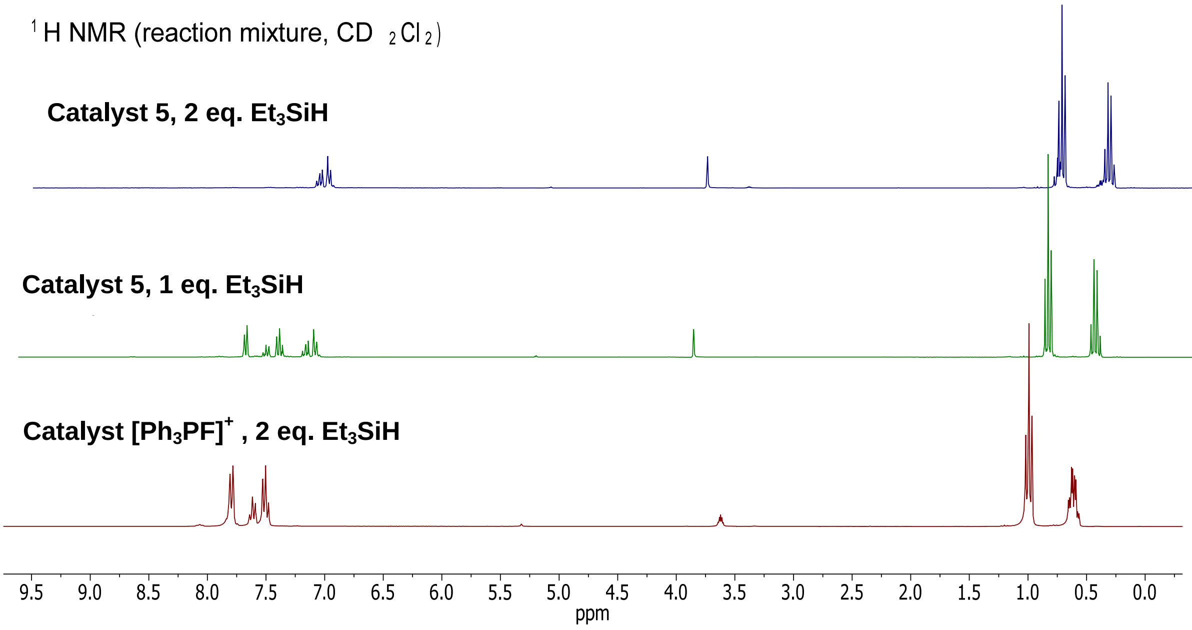

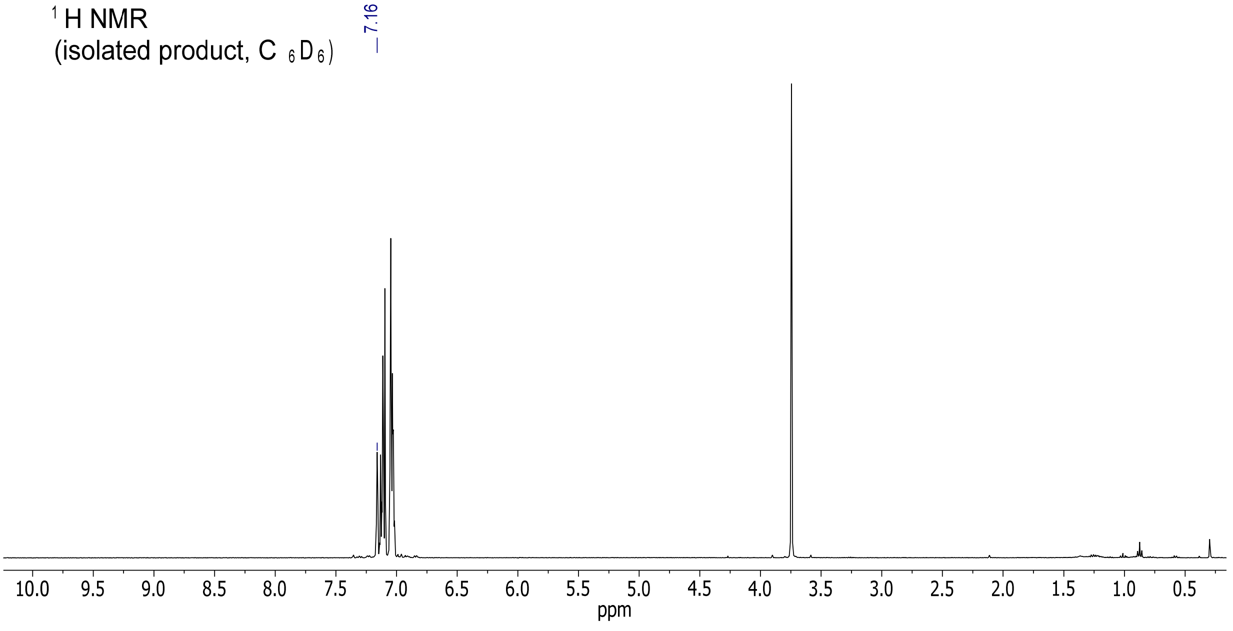

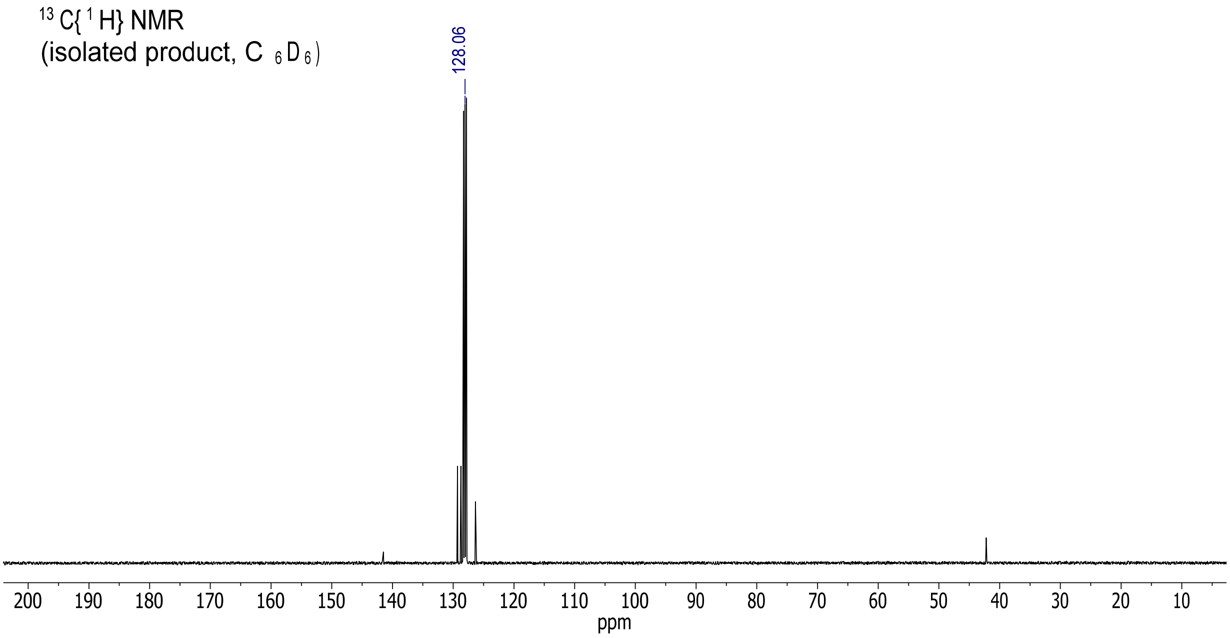

2.25. Hydrodefluorination of fluoropentane in the presence of Et_3SiH using 5, $[Ph_3PF][B(C_6F_5)_4]$ and 9a-e as catalysts

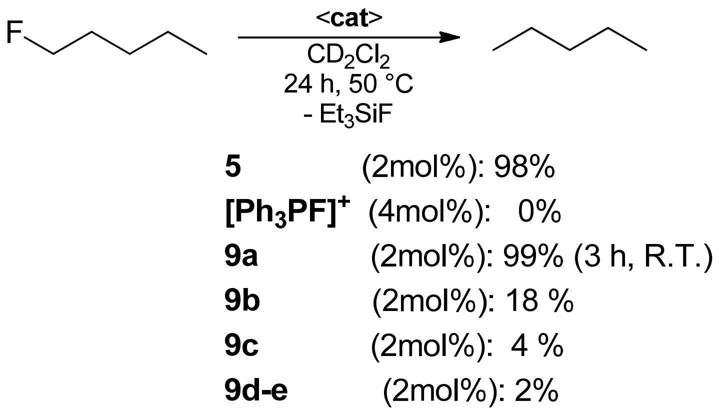

Catalyst **5** (4 mg, 2 mol%), $[Ph_3PF][B(C_6F_5)_4]$ (4 mg, 4 mol%) or **9a-e** (6 mg, 2 mol%) were added to a solution of Et_3SiH (21 µL, 0.12 mmol) and fluoropentane (12 µL, 0.10 mmol) in CD_2Cl_2 (0.7 mL). The reaction mixtures were heated to 50 °C for 24 h and investigated by NMR spectroscopy.[S15] Conversion was determined by means of ^{19}F NMR spectroscopy (Consumption of fluoropentane and formation of Et_3SiF).

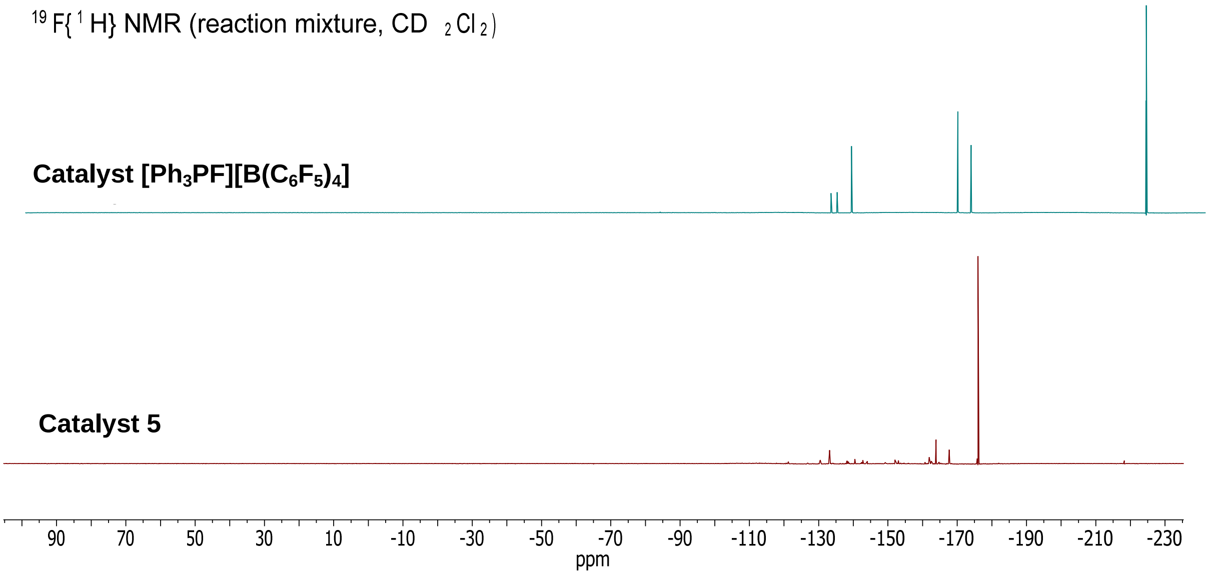
$^{19}F\{^{1}H\}$ NMR (reaction mixture, CD_2Cl_2)
Catalyst $[Ph_3PF][B(C_6F_5)_4]$
Catalyst 5
90 70 50 30 10 -10 -30 -50 -70 -90 -110 -130 -150 -170 -190 -210 -230
ppm

3. Crystallographic Details

Table 3.1. Crystallographic data and details of the structure refinements of compounds **2**, **3** and **4***(CH_2Cl_2)

	**2**	**3**	**4***(CH_2Cl_2)
formula	$C_{34}H_{26}BF_4P_2$	$C_{58}H_{26}BF_{21}P_2$	$C_{59.25}H_{28.50}BCl_{2.50}F_{23}P_2$
M_r [g mol^{-1}]	572.49	1194.54	1338.70
colour, habit	colourless, block	colourless, block	colourless, block
crystal system	monoclinic	monoclinic	monoclinic
Space group	$P2_1$/n	$P2_1$	$P2_1$/c
a [Å]	9.206(1)	8.426(1)	18.800(1)
b [Å]	16.745(1)	21.123(3)	17.137(1)
c [Å]	17.632(1)	13.804(2)	17.800(1)
α [°]	90	90	90
β [°]	101.286(3)	95.06(1)	102.564(2)
γ [°]	90	90	90
V [Å^3]	2655.6(3)	2447(1)	5597.6(5)
Z	4	2	4
T [K]	149(2)	150(2)	149(2)
Crystal size [mm]	0.20x0.20x0.20	0.20x0.10x0.10	0.40x0.20x0.20
ρ_c [g cm^{-3}]	1.427	1.621	1.589
F(000)	1184	1196	2674
θ_{min} [°]	1.69	1.48	1.63
θ_{max} [°]	27.55	27.46	27.55
Index range	$-11 \le h \le 11$<br>$-20 \le k \le 21$<br>$-22 \le l \le 22$	$-10 \le h \le 10$<br>$-27 \le k \le 27$<br>$-17 \le l \le 17$	$-24 \le h \le 24$<br>$-20 \le k \le 22$<br>$-22 \le l \le 23$
μ [mm^{-1}]	0.215	0.212	0.315
absorption correction	SADABS	SADABS	SADABS
reflections collected	24302	41120	50339
reflections unique	6136	10858	12911
R_{int}	0.079	0.0787	0.0441
reflection obs. [F>3σ(F)]	3558	7111	8842
residual density [e Å^{-3}]	0.663, –0.287	0.264, –0.266	0.419, –0.357
parameters	361	739	757
GOOF	1.011	0.983	0.989
R_1 [I>2σ(I)]	0.0602	0.0480	0.0458
wR_2 (all data)	0.1615	0.0852	0.1170
CCDC	1041558	1041560	1041561

Table 3.2. Crystallographic data and details of the structure refinement of compound **8a**.

	**8a**
formula	$C_{25}H_{22}F_4P_2$
M_r [g mol^{-1}]	460.37
color, habit	colourless, block
crystal system	monoclinic
Space group	*C*2
a [Å]	21.097(2)
b [Å]	8.388(1)
c [Å]	6.357(1)
α [°]	90
β [°]	102.383(3)
γ [°]	90
V [Å^3]	1098.7(2)
Z	2
T [K]	150(2)
Crystal size [mm]	0.20x0.20x0.10
ρ_c [g cm^{-3}]	1.391
F(000)	476
θ_{min} [°]	1.98
θ_{max} [°]	27.51
Index range	$-27 \le h \le 27$<br>$-10 \le k \le 10$<br>$-8 \le l \le 8$
μ [mm^{-1}]	0.242
absorption correction	SADABS
reflections collected	8224
reflections unique	2417
R_{int}	0.0247
reflection obs. [F>3σ(F)]	2243
residual density [e Å^{-3}]	0.275, –0.182
parameters	141
GOOF	1.048
R_1 [I>2σ(I)]	0.0277
wR_2 (all data)	0.0660
CCDC	1041559

4. References

[S1] D. F. Shriver, M. A. Drezdzon, *The manipulation of air sensitive compounds*, **1986**, Wiley VCH, New York, USA.

[S2] J. B. Lambert, S. Zhang, S. M. Ciro, *Organometallics* **1994**, 2430.

[S3] a) *SAINT 7.23A*, Bruker AXS, Inc: Madison, Wisconsin, **2006**; b) G. M. Sheldrick, *SADABS*, Bruker AXS, Inc.: Madison, Wisconsin, **2004**.

[S4] G. M. Sheldrick, *SHELXL-97, Program for crystal structure determination,* University of Göttingen, Germany, **1997**.

[S5] R. Appel, I. Ruppert, *Chem. Ber.* **1975**, *108*, 919.

[S6] I. Ruppert, V. Bastian, *Angew. Chem.* **1977**, *89*, 763; *Angew. Chem. Int. Ed. Engl.* **1977**, *16*, 718.

[S7] P. H. M. Budzelaar, *gNMR for Windows (5.0.6.0) NMR Simulation Program,* IvorySoft **2006**.

[S8] (*a*) C. B. Caputo, L. J. Hounjet, R. Dobrovetsky, D. W. Stephan, *Science* **2013**, *341*, 1374; (b) M. Holthausen, M. Mehta, D. W. Stephan, *Angew. Chem. Int. Ed.* **2014**, *53*, 6538.

[S9] (*a*) K.-O. Feldmann and J. J. Weigand, *J. Am. Chem. Soc.* 2012, **134**, 15443; (*b*) M. H. Holthausen, S. K. Surmiak, P. Jerabek, G. Frenking and J. J. Weigand, *Angew. Chem. Int. Ed.* 2013, **52**, 11078.

[S10] G. S. Reddy, R. Schmutzler, *Z. Naturforsch. B.* **1970**, *25*, 1199.

[S11] F. Seel, H.-J. Bassler, *Z. Anorg. Allg. Chem.* **1975**, *418*, 263.

[S12] G. E. Maciel, R. V. James, *Inorg. Chem.* **1964**, *3*, 1650.

[S13] W. Peng, J. M. Shreeve, *J. Fluorine Chem.* **2005**, *126*, 1054.

[S14] M. Pèrez, C. B. Caputo, R. Dobrovetsky, D. W. Stephan, *Proc. Natl. Acad. Sci. USA* **2014**, *111*, 10917.

[S15] M. Pèrez, L. J. Hounjet, C. B. Caputo, R. Dobrovetsky, D. W. Stephan, *J. Am. Chem. Soc.* **2013**, *135*, 18308.
